# Is increased time to diagnosis and treatment in symptomatic cancer associated with poorer outcomes? Systematic review

**DOI:** 10.1038/bjc.2015.48

**Published:** 2015-03-03

**Authors:** R D Neal, P Tharmanathan, B France, N U Din, S Cotton, J Fallon-Ferguson, W Hamilton, A Hendry, M Hendry, R Lewis, U Macleod, E D Mitchell, M Pickett, T Rai, K Shaw, N Stuart, M L Tørring, C Wilkinson, B Williams, N Williams, J Emery

**Affiliations:** 1 North Wales Centre for Primary Care Research, Bangor University, Bangor LL13 7YP, UK; 2 Department of Health Sciences, University of York, York, YO10 5DD, UK; 3 Betsi Cadwaladr University Health Board, Wrexham Maelor Hospital, Wrexham LL13 7TD, UK; 4 Primary Care Collaborative Cancer Clinical Trials Group, School of Primary, Aboriginal, and Rural Healthcare, University of Western Australia, M706, 35 Stirling Highway, Crawley, Western Australia 6009, Australia; 5 University of Exeter Medical School, Exeter EX1 2LU, UK; 6 Centre for Health and Population studies, Hull York Medical School, University of Hull, Hull HU6 7RX, UK; 7 Leeds Institute of Health Sciences, University of Leeds, Leeds LS2 9LJ, UK; 8 North Wales Organisation for Randomised Trials in Health, Bangor University, Bangor LL57 2PZ, UK; 9 School of Medical Sciences, Bangor University, Bangor, LL57 2AS UK; 10 Research Unit for General Practice, Aarhus University, Bartholins Alle 2, Aarhus DK-8000, Denmark; 11 General Practice & Primary Care Academic Centre, University of Melbourne, 200 Berkeley Street, Melbourne, Victoria 3053, Australia

**Keywords:** systematic review, diagnosis, delays, survival, stage

## Abstract

**Background:**

It is unclear whether more timely cancer diagnosis brings favourable outcomes, with much of the previous evidence, in some cancers, being equivocal. We set out to determine whether there is an association between time to diagnosis, treatment and clinical outcomes, across all cancers for symptomatic presentations.

**Methods:**

Systematic review of the literature and narrative synthesis.

**Results:**

We included 177 articles reporting 209 studies. These studies varied in study design, the time intervals assessed and the outcomes reported. Study quality was variable, with a small number of higher-quality studies. Heterogeneity precluded definitive findings. The cancers with more reports of an association between shorter times to diagnosis and more favourable outcomes were breast, colorectal, head and neck, testicular and melanoma.

**Conclusions:**

This is the first review encompassing many cancer types, and we have demonstrated those cancers in which more evidence of an association between shorter times to diagnosis and more favourable outcomes exists, and where it is lacking. We believe that it is reasonable to assume that efforts to expedite the diagnosis of symptomatic cancer are likely to have benefits for patients in terms of improved survival, earlier-stage diagnosis and improved quality of life, although these benefits vary between cancers.

Symptomatic diagnosis of cancer is important and has been the subject of considerable innovation and intervention in recent years to achieve timelier and earlier-stage diagnosis (Emery *et al*, 2014); the English National Awareness and Early Diagnosis Initiative has made a major contribution to this effort (Richards and Hiom, 2009; Richards, 2009a). We know that patients value timely diagnostic workup, and that later stage at diagnosis is one of the contributory factors to poor cancer outcomes (Richards, 2009b). However, it is less clear whether more timely cancer diagnosis brings favourable outcomes. Systematic reviews in breast cancer reported that delays of 3–6 months were associated with lower survival (Richards *et al*, 1999), and in colorectal cancer it was concluded that there were no associations between diagnostic delays and survival and stage (Ramos *et al*, 2007, 2008; Thompson *et al*, 2010). Other reviews have been published for gynaecological cancers (Menczer, 2000), bladder (Fahmy *et al*, 2006), testicular (Bell *et al*, 2006), lung (Jensen *et al*, 2002; Olsson *et al*, 2009), paediatric cancers (Brasme *et al*, 2012a, 2012b) and head and neck cancers (Goy *et al*, 2009; Seoane *et al*, 2012), all with equivocal findings. No review to date has undertaken this work in a range of different cancer types.

Longer time to diagnosis may be detrimental in several ways: a more advanced stage at diagnosis, poorer survival, greater disease-related and treatment-related morbidity and adverse psychological adjustment. Conversely, harm may be caused by earlier detection of cancers without improving survival (lead-time), and detection of slow-growing tumours not needing treatment (over-diagnosis) (Esserman *et al*, 2013). A scoping review, undertaken before the review reported here, showed that observational studies in many cancers reported no association or an inverse relationship between longer diagnostic times and better outcomes (Neal, 2009). We therefore undertook a systematic review of the literature aiming to determine whether there is an association between time to diagnosis, treatment and clinical outcomes, across all cancers for symptomatic presentations only.

## Materials and methods

We undertook a systematic review in two phases. The original review was conducted in 2008–10, and the literature from inception of databases to February 2010 was searched; the update was conducted in 2013–14, and the literature from February 2010 to November 2013 was searched. The original review did not include breast or colorectal cancer (because of prior systematic reviews); however, these were included in the update (as we knew of more papers published in these cancers). The review adhered to principles of good practice (Egger *et al*, 2001; NHS Centre for Reviews and Dissemination, 2001). Reporting is in line with the PRISMA recommendations (Moher *et al*, 2009).

**Figure 1:**
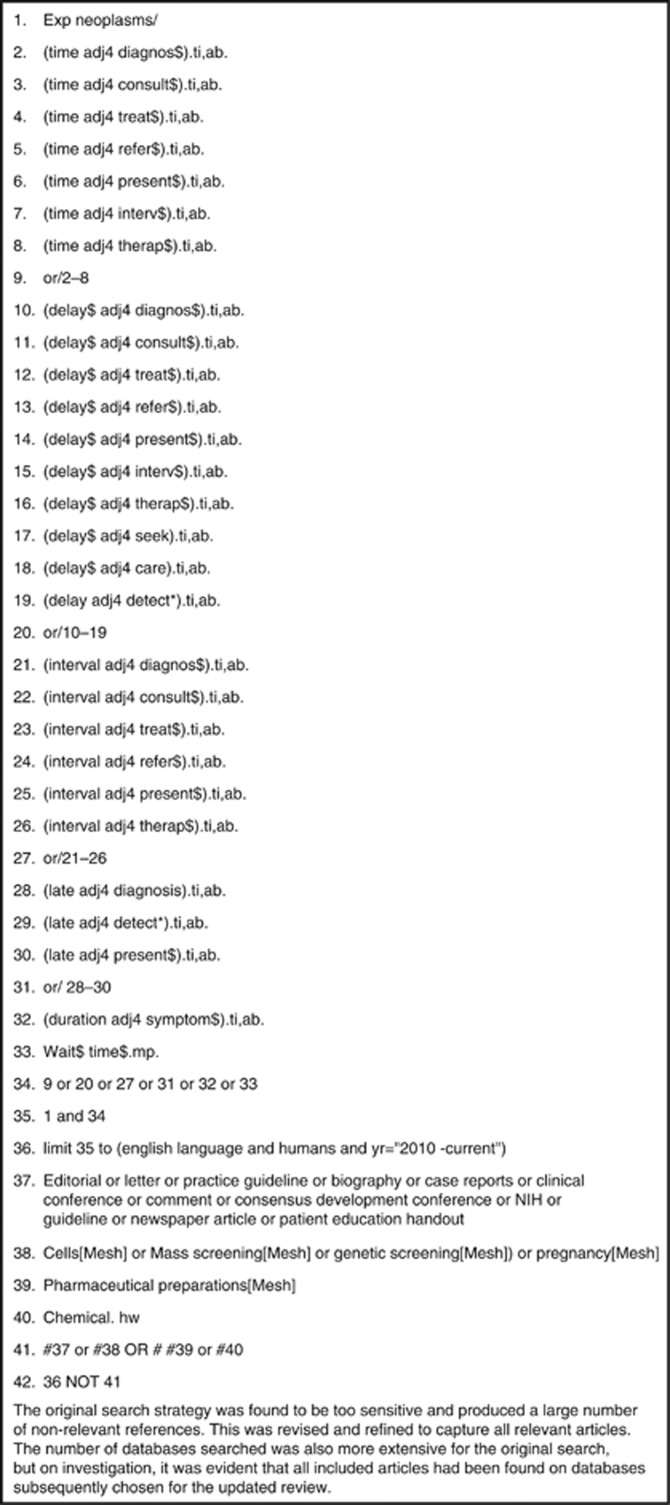
Search strategy (medline).

A search strategy was developed for Medline (Figure 1) and adapted for other search sources. A range of bibliographic databases were searched for relevant studies. These were as follows:

- MEDLINE, MEDLINE in-process, EMBASE, CINAHL, PsychINFO
- Cochrane Central Register of Controlled Trials, Database of Abstracts of Reviews of Effects, Cochrane Database of Systematic Reviews, Health Technology Assessment Database, NHS Economic Evaluation database.

Reference lists of studies included in this and previous reviews were hand-searched for relevant studies.

One reviewer screened the titles and abstracts of all records for relevance, and assessed potentially relevant records for inclusion. A second reviewer checked the decisions; disagreements were resolved by discussion or, if necessary, by a third reviewer. A study or analysis was included in the review if it:

- Reported patients with symptomatic diagnosis of primary cancer (screen- and biomarker-detected cancers were excluded).
- Primarily aimed to determine the association of at least one time interval to diagnosis or treatment (patient, primary care, secondary care or a combination), allowing assessment against accepted definitions (Weller *et al*, 2012). The outcomes of interest were any measure of survival or mortality; any description of stage, including extent or severity of disease at diagnosis and response to treatment; or quality of life.
- Was available as full text in English.

**Figure 2:**
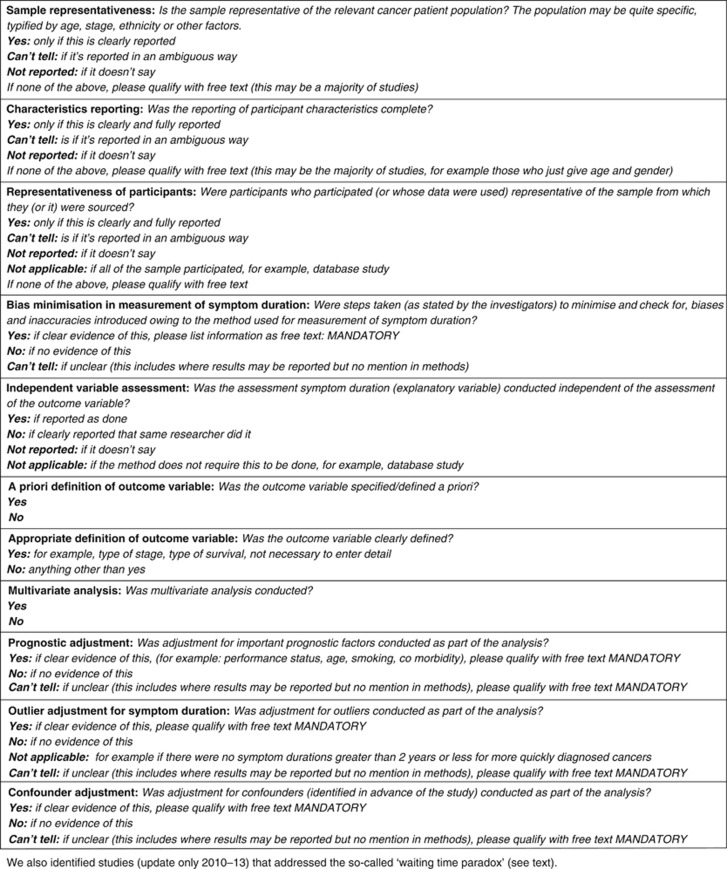
Bias assessment tool.

Data extraction for all included studies was done by one researcher and checked by another. We extracted data relating to the following:

- Characteristics of included studies: study aim, population, location, setting, definitions of time intervals, data collection methods used and outcome measures.
- Clinical outcomes: included the measure of association, associations of intervals with outcomes and reported interpretation.
- Bias assessment: we envisaged at the outset that there would be considerable variation between included studies in terms of study design, and that many may be of poor quality (Neal, 2009). We therefore considered that the assessment of methodological quality was especially important. However, at that time, there were no widely accepted checklists for checking the quality of prognostic studies, and there was little empirical evidence to support the importance of individual criteria, or study features, in affecting the reliability of study findings (Altman, 2001). Hence, we decided against the use of quality scoring, and to use a checklist instead of a scale. Judgements on the risk of bias were made according to a number of domains, using a generic list of questions within each domain (Figure 2), based primarily on a framework for assessing prognostic studies (Altman, 2001). For the updated review, and being aware of more recent literature on assessing the quality of prognostic studies, we decided to keep the original questions, as they were in line with the new Quality in Prognosis Studies tool (Hayden *et al*, 2006, 2013). In addition, in the update, we identified studies that addressed the so-called ‘waiting time paradox' (Crawford *et al*, 2002), which were likely to be of higher analytical quality. These were defined as follows: ‘articles that undertake an analysis or sub-analysis that specifically includes or excludes patients who are either diagnosed very quickly (e.g., within 4–8 weeks, although this will vary between cancers), or have very poor outcomes (e.g., deaths within a short time after diagnosis, e.g., within 4–8 weeks).' Agreement on inclusion in this subset of articles was done by two members of the study team. This is the ‘paradox' caused by the inclusion of patients with aggressive disease who invariably present early and have poor outcomes as a result of the aggressive disease, and is a form of confounding by indication.
- Clinical outcomes: the measure of association, associations of intervals with outcomes and interpretation.

We planned to undertake meta-analysis if there were sufficient homogenous studies reporting a similar outcome measure and the same interval for an individual cancer. Narrative synthesis was undertaken otherwise.

## Results

### Study selection

**Figure 3:**
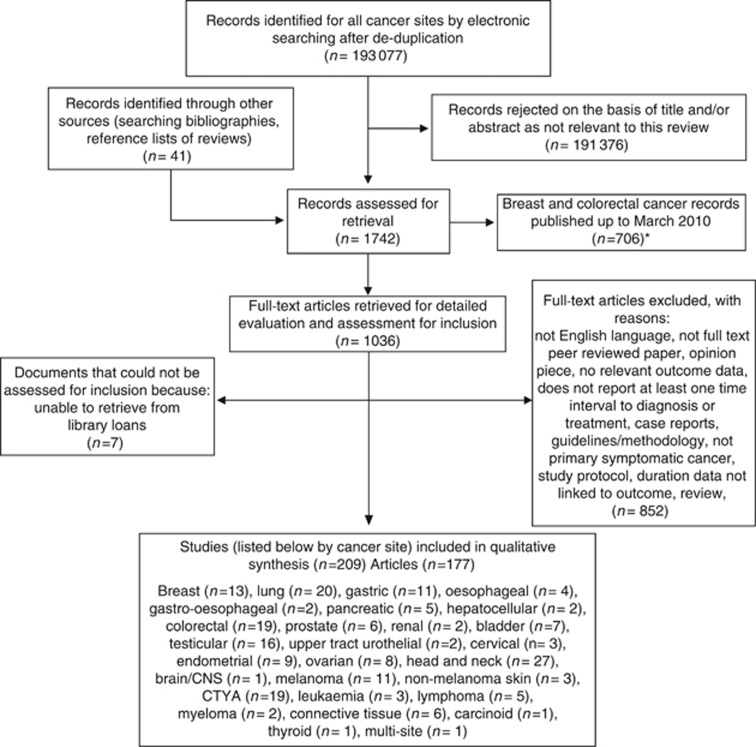
**Flow diagram.** *Of those breast and colorectal cancer records published up to March 2010 (*n*=706) assessed for retrieval, 330 were retrieved and assessed for inclusion but were not included in the evaluation, as systematic reviews on these cancers had been recently published. The follow-up review, covering the period March 2010 to October 2013, included both breast and colorectal cancers in the qualitative synthesis.

The number of studies screened, assessed for eligibility, included and reasons for exclusion are shown in Figure 3. Of the 1036 records identified for full-text review, 177 articles, reporting 209 studies, met the inclusion criteria and entered the narrative synthesis. A number of the articles reported data on more than one cancer, or more than one interval.

### Data collection in the included studies

#### Definition of time intervals

**Figure 4:**
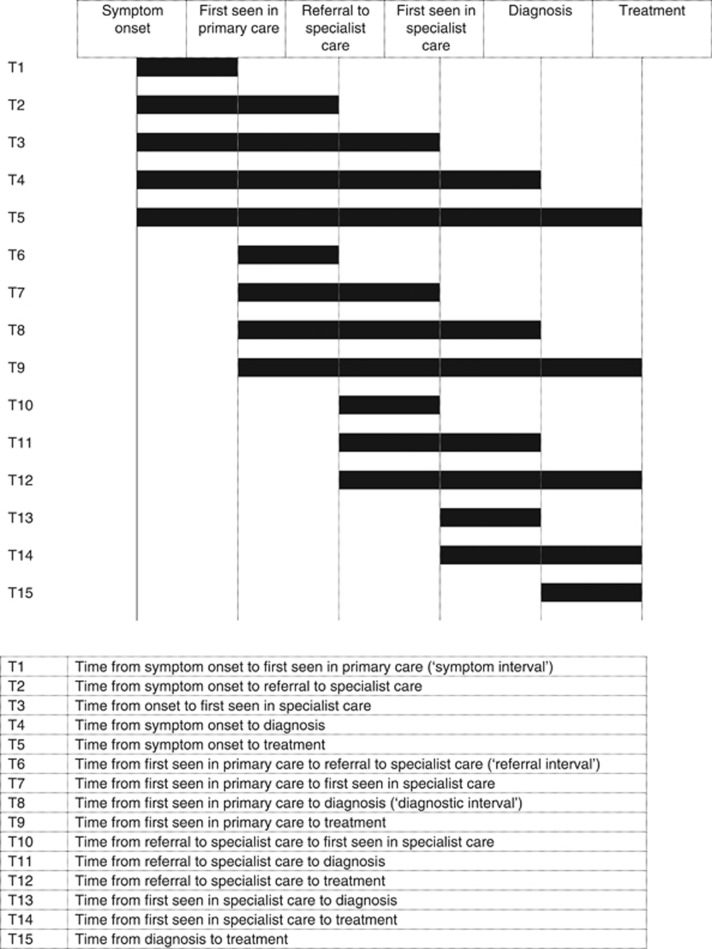
Definitions of time interval.

There were 15 different intervals reported in the included studies (Figure 4).

#### Clinical and psychological outcomes

Data collection for the outcome measures was predominantly retrospective review of medical records (using a variety of the following: clinical, pathological, histological and imaging) and cancer registries.

Patient questionnaires were used for studies with psychological outcomes. Most studies used various measures of survival (or mortality) and/or stage as outcome measures.

### Bias assessment

The bias assessment demonstrates the mixed quality of the studies (Supplementary Online Material). On a positive note, the characteristics and representativeness of the samples were reported in most articles, the definitions and appropriateness of time intervals were well reported and many studies undertook multivariable analysis. However, the representativeness of the samples was not reported in many articles, and few studies undertook confounder adjustment, prognostic adjustment or attempted bias minimisation. Only seven of the articles made an attempt to address the waiting time paradox (Tørring *et al*, 2011, 2012, 2013; Brasme *et al*, 2012a, 2012b; Elit *et al*, 2013; Gobbi *et al*, 2013; Pruitt *et al*, 2013). Hence, most studies failed to address the premise of confounding by indication–that is, the relationship between the diagnostic pathway (and hence the time interval) and prognosis.

### Study characteristics

Of the 177 articles included, there were a total of 401 760 participants, with a range of 13 to 147 682 in individual study size (Supplementary Online Material). There were 88 European studies with 23 from the UK, 9 from Italy, 8 from Spain, 8 from the Netherlands, 7 from Denmark, 7 from Finland, 5 from France, 5 from Norway, 3 from Switzerland, 3 from Sweden, 3 from Germany, 2 from Poland and 1 each from Austria, Belgium, Romania, Greece and joint UK/Denmark. There were 18 studies from Asia, with 5 from India, 4 from Japan, 4 from China, 2 from Hong Kong, 2 from Malaysia and 1 from South Korea. There were 59 studies from the Americas, with 47 studies from the USA, 8 from Canada and 4 from Brazil. In addition, there were three from Turkey, two from Israel, two from Australia, one each from New Zealand, Saudi Arabia, Libya, South Africa and one unspecified.

148 were based in specialist care 148 (106 single site, 38 multisite and 4 unspecified), 21 were population based, 3 were set in primary care, 3 database studies, 1 used hospital cancer registry data and 1 was unspecified. Study design varied hugely, and it included prospective and retrospective cohort studies, reviews of medical records, database analyses, patient surveys and interviews. The majority of the studies had retrospective designs.

### Synthesis of main findings

The results of individual studies are presented in Supplementary Online Material. No meta-analyses were possible. The results are reported cancer by cancer. Studies are grouped under ‘children teenagers and young adults' where they reported at least a significant proportion of participants aged <25 years.

Summaries for each cancer are reported in Table 1. Studies that reported ‘positive' associations (i.e., where there was evidence of shorter intervals being associated with more favourable outcomes) are presented first, followed by studies that reported no associations, followed by those that reported ‘negative' associations (i.e., where there was evidence of shorter intervals being associated with less favourable outcomes). In each section, studies reporting survival outcomes (or mortality, but for simplicity just referred to as survival in the table) are presented before those reporting stage and other outcomes. A brief narrative for each cancer is provided below.

For breast cancer, four studies reported positive associations, including one of the studies that addressed the waiting time paradox, and was able to demonstrate the effect of different diagnostic intervals on mortality (Tørring *et al*, 2013). The remainder reported no associations.

The lung studies had mixed findings, with similar numbers of studies reporting positive, negative and no associations, across a range of different time intervals. However, one of the studies reporting a positive association with mortality for diagnostic intervals addressed the waiting time paradox (Tørring *et al*, 2013).

For colorectal cancer, although many studies reported no associations, more studies reported a positive, rather than a negative, association. Indeed, four studies addressing the waiting time paradox were included, three of which reported a positive association (Tørring *et al*, 2011, 2012, 2013) and one a negative association (Pruitt *et al*, 2013). Of the upper gastrointestinal cancers, most studies reported no association, and more reported a negative, rather than a positive, association. For pancreatic cancer, two of the five studies reported a positive association, one of which addressed the waiting time paradox (Gobbi *et al*, 2013). The other three studies reported no association.

Two of the prostate studies reported a positive association for survival/mortality, one of which addressed the waiting time paradox (Tørring *et al*, 2013); the others reported no association. Two of the bladder studies reported a positive association; the others reported no association. For testicular cancer, 15 studies reported positive associations, and the remainder had no associations.

For gynaecological cancers, of the four studies examining cervix, one reported a positive association; the others reported no association. For endometrial and ovarian cancers, there were similar numbers of studies with positive, negative and no associations. One of the endometrial studies that reported a negative association addressed the waiting time paradox (Elit *et al*, 2013).

For head and neck cancers (pharyngeal, laryngeal, oral and others), there were a large number of studies and these were equally divided between those reporting a positive association and those reporting no association. No studies reported a negative association.

For melanoma, eight studies reported positive associations, one of which addressed the waiting time paradox (Tørring *et al*, 2013); the remainder reported no associations. For non-melanoma skin, two studies reported positive associations and one reported no association.

There were a large number of studies covering the various cancers in children, teenagers and young adults. The findings of these were very mixed, with the biggest group showing no associations, and smaller but similar number of studies reporting both positive and negative associations. One of the ‘no association' studies addressed the waiting time paradox (Brasme *et al*, 2012a, 2012b).

For lymphoma, three studies reported no association or a negative association. For leukaemia, the three studies reported no associations. There were only two studies in myeloma, although both of these reported positive outcomes. For the various connective tissue cancers, three studies each reported a positive association and no association. The other cancer groups (brain/central nervous system, carcinoid, hepatocellular, renal, thyroid, upper tract urothelial carcinoma and multisite) only had one or two included studies.

## Discussion

### Summary of main findings

This review is unique in that it has assessed the literature for a range of different cancer types, and hence we are able to make recommendations for policy practice and research that are not limited to one cancer (or group of cancers). The number of included studies in this review has shown the importance of this question to patients, clinicians and researchers. However, even within specific cancer types, there is only moderate consensus as to the nature of any associations between various time intervals in the diagnostic process and clinical outcomes, with some studies showing no associations, some studies showing better outcomes with shorter time intervals and some the opposite. There are more reports of an association between times to diagnosis and outcomes for breast, colorectal, head and neck, testicular and melanoma, with reports from a smaller number of studies for pancreatic, prostate and bladder cancers. The time intervals in the studies varied, making it impossible to draw consensus as to which intervals may be more, or less, important. Moreover, the methodological quality of many of these papers is mixed, despite a recent consensus paper on design and reporting of such studies (Weller *et al*, 2012). There is some evidence from papers published more recently that address the waiting time paradox in their analyses (Tørring *et al*, 2011, 2012, 2013; Brasme *et al*, 2012a, 2012b; Elit *et al*, 2013, Gobbi *et al*, 2013, Pruitt *et al*, 2013), with most, but not all, of these reporting longer intervals being associated with poorer outcomes, particularly mortality. This is important and begins to provide more robust evidence about the relationship between time to diagnosis and outcomes.

### Findings within the context of the literature

The previous cancer-specific reviews (Menczer, 2000; Jensen *et al*, 2002; Bell *et al*, 2006; Fahmy *et al*, 2006; Ramos *et al*, 2007, 2008; Goy *et al*, 2009; Olsson *et al*, 2009; Thompson *et al*, 2010; Brasme *et al*, 2012a, 2012b), with the exception of the breast cancer (Richards *et al*, 1999), and to a lesser extent head and neck (Seoane *et al*, 2012), have been largely equivocal, probably because of the poor quality of the included studies. Our findings are largely in keeping with these reviews, although we have provided much more evidence than previous reviews for testicular cancer (Bell *et al*, 2006) and head and neck cancers (Goy *et al*, 2009). We have also identified more recent and probably higher-quality papers providing better evidence for colorectal cancer than covered in previous reviews (Ramos *et al*, 2007, 2008; Thompson *et al*, 2010). We provide review findings for the first time for many cancers. We are also aware of further articles being published since the end date of our review. For example, one of these replicated the methods of one of the papers in our review (Tørring *et al*, 2011) on a sample of 958 colorectal cancers in Scotland, and reported that longer diagnostic intervals did not adversely affect cancer outcomes (Murchie *et al*, 2014). Another has reported that time to diagnosis in 436 Ewing tumours in France was not associated with metastasis, surgical outcome or survival (Brasme *et al*, 2014). One of our main findings, of the poor quality of reporting of time to diagnosis studies, replicates the findings of a recent paediatric systematic review (Launay *et al*, 2013).

### Strengths and weaknesses

This is the largest and most comprehensive review in this field, and the first ‘all-cancer' systematic review. The huge heterogeneity in both the outcomes and the time intervals used, within each cancer site, precluded meta-analyses. Another systematic review has recently reported similar difficulty in comparisons between studies (Lethaby *et al*, 2013). As previously stated, the review only contains studies in colorectal and breast cancer for 2010–13, and only these studies identified during the second round of searches were assessed to determine whether their analyses addressed the waiting time paradox. Survival, or mortality, is the most objective outcomes for these studies. However, many of the included studies in the review reported stage, or some other proxy. This may explain why stage and survival outcomes differ. Stage categorisation also varied, and some of the studies may be affected by *post-hoc* upstaging. A further problem with the literature is that of confounding by indication. Symptoms of more advanced cancer are likely to present differently and be investigated more promptly, as are patients presenting with so-called ‘red-flag' symptoms. We were unable to assess for publication bias; indeed, if there was any publication bias, we cannot predict in which direction this would act.

### Implications for policy, practice and research

Our main conclusion from this review is that we believe that it is reasonable to assume that efforts to expedite the diagnosis of symptomatic cancer are likely to have benefits for patients in terms of earlier-stage diagnosis, improved survival and improved quality of life. The amount of benefit varies between cancers; at present, there is more evidence for breast, colorectal, head and neck, testicular and melanoma, with evidence from a smaller number of studies for pancreatic, prostate and bladder cancers. There is either insufficient evidence or equivocal findings in the other cancers. The findings need replicating in using similar analytical methods, ideally also to address how much of a difference expedited diagnosis of different cancers would make on outcomes, and at which points in the diagnostic journey matters most. Until we have well-designed and well-analysed prospective studies to answer this question, it is difficult to determine the likely effect of interventions to reduce patient and diagnostic intervals on outcomes. This knowledge would inform the development of targeted intervention studies, to improve outcomes.

Hence, we recommend that policy, and clinicians, should continue the current emphasis on expediting symptomatic diagnosis, at least for most cancers. This can be achieved by clinicians having a high index of suspicion of cancer, the use of diagnostic technologies and rapid access to diagnostic investigations and fast-track pathways for assessment (Rubin *et al*, 2014). Finally, we recommend the need for more high-quality research in the area for a number of reasons. First, we suspect that many clinicians continue to believe that there are no associations between time and clinical outcomes.

A considerable number of studies fail to address basic issues of bias and thus equate the absence of evidence with evidence of absence. Second, it is likely that more timely diagnosis may have a greater or lesser impact between different cancers. This is important to ascertain, because it will inform policy and practice. We recommend, where possible, re-analysis of pooled (and similar) data from some of the studies included in this review, and new studies using linked data sets, across all cancers, such that similar analyses can be conducted between cancers. We also recommend that such studies should ideally focus on survival or mortality as the outcome, as this is the ‘gold-standard' outcome, although stage is also a valuable end point. There is also a dearth of studies reporting patient experience; we therefore recommend further work that examined the relationship between patient perceptions of ‘delay' and quality of life and psychological outcomes. Suggested key quality criteria for future studies are summarised in Box 1. Other work should focus on the organisation and function of health services, and subsequent time intervals and outcomes. Furthermore, we recommend that, wherever possible, this work should be conducted and reported in keeping within the recommendations of the Aarhus Statement (Weller *et al*, 2012).

## Figures and Tables

**Table 1:** Summary results from narrative synthesis, by cancer

**Positive association**	**No association**	**Negative association**
**Breast**		
**Survival** Diagnostic interval (Tørring *et al*, 2013) Treatment interval (Yun *et al*, 2012; Smith *et al*, 2013)	**Survival** Treatment interval (Brazda *et al*, 2010; McLaughlin *et al*, 2012; Eastman *et al*, 2013; Mujar *et al*, 2013; Redaniel *et al*, 2013; Sue *et al*, 2013)	None reported
		
**Stage** Symptom onset to diagnosis (Ermiah *et al*, 2012; Warner *et al*, 2012)	**Stage** Treatment interval (Wright *et al*, 2010; Wagner *et al*, 2011)	
		
	**Other outcomes** Treatment interval and risk of recurrence (Eastman *et al*, 2013)	
**Lung**		
**Survival** Diagnostic interval (Tørring *et al*, 2013) Symptom onset to diagnosis (Maguire *et al*, 1994)	**Survival** Symptom onset to treatment (Annakkaya *et al*, 2007) Patient interval (Loh *et al*, 2006) Diagnostic interval (Loh *et al* 2006; Pita-Fernandez *et al*, 2007; Skaug *et al*, 2011) Treatment interval (Diaconescu *et al*, 2011; Yun *et al*, 2012) Symptom onset to being seen in specialist care (Garcia-Barcala, 2012)	**Survival** Patient interval (Radzikowska *et al*, 2012) Treatment interval (Gonzalez-Barcala *et al*, 2010)
		
**Stage** Symptom onset to treatment (Christensen *et al*, 1997) Treatment interval (Brocken *et al*, 2012; Murai *et al*, 2012)	**Stage** Patient interval (Yilmaz *et al*, 2008; Tokuda *et al*, 2009) Diagnostic interval(Pita-Fernandez *et al*, 2007; Yilmaz *et al*, 2008)	**Stage** Diagnostic interval (Gould *et al*, 2008) Treatment interval (Salomaa *et al*, 2005) Symptom onset to treatment (Myrdal *et al*, 2004) Referral interval (Neal, 2007) First seen in secondary care to diagnosis (Brocken *et al*, 2012)
		
	**Other outcomes** Symptom onset to diagnosis and quality of life (Mohan *et al*, 2006)	
**Gastric**		
None reported	**Survival** Treatment interval (Yun *et al*, 2012) Symptom onset to diagnosis (Maguire *et al*, 1994; Martin *et al*, 1997; Windham *et al*, 2002; Arvanitakis *et al*, 2006) Patient interval (Lim *et al*, 1974) Primary care interval (Lim *et al*, 1974)	**Survival** Symptom onset to diagnosis (Maconi *et al*, 2003) Patient interval (Ziliotto *et al*, 1987)
		
	**Stage** Diagnostic interval (Fernandez *et al*, 2002) Patient interval (Tokuda *et al*, 2009)	**Stage** Diagnostic interval (Haugstvedt *et al*, 1991)
**Oesophageal**		
**Stage** Symptom onset to diagnosis (Martin *et al*, 1997)	**Stage** Diagnostic interval (Fernandez *et al*, 2002) Patient interval (Tokuda *et al*, 2009)	**Stage** Symptom onset to treatment (Wang *et al*, 2008)
**Gastro-oesophageal**		
**Survival** None reported	**Survival** Referral interval (Sharpe *et al*, 2010)	None reported
		
**Other outcomes** Treatment interval and morbidity and in-hospital mortality (Grotenhuis *et al*, 2010)		
**Pancreatic**		
**Survival** Symptom onset to diagnosis (Gobbi *et al*, 2013) Symptom onset to referral (Raptis *et al*, 2010)	**Survival** Treatment interval (Yun *et al*, 2012)	None reported
		
	**Stage** Patient interval (Tokuda *et al*, 2009)	
		
	**Other outcomes** Diagnostic interval and resectability (McLean *et al*, 2013)	
**Hepatocellular**		
**Survival** Treatment interval (Singal *et al*, 2013)		None reported
		
	**Stage** Patient interval (Tokuda *et al*, 2009)	
**Colorectal**		
**Survival** Diagnostic interval (Tørring *et al*, 2011, 2012, 2013) Treatment interval (Gort *et al*, 2010–colon only; Yun *et al*, 2012–rectal only)	**Survival** Diagnostic interval (Pruitt *et al*, 2013) Referral interval (Zafar *et al*, 2011; Currie *et al*, 2012) Symptom onset to treatment (Thompson *et al*, 2011) First presentation to diagnosis (Singh *et al*, 2012) Treatment interval (Roland *et al*, 2013)	
		
**Stage** Treatment interval (Guzman-Laura *et al*, 2011) colon Referral interval (Valentin-Lopez *et al*, 2012)	**Stage** Symptom onset to treatment (Terhaar sive Droste *et al*, 2010; Cerdan-Santacruz *et al*, 2011; Deng *et al*, 2012) Referral interval (Ramsay *et al*, 2012) Treatment interval (Van Hout *et al*, 2011) Symptom onset to treatment (Van Hout *et al*, 2011) Patient interval (Cerdan-Santacruz *et al*, 2011; Van Hout *et al*, 2011)	**Stage** Treatment interval (Guzman-Laura *et al*, 2011) rectal
		
	**Other outcomes** Patient interval and satisfaction (Tomlinson *et al*, 2012)	
**Prostate**		
**Survival** Diagnostic interval (Tørring *et al*, 2013) Diagnosis to treatment (O'Brien *et al*, 2011)	**Survival** Diagnosis to treatment (Korets *et al*, 2012; Sun *et al*, 2012) Referral interval (Neal *et al*, 2007)	None reported
		
	**Stage** Diagnosis to treatment (Korets *et al*, 2012; Sun *et al*, 2012) Patient interval (Tokuda *et al*, 2009)	
**Testicular**		
**Survival** Patient interval (Hanson *et al*, 1993) Diagnostic interval (Huyghe *et al*, 2007; Moul *et al*, 1990–non-seminoma only) Symptom onset to treatment (Prout and Griffin, 1984; Medical Research Council Working Party, Testicular Tumours, 1985)	**Survival** Patient interval (Fossa *et al*, 1981) Symptom onset to treatment (Dieckmann *et al*, 1987) Symptom onset to treatment Meffan *et al*, 1991) Diagnostic interval (Moul *et al*, 1990; Harding *et al*, 1995–seminoma only; Fossa *et al*, 1981)	None reported
**Stage** Symptom onset to treatment (Ware *et al*, 1980; Wishnow *et al*, 1990) Patient interval (Ware *et al*, 1980; Chilvers *et al*, 1989) Diagnostic interval (Bosl *et al*, 1981; Moul *et al*, 1990; Huyghe *et al*, 2007–non-seminoma only) Patient interval (Hanson *et al*, 1993)	**Stage** Symptom onset to treatment (Dieckmann *et al*, 1987) Symptom onset to treatment Meffan *et al*, 1991) Diagnostic interval (Harding *et al*, 1995)	
		
**Other outcomes** Diagnostic interval and chance of complete remission (Akdas *et al*, 1986); and response to treatment (Scher *et al*, 1983)	**Other outcomes** Symptom onset to treatment and relapse rate (Napier and Rustin, 2000)	
**Renal**		
None reported	**Stage** Patient interval (Tokuda *et al*, 2009)	**Stage** Symptom onset to treatment (Holmang and Johansson, 2006)
**Bladder**		
**Survival** Symptom onset to diagnosis (Hollenbeck *et al*, 2010) Symptom onset to referral (Wallace *et al*, 2002)	**Survival** Treatment interval (Gulliford *et al*, 1991) Referral interval (Wallace *et al*, 2002) Symptom onset to treatment (Mommsen *et al*, 1983)	None reported
		
**Stage** Diagnostic interval (Liedberg *et al*, 2003)	**Stage** Symptom onset to diagnosis (Maguire *et al*, 1994) Patient interval (Tokuda *et al*, 2009)	
**Upper tract urothelial carcinoma**		
	**Survival** Diagnosis to treatment (Waldert *et al*, 2010; Sundi *et al*, 2012)	None reported
		
**Stage** Diagnosis to treatment (Waldert *et al*, 2010)		
**Cervical**		
	**Survival** Treatment interval (Umezu *et al*, 2012)	None reported
		
**Stage** Patient interval (Fruchter and Boyce, 1981)	**Stage** Primary care interval (Fruchter and Boyce, 1981) Patient interval (Tokuda *et al*, 2009)	
**Endometrial**		
	**Survival** Symptom onset to diagnosis (Menczer *et al*, 1995)	**Survival** Referral to treatment interval (Crawford *et al*, 2002) Diagnosis to treatment interval (Elit *et al*, 2013)
		
**Stage** Symptom onset to diagnosis (Fruchter and Boyce, 1981; Franceschi *et al*, 1983; Obermair *et al*, 1996)	**Stage** Symptom onset to diagnosis (Pirog *et al*, 1997) Patient interval (Tokuda *et al*, 2009)	
		
**Other outcomes** Symptom onset to treatment and quality of life and satisfaction (Robinson *et al*, 2012)		
**Ovarian**		
	**Survival** Symptom onset to diagnosis (Nagle *et al*, 2011) Referral interval (Neal *et al*, 2007)	
		
	**Stage** Patient interval (Smith and Anderson, 1985; Tokuda *et al*, 2009) Symptom onset to diagnosis (Fruchter and Boyce, 1981; Menczer *et al*, 2009; Nagle *et al*, 2011)	**Stage** Symptom onset to diagnosis (Lurie *et al*, 2010)
		
**Other outcomes** Symptom onset to treatment and quality of life and satisfaction (Robinson *et al*, 2012)		
**Head and neck**		
**Survival** Patient interval (Koivunen *et al*, 2001–pharyngeal; Teppo and Alho, 2008–pharyngeal and laryngeal cancers (separately)) Diagnostic interval (Alho *et al*, 2006–head and neck unspecified; Teppo *et al*, 2003–laryngeal; Teppo and Alho, 2008–laryngeal) Symptom onset to treatment (Hansen *et al*, 2004–laryngeal) Treatment interval (Sidler *et al*, 2010–nasopharyngeal)	**Survival** Patient interval (Teppo *et al*, 2003–laryngeal; Teppo and Alho, 2008–tongue) Diagnostic interval (Seoane *et al*, 2010–oral; Teppo and Alho, 2008–pharyngeal and tongue (separately); Koivunen *et al*, 2001–pharyngeal) Symptom onset to diagnosis (Wildt *et al*, 1995–oral) Symptom onset to treatment (McGurk *et al*, 2005–head and neck unspecified) Treatment interval (Caudell *et al*, 2011–head and neck unspecified; Brouha *et al*, 2000–laryngeal)	None reported
		
**Stage** Patient interval (Kumar *et al*, 2001–oral; Brouha *et al*, 2005b–oral and pharyngeal cancer (separately); Lee *et al*, 1997–nasopharyngeal; Sheng *et al*, 2008–nasopharyngeal; Tromp *et al*, 2005–head and neck unspecified; Tokuda *et al*, 2009–head and neck unspecified; Tromp *et al*, 2005–head and neck unspecified) Diagnostic interval (Allison *et al*, 1998–aerodigestive tract; Al-Rajhi *et al*, 2009–nasopharyngeal) Symptom onset to diagnosis (Allison *et al*, 1998–aerodigestive tract; Al-Rajhi *et al*, 2009–nasopharyngeal) Symptom onset to referral (Pitchers and Martin, 2006–oropharyngeal)	**Stage** Patient interval (Allison *et al*, 1998–upper aerodigestive tract; Al-Rajhi *et al*, 2009–nasopharyngeal; Brouha *et al*, 2005a–laryngeal cancer; Wildt *et al*, 1995–oral; Teppo *et al*, 2009–vestibular schwannoma) Diagnostic interval (Teppo *et al*, 2009–vestibular schwannoma; Ho *et al*, 2004–oropharyngeal) Symptom onset to diagnosis (Miziara *et al*, 1998–laryngeal; Scott *et al*, 2005–oral) Symptom onset to referral (Vernham and Crowther, 1994 head and neck unspecified) Symptom onset to treatment (McGurk *et al*, 2005–head and neck unspecified)	
		
**Other outcomes** Diagnostic interval and risk of recurrence (Teppo *et al*, 2005–laryngeal)	**Other outcomes** Patient interval and risk of recurrence (Teppo *et al*, 2005–laryngeal)	
**Brain/CNS**		
**Other outcomes** Symptom onset to diagnosis and progressive neurological deterioration (Balasa *et al*, 2012)	None reported	None reported
**Melanoma**		
**Survival** Patient interval (Temoshok *et al*, 1984, Montella *et al*, 2002) Diagnostic interval (Temoshok *et al*, 1984; Metzger *et al*, 1998; Montella *et al*, 2002; Tørring *et al*, 2013)		None reported
		
**Stage** Patient interval (Richards *et al*, 1999) Symptom onset to diagnosis (Helsing *et al*, 1997)	**Stage** Patient interval (Cassileth *et al*, 1982, Schmid-Wendtner *et al*, 2002; Carli *et al*, 2003; Baade *et al*, 2006) Diagnostic interval (Cassileth *et al*, 1982, Schmid-Wendtner *et al*, 2002; Baade *et al*, 2006) Symptom onset to diagnosis (Krige *et al*, 1991; Baade *et al*, 2006)	
**Non-melanoma skin**		
**Stage** Patient interval (Tokuda *et al*, 2009)		None reported
		
**Other outcomes** Symptom onset and presentation to specialist care and increase in tumour size (Alam *et al*, 2011)	**Other outcomes** Symptom onset to treatment and larger lesions (Renzi *et al*, 2010)	
**CTYA**		
**Survival** Symptom onset to diagnosis (Marwaha *et al*, 2010b–leukaemia; Ferrari *et al*, 2010–soft tissue sarcomas) First seen in specialist care to diagnosis (Marwaha *et al*, 2010a–leukaemia)	**Survival** Symptom onset to diagnosis (Kameda-Smith *et al*, 2013–soft tissue sarcomas; Sethi *et al*, 2013–posterior fossa tumours) Diagnostic interval (Lins *et al*, 2012–leukaemia; Crawford *et al*, 2009–primary spinal cord tumours) Patient interval (Yang *et al*, 2009 –osteosarcoma) Symptom onset to diagnosis (Brasme *et al*, 2012a, 2012b–medulloblastoma; Loh *et al*, 2012–paediatric solid tumours; Butros *et al*, 2002–retinoblastoma)	**Survival** Patient interval (Kukal *et al*, 2009–brain tumours) First symptom to treatment (Erwenne and Franco, 1989–retinoblastoma)
		
**Stage** Diagnostic interval (Wallach *et al*, 2006–retinoblastoma)	**Stage** Patient interval (Yang *et al*, 2009 –osteosarcoma; Simpson *et al*, 2005–Ewing's sarcoma) Symptom onset to diagnosis and eye loss (Butros *et al*, 2002–retinoblastoma)	**Stage** Diagnostic interval (Crawford *et al*, 2009–primary spinal cord tumours; Halperin *et al*, 2001–medulloblastoma; Bacci *et al*, 1999–Ewing's sarcoma)
		
**Other outcomes** Symptom onset to treatment and extra-ocular disease (Erwenne and Franco, 1989–retinoblastoma)	**Other outcomes** Patient interval and eye loss (Goddard and Kingston, 1999–retinoblastoma) Treatment interval and relapse rate (Wahl *et al*, 2012–leukaemia)	
**Leukaemia**		
None reported	**Survival** Diagnostic interval (Friese *et al*, 2011 (chronic lymphocytic)) Symptom onset to diagnosis (Prabhu *et al*, 1986 (chronic myeloid)) Treatment interval (Bertoli *et al*, 2013 (acute myeloid))	None reported
**Lymphoma**		
None reported	**Survival** Symptom onset to diagnosis (Jacobi *et al*, 2008 (follicular); Maguire *et al*, 1994 (unspecified); Norum, 1995 (Hodgkin's))	**Survival** Symptom onset to diagnosis (Kim *et al*, 1995; Foulc *et al*, 2003 (both Sezary syndrome))
**Myeloma**		
**Survival** Symptom onset to diagnosis (Kariyawasan *et al*, 2007)	None reported	None reported
		
**Other outcomes** Symptom onset to diagnosis and complications at diagnosis (Kariyawasan *et al*, 2007; Friese *et al*, 2009)		
**Connective tissue**		
**Survival** Symptom onset to treatment (Ruka *et al*, 1988 (soft tissue sarcoma)) Symptom onset to diagnosis (Saithna *et al*, 2008 (soft tissue sarcoma)) Symptom onset to diagnosis (Nakamura *et al*, 2011 (soft tissue sarcoma))	**Survival** Symptom onset to diagnosis (Rougraff *et al*, 2007 (soft tissue sarcoma); Wurtz *et al*, 1999 (osteosarcoma)	None reported
		
	**Stage** Symptom onset to diagnosis (Bacci *et al*, 2002 (osteosarcoma))	
**Carcinoid**		
None reported	**Survival** Symptom onset to diagnosis (Toth-Fejel and Pommier, 2004)	None reported
	**Stage** Symptom onset to diagnosis (Toth-Fejel and Pommier, 2004)	
**Thyroid**		
None reported	**Stage** Patient interval (Tokuda *et al*, 2009)	None reported
**Multisite**		
**Survival** Diagnostic interval (Tørring *et al*, 2013 (breast, lung, colorectal, prostate and melanoma combined)	None reported	None reported

## Box 1. Key quality criteria for studies that examine the relationship between time intervals in cancer diagnosis and outcomes.

Good studies will report the definition of intervals in compliance with definitions in the Aarhus statement (Weller *et al*, 2012). The most common intervals reported are as follows:

- Patient interval – the time from when bodily changes and/or first symptoms are noticed to presentation of this change or symptom to a health care professional
- Diagnostic interval – the date from first presentation to a health care professional to diagnosis
- Referral interval – the date from referral to specialist care to being seen in specialist care

Good studies will report key dates in the diagnostic journey in a standardised way, with a full description. This includes, for example, the following:

- Dates when patients first notices bodily changes or symptoms and when they decide to seek help
- Date of first presentation of potential cancer symptom – including how such symptoms were defined
- Date of diagnosis – clear reporting of how this date was obtained and what date it actually represents (e.g., date of tissue diagnosis, date when patient informed)
- Dates of referral / investigation – including definitions of which were included and why

Good studies will fully describe appropriate data collection methods for time intervals. These will vary by interval, and different approaches to data collection (e.g., interviews, questionnaires, medical records, database studies) will give different answers. Data collection from patients is preferred for studies measuring patient intervals. Precise details regarding data collection methods are preferred.Good studies will fully describe and justify outcome measures. Mortality or survival is preferred, but some measure of stage (or other measure of disease severity or treatment modality) is also a useful endpoint. Studies capturing patient experience and quality of life and psychological outcomes are also needed.

Good studies will use a design that addresses bias and confounding (including confounding by indication); this includes measures to address the waiting time paradox.
