# Supplementary material for: Is increased time to diagnosis and treatment in symptomatic cancer associated with poorer outcomes? Systematic review
Source: Br J Cancer. 2015 Mar 3;112(Suppl 1):S92–S107. doi: 10.1038/bjc.2015.48 (PMC4385982; doi:10.1038/bjc.2015.48)
Supplement: Supplementary Information [file bjc201548x1.docx]

**Supplementary Online Material - Bias assessment**

| **Author** | | **Sample representativeness** | **Characteristics reporting** | **Representativeness of participants** | **Bias minimisation** | **Independent variable assessment** | **A priori definition** | **Appropriate definition** | **Multi-variate analysis** | **Prognostic adjustment** | **Outlier adjustment** | **Confounder adjustment** |
| --- | --- | --- | --- | --- | --- | --- | --- | --- | --- | --- | --- | --- |
| **Breast** | | | | | | | | | | | | |
| Brazda (2010) | | Yes | Yes | Yes | No | Yes | Yes | Yes | No | No | No | No |
| Eastman (2013) | | Yes | Ethnicity | Not applicable | No | Not applicable | Yes | Yes | Yes | No | No | No |
| Ermiah (2012) | | Yes | Yes | Can't tell | Yes | No | Yes | Yes | No | No | No | No |
| McLaughlin (2012) | | NC Medicaid enrollees | Yes | Not applicable | No | Not applicable | Yes | Yes | Yes | Yes | No | No |
| Mujar (2013) | | Yes | Yes | Yes | No | Not applicable | Yes | Yes | Yes | Yes | No | Yes |
| Redaniel (2013) | | Yes | Yes | Not applicable | No | Not applicable | Yes | Yes | Yes | Yes | No | No |
| Smith (2013) | | Exclusions due to missing information.  Pregnancy status may account for delay. | Yes | Not applicable | No | Not applicable | Yes | Yes | Yes | No | No | No |
| Sue (2013) | | Only patients who were treated with definitive surgical excision | Age, ethnicity | Not applicable | No | Not reported | Yes | Yes | No | No | Not applicable | No |
| Tørring (2013) | | Yes | Age, gender | 16% excluded | Yes | Not applicable | Yes | Yes | Yes | Yes | Yes | Yes |
| Wagner (2011) | | Many patients with stage II and III tumors were treated with chemotherapy | Age | Not applicable | No | Not applicable | Yes | Yes | Yes | No | Not applicable | No |
| Warner (2012) | | Yes | Yes | Not applicable | Yes | Not applicable | Yes | Yes | Yes | Yes | No | Yes |
| Wright (2010) | | Yes | Yes | Not applicable | No | Not applicable | Yes | Yes | Yes | No | No | No |
| Yun (2012) | | Yes | Age, gender | Not applicable | Can't tell | Not applicable | Yes | Yes | Yes | Yes | No | Can’t tell |
| **Lung** | | | | | | | | | | | | |
| Annakkaya (2007) | | Yes | Age, gender | Yes | No | No | Yes | Yes | Yes | No | No | No |
| Brocken (2012) | | Can't tell | Yes | Yes | Can't tell | Not reported | Yes | Yes | Yes | Can't tell | No | No |
| Christensen (1997) | | Yes | Age, gender | Yes | No | No | Yes | Yes | No | No | No | No |
| Diaconescu (2011) | | Can't tell | Yes | Can't tell | No | Not reported | Yes | Yes | Yes | Yes | Can't tell | Can't tell |
| Gonzalez-Barcala (2010) | | Can't tell | Yes | Not applicable | No | Not reported | Yes | Yes | Yes | Yes | Can't tell | No |
| Gould (2008) | | Yes | Age, gender, race | Can’t tell | No | No | Yes | Yes | No | No | No | No |
| Loh (2006) | | Yes | Yes | Yes | No | No | Yes | Yes | No | No | No | No |
| Maguire (1994) | | Yes | Age, gender | Can’t tell | No | No | Yes | Yes | No | No | No | No |
| Mohan (2006) | | Yes | Yes | Yes | Yes | No | Yes | Yes | No | No | No | No |
| Murai (2012) | | Yes | Age, gender, stage | Not applicable | No | Yes | Yes | Yes | No | No | No | No |
| Myrdal (2004) | | Yes | Age, gender | Can’t tell | No | No | Yes | Yes | Yes | No | No | Yes |
| Neal (2007) | | Yes | Data collected but not reported | Can’t tell | No | No | No | Yes | Yes | No | No | No |
| Pita Fernandez (2003) | | Can’t tell | Ager, gender | Can’t tell | No | No | Yes | Yes | No | No | No | No |
| Radzikowska (2012) | | Yes | Age, performance status | Not applicable | Can't tell | Not reported | Yes | Yes | Yes | Yes | No | No |
| Salomaa (2005) | | Yes | Yes | Can’t tell | No | No | Yes | Yes | Yes | No | No | No |
| Skaug (2011) | | Yes | Yes | Yes | Yes | Not reported | Yes | Yes | Yes | Yes | No | No |
| Tokuda (2009) | | Yes | Age, gender | Not applicable | No | No | Yes | Yes | Yes | No | No | No |
| Tørring (2013) | | Yes | Age, gender | 16% excluded | Yes | Not applicable | Yes | Yes | Yes | Yes | Yes | Yes |
| Yilmaz (2008) | | Yes | Yes | Yes | No | No | Yes | Yes | Yes | No | No | No |
| Yun (2012) | | Yes | Age, gender | Not applicable | Can't tell | Not applicable | Yes | Yes | Yes | Yes | No | Can’t tell |
| **Gastro intestinal tract** | | | | | | | | | | | | |
| **Gastric** | | | | | | | | | | | | |
| Arvanitakis (1992) | | No | Yes | Can’t tell | No | No | Yes | Yes | No | No | No | No |
| Fernandez (2002) | | Yes | Yes | Can’t tell | Yes | No | Yes | Yes | No | No | No | No |
| Haugstvedt (1991) | | Can’t tell | Yes | Can’t tell | No | No | Yes | Yes | No | Yes | No | Yes |
| Lim (1974)  Maconi (2003)  Maguire (1994) | | Yes | Sex, race | Can’t tell | No | No | Yes | Yes | No | No | No | No |
| Maconi (2003) | | No | Age, gender | Can’t tell | No | No | Yes | Yes | No | Yes | No | Yes |
| Maguire (1994) | | Yes | Age, gender | Can’t tell | No | No | Yes | Yes | No | No | No | No |
| Martin (1997) | | Yes | Age, gender | Can’t tell | No | No | Yes | Yes | No | No | No | No |
| Tokuda (2009) | | Yes | Age, gender | Not applicable | No | No | Yes | Yes | Yes | No | No | No |
| Windham (2002) | | Yes | Yes | Can’t tell | No | No | Yes | Yes | No | No | No | No |
| Yun (2012) | | Yes | Age, gender | Not applicable | Can't tell | Not applicable | Yes | Yes | Yes | Yes | No | Can’t tell |
| Zilliotto (1987) | | Yes | Age, gender | Can’t tell | No | No | Yes | Yes | No | No | No | No |
| **Oesophageal** | | | | | | | | | | | | |
| Fernandez (2002) | | Yes | Yes | Can’t tell | Yes | No | Yes | Yes | No | No | No | No |
| Martin (1997) | | Yes | Age, gender | Can’t tell | No | No | Yes | Yes | No | No | No | No |
| Tokuda (2009) | | Yes | Age, gender | Not applicable | No | No | Yes | Yes | Yes | No | No | No |
| Wang (2008) | | Yes | Age, gender | Can’t tell | No | No | Yes | Yes | Yes | No | No | No |
| **Gastric and oesophageal** | | | | | | | | | | | | |
| Grotenhuis (2010) | | Not reported | Age, gender | Yes | No | Not applicable | Yes | Yes | No | No | No | No |
| Sharpe (2010) | | Can’t tell | Age, gender | Not applicable | No | Not applicable | Yes | Yes | No | No | Not applicable | No |
| **Pancreatic** | | | | | | | | | | | | |
| Gobbi (2013) | | Not reported | Yes | Not reported | No | Not applicable | Yes | No | Yes | Can't tell | No | No |
| McLean (2013) | | Can't tell | Not reported | Yes | No | Not reported | Yes | Yes | Yes | No | No | No |
| Raptis (2010) | | Yes | Age | Yes | Can't tell | Not reported | Yes | Yes | Yes | Can't tell | Not applicable | No |
| Tokuda (2009) | | Yes | Age, gender | Not applicable | No | No | Yes | Yes | Yes | No | No | No |
| Yun (2012) | | Yes | Age, gender | Not applicable | Can't tell | Not applicable | Yes | Yes | Yes | Yes | No | Can’t tell |
| **Hepatocellular** | | | | | | | | | | | | |
| Singal (2013) | | Yes | Yes | Yes | Yes | No | Yes | Yes | Yes | Yes | No | No |
| Tokuda (2009) | | Yes | Age, gender | Not applicable | No | No | Yes | Yes | Yes | No | No | No |
| **Colorectal** | | | | | | | | | | | | |
| Cerdan-Santacruz (2011) | | Yes | Yes | Yes | Can't tell | Not reported | Yes | Yes | No | No | No | No |
| Currie (2011) | | Can’t tell | Age, gender | Not applicable | Can't tell | Not applicable | Yes | Yes | Yes | No | No | No |
| Deng (2012) | | Han Chinese recruited in one hospital | Yes | Yes | Yes | Yes | Yes | Yes | Yes | No | No | Yes |
| Gort (2010) | | Yes | Yes | Yes | Yes | Not applicable | Yes | Yes | Yes | No | No | No |
| Guzman (2011) | | Yes | Yes | Yes | Can't tell | Not applicable | Yes | Yes | Yes | No | No | Yes |
| Pruitt (2013) | | Older Medicare patients | Yes | Yes | Yes | Not applicable | Yes | Yes | Yes | Yes | Yes | No |
| Ramsay (2012) | | Yes | Yes | Yes | No | Not applicable | Yes | Yes | No | No | No | No |
| Roland (2013) | | Yes | Yes | Yes | Can't tell | Not applicable | Yes | Yes | Yes | No | No | No |
| Singh (2012) | | Yes | Age, gender | Yes | Can't tell | Not applicable | Yes | Yes | Yes | No | No | Yes |
| Terhaar sive Droste (2010) | | Yes | Yes | Yes | No | No | Yes | Yes | Yes | Yes | No | No |
| Thompson (2011) | | Yes | Yes | Clinical details of 169 patients not analysed suggest worse outcome | Yes | Not applicable | Yes | Yes | Yes | Yes | No | Yes |
| Tomlinson (2012) | | Can't tell | Yes | Can't tell | Yes | Not reported | Yes | Yes | Yes | No | No | No |
| Tørring (2011) | | Yes | Yes | Yes | Yes | Not applicable | Yes | Yes | Yes | Yes | Yes | Yes |
| Tørring (2012) | | Yes | Yes | Yes | Yes | Not applicable | Yes | Yes | Yes | Yes | Yes | Yes |
| Tørring (2013) | | Yes | Age, gender | 16% excluded | Yes | Not applicable | Yes | Yes | Yes | Yes | Yes | Yes |
| Valentin-Lopez (2012) | | Yes | Yes | Can't tell | No | Not reported | Yes | Yes | Yes | No | No | No |
| Van Hout (2011) | | Yes | Yes | Yes | Yes | Not applicable | Yes | Yes | Yes | No | No | Yes |
| Yun (2012) | | Yes | Age, gender | Not applicable | Can't tell | Not applicable | Yes | Yes | Yes | Yes | No | Can’t tell |
| Zafar (2012) | | Yes | Yes | Yes | Yes | Not applicable | Yes | Yes | Yes | Can't tell | No | No |
| **Renal tract** | | | | | | | | | | | | |
| **Prostate** | | | | | | | | | | | | |
| Korets (2012) | | Not reported | Yes | Not applicable | No | Not applicable | Yes | Yes | Yes | No | No | No |
| Neal (2007) | | Yes | Data collected but not reported | Can’t tell | No | No | No | Yes | Yes | No | No | No |
| O'Brien (2011) | | Not reported | Only age clearly reported | Not applicable | No | Not applicable | Yes | Yes | Yes | No | No | No |
| Sun (2012) | | Yes | Yes | Yes | No | Not applicable | Yes | Yes | Yes | Yes | No | No |
| Tokuda (2009) | | Yes | Age, gender | Not applicable | No | No | Yes | Yes | Yes | No | No | No |
| Tørring (2013) | | Yes | Age, gender | 16% excluded | Yes | Not applicable | Yes | Yes | Yes | Yes | Yes | Yes |
| **Renal** | | | | | | | | | | | | |
| Holmang (2006) | | No | Age, gender | Can’t tell | Yes | Yes | Yes | Yes | Yes | Yes | No | Yes |
| Tokuda (2009) | | Yes | Age, gender | Not applicable | No | No | Yes | Yes | Yes | No | No | No |
| **Bladder** | | | | | | | | | | | | |
| Gulliford (1991) | | No | Age, gender | Not applicable | No | No | Yes | Yes | Yes | Yes | No | Yes |
| Hollenbeck (2010) | | Yes | Yes | Not applicable | Can't tell | Not applicable | Yes | Yes | Yes | Yes | No | No |
| Liedberg (2003) | | Yes | Age, gender | Can’t tell | No | No | Yes | Yes | No | No | No | No |
| Maguire (1994) | | Yes | Age, gender | Can’t tell | No | No | Yes | Yes | No | No | No | No |
| Mommsen (1983) | | Yes | Age, gender | Can’t tell | Yes | Yes | Yes | Yes | No | No | No | No |
| Tokuda (2009) | | Yes | Age, gender | Not applicable | No | No | Yes | Yes | Yes | No | No | No |
| Wallace (2002) | | Yes | Yes | Can’t tell | No | No | Yes | Yes | Yes | Yes | No | Yes |
| **Testicular** | | | | | | | | | | | | |
| Adkas (1986) | | Can’t tell | Not reported | Can’t tell | Yes | No | Yes | Yes | No | No | No | No |
| Bosl (1981) | | Yes | Age | Can’t tell | No | No | Yes | Yes | No | No | No | No |
| Chilvers (1989) | | Can’t tell | Not reported | Can’t tell | No | No | Yes | Yes | Yes | Yes | No | Yes |
| Dieckmann (1987) | | Yes | Data collected but not reported | Can’t tell | No | No | Yes | Yes | No | No | No | No |
| Fossa (1981) | | Yes | Age | Can’t tell | No | No | Yes | Yes | No | No | No | No |
| Hanson (1993) | | Yes | Age | Can’t tell | No | No | Yes | Yes | No | No | No | No |
| Harding (1995) | | Yes | Not reported | Can’t tell | No | No | Yes | Yes | No | No | No | No |
| Huyghe (2007) | | Yes | Not reported | Can’t tell | No | No | Yes | Yes | No | No | No | No |
| Meffan (1991) | | Yes | Not reported | Can’t tell | No | No | Yes | Yes | No | No | No | No |
| Moul  (1990) | | Yes | Yes | Can’t tell | No | No | Yes | Yes | No | No | No | No |
| MRC Working Party (1985) | | Yes | Not reported | Can’t tell | No | No | Yes | Yes | Yes | Yes | No | No |
| Napier (2000) | | Yes | Age | Can’t tell | No | No | Yes | Yes | No | No | No | No |
| Prout (1984) | | Yes | Age | Can’t tell | No | No | Yes | Yes | No | No | No | No |
| Scher (1983) | | Can’t tell | Not reported | Can’t tell | No | No | Yes | Yes | No | No | No | No |
| Ware (1980) | | Can’t tell | Not reported | Can’t tell | No | No | Yes | Yes | No | No | No | No |
| Wishnow (1990) | | Yes | Not reported | Can’t tell | No | No | Yes | Yes | No | No | No | No |
| **Upper Tract Urothelial** | | | | | | | | | | | | |
| Sundi (2012) | | Selection bias | Yes | Not applicable | No | Not reported | Yes | Yes | Yes | No | Yes | Yes |
| Waldert (2010) | | Not reported | Age, gender | Not applicable | No | Not applicable | Yes | Yes | Yes | No | No | Can't tell |
| **Gynaecological** | | | | | | | | | | | | |
| **Cervical** | | | | | | | | | | | | |
| Fruchter (1981) | | Yes | Not reported | Can’t tell | Yes | Yes | Yes | Yes | No | No | No | No |
| Tokuda (2009) | | Yes | Age, gender | Not applicable | No | No | Yes | Yes | Yes | No | No | No |
| Umezu (2012) | | Not reported | Age | Not applicable | No | Not reported | Yes | Yes | Yes | No | No | No |
| **Endometrial** | | | | | | | | | | | | |
| Crawford (2002) | | Yes | Age | Not applicable | No | No | Yes | Yes | Yes | Yes | No | Yes |
| Elit (2013) | | Can't tell | Yes | Yes | No | No | Yes | Yes | Yes | Yes | No | No |
| Franceschi (1983) | | Yes | Yes | Yes | Yes | Yes | Yes | Yes | No | Yes | No | Yes |
| Fruchter (1981) | | Yes | Not reported | Can’t tell | Yes | Yes | Yes | Yes | No | No | No | No |
| Menczer (1995) | | Yes | Yes | Not applicable | Yes | No | Yes | Yes | Yes | Yes | No | Yes |
| Obermair (1996) | | Can’t tell | Age | Can’t tell | Can’t tell | No | Yes | Yes | No | No | No | No |
| Pirog (1997) | | Yes | Yes | Not applicable | No | No | Yes | Yes | No | No | No | No |
| Robinson (2012) | | Yes | Age | Non-participants younger and older than participants | No | No | Yes | Yes | Yes | Yes | No | No |
| Tokuda (2009) | | Yes | Age, gender | Not applicable | No | No | Yes | Yes | Yes | No | No | No |
| **Ovarian** | | | | | | | | | | | | |
| Fruchter (1981) | | Yes | Not reported | Can’t tell | Yes | Yes | Yes | Yes | No | No | No | No |
| Lurie (2010) | | Yes | Yes | Not applicable | Yes | Not reported | Yes | Yes | Yes | No | No | No |
| Menzcer (2009) | | Selection bias | Age | Yes | Yes | Not reported | Yes | Yes | No | No | No | No |
| Nagle (2011) | | Yes | Yes | Yes | Yes | Yes | Yes | Yes | Yes | No | No | No |
| Neal (2007) | | Yes | Data collected but not reported | Can’t tell | No | No | No | Yes | Yes | No | No | No |
| Robinson (2012) | | Yes | Age | Non-participants younger and older than participants | No | No | Yes | Yes | Yes | Yes | No | No |
| Smith (1985) | | Yes | Data collected but not reported | Yes | Yes | No | Yes | Yes | No | No | No | No |
| Tokuda (2009) | | Yes | Age, gender | Not applicable | No | No | Yes | Yes | Yes | No | No | No |
| **Head and Neck** | | | | | | | | | | | | |
| Alho (2006) | | Yes | Age, gender | Not applicable | Yes | No | Yes | Yes | Yes | No | No | No |
| Allison (1998) | | Yes | Yes | Yes | Yes | No | Yes | Yes | Yes | Yes | No | Yes |
| Al-Rajhi (2009) | | Yes | Yes | Yes | No | No | Yes | Yes | Yes | Yes | No | Yes |
| Brouha (2000) | | Yes | Yes | Not applicable | No | No | Yes | Yes | Yes | Yes | No | No |
| Brouha  (2005a) | | Can’t tell | Yes | Yes | Yes | No | Yes | Yes | No | No | No | No |
| Brouha  (2005b) | | Can’t tell | Yes | Yes | Yes | No | Yes | Yes | No | No | No | No |
| Caudell (2011) | | Not reported | Yes | Yes | No | Not applicable | Yes | Yes | Yes | No | No | No |
| Hansen (2005) | | Yes | Age, gender | Not applicable | No | No | Yes | Yes | Yes | No | No | No |
| Ho (2004) | | Yes | Yes | Not applicable | No | No | Yes | Yes | Yes | Yes | No | Yes |
| Koivunen (2001) | | Yes | Yes | Not applicable | No | No | Yes | Yes | No | No | No | No |
| Kumar (2001) | | Yes | Yes | Yes | No | No | Yes | Yes | No | No | No | No |
| Lee (1997) | | Yes | Age, gender | Yes | No | No | Yes | Yes | Yes | Yes | No | Yes |
| McGurk (2005) | | Yes | Demographic data collected but not reported | Can’t tell | No | No | Yes | Yes | No | No | No | No |
| Miziara (1998) | | Yes | Not reported | Yes | No | No | Yes | Yes | No | No | No | No |
| Pitchers (2006) | | Yes | Age, gender | Not applicable | Yes | No | Yes | Yes | No | No | No | No |
| Scott (2005) | | Yes | Yes | Not applicable | No | No | Yes | Yes | Yes | Yes | No | Yes |
| Seoane (2010) | | Not reported | Yes | Not reported | Yes | Not reported | Yes | No | Yes | No | Not applicable | Yes |
| Sheng (2008) | | Can’t tell | Yes | Yes | No | No | Yes | Yes | No | No | No | No |
| Sidler (2010) | | Yes | Yes | Yes | No | No | Yes | No | Yes | No | No | No |
| Teppo (2003) | | Yes | Yes | Not applicable | Yes | No | Yes | Yes | Yes | Yes | No | No |
| Teppo (2005) | | Yes | Age, gender | Not applicable | Yes | No | Yes | Yes | Yes | Yes | No | Yes |
| Teppo (2008) | | Yes | Age, gender | Not applicable | Yes | No | Yes | Yes | Yes | Yes | No | Yes |
| Teppo (2009) | | Yes | Age, gender | Yes | No | No | Yes | Yes | Yes | Yes | No | Yes |
| Tokuda (2009) | | Yes | Age, gender | Not applicable | No | No | Yes | Yes | Yes | No | No | No |
| Tromp (2005) | | Yes | Yes | Yes | Yes | No | Yes | Yes | Yes | Yes | No | Yes |
| Vernham (1994) | | Yes | Age, gender | Can’t tell | No | No | Yes | Yes | No | No | No | No |
| Wildt (1995) | | Yes | Age, gender | Not applicable | No | Yes | Yes | Yes | No | No | No | No |
| **Brain/CNS** | | | | | | | | | | | | |
| Balasa (2012) | | Not reported | Age, gender | Not reported | No | Not reported | Yes | No | No | No | No | No |
| **Melanoma** | | | | | | | | | | | | |
| Baade (2006) | | Yes | Yes | Yes | Yes | Yes | Yes | Yes | Yes | Yes | Yes | Yes |
| Carli (2003) | | Yes | Yes | Yes | Yes | Yes | Yes | Yes | Yes | Yes | Yes | Yes |
| Cassileth (1982) | | Can’t tell | Gender, occupation | Can’t tell | No | Yes | Yes | Yes | No | No | No | No |
| Helsing (1997) | | Yes | Age, gender | Yes | No | Yes | Yes | Yes | No | No | No | No |
| Krige (1991) | | Yes | Age, gender | Yes | Yes | No | Yes | Yes | No | No | No | No |
| Metzger (1998) | | Can’t tell | Age, gender | Can’t tell | No | No | Yes | Yes | No | No | No | No |
| Montella (2002) | | Yes | Yes | Yes | Yes | No | Yes | Yes | Yes | Yes | No | Yes |
| Richard (1999) | | Yes | Age, gender | Yes | Yes | Yes | Yes | Yes | No | No | Yes | No |
| Schmid-Wendtner (2002) | | Yes | Age, gender | Yes | Yes | No | Yes | Yes | No | No | No | No |
| Temoshok (1984) | | Yes | Age, gender | Yes | No | Yes | Yes | Yes | No | No | No | No |
| Tørring (2013) | | Yes | Age, gender | 16% excluded | Can't tell | Not applicable | Yes | Yes | No | Yes | Yes | Yes |
| **Non-Melanoma Skin** | | | | | | | | | | | | |
| Alam (2011) | | Reflects an affluent sub population | Yes | Yes | No | No | Yes | Yes | No | No | No | No |
| Renzi (2010) | | Yes*.* | Yes | Not applicable | Yes | Not reported | Yes | Yes | Yes | No | No | No |
| Tokuda (2009) | | Yes | Age, gender | Not applicable | No | No | Yes | Yes | Yes | No | No | No |
| **CTYA** | | | | | | | | | | | | |
| **Head and Neck** | | | | | | | | | | | | |
| Butros (2002) | | Yes | Age | No | No | No | Yes | Yes | No | No | No | No |
| Erwenne (1989) | | Yes | Age, gender | No | Yes | No | Yes | Yes | No | Yes | No | Yes |
| Goddard (1999) | | Yes | Age | Yes | Yes | No | Yes | Yes | No | No | No | No |
| Wallach (2006) | | Yes | Age, gender | No | No | No | Yes | Yes | Yes | No | No | No |
| **Brain/CNS** | | | | | | | | | | | | |
| Brasme (2012) | | Yes | Age, gender | Yes | No | Not applicable | Yes | Yes | Yes | Yes | Not applicable | Yes |
| Crawford (2009) | | Yes | Age, gender | Can’t tell | No | Yes | Yes | Yes | No | No | No | No |
| Halperin (2001) | | Yes | Age, gender, race | Not applicable | No | No | Yes | Yes | No | No | No | No |
| Kameda-Smith  (2013) | | Yes | Age, gender | Yes | Yes | No | Yes | No | No | No | No | No |
| Kukal (2009) | | Yes | Age, gender | Can’t tell | No | Yes | Yes | Yes | No | No | No | No |
| Sethi (2013) | | Yes | Age, gender | Yes | Yes | Not reported | Yes | Yes | Yes | No | No | No |
| **Leukaemia** | | | | | | | | | | | | |
| Lins (2012) | | Can't tell | Yes | Yes | No | Not reported | Yes | Yes | Yes | No | Not applicable | No |
| Marwaha (2010a) | | Yes | Age, gender | Yes | No | Not reported | Yes | Yes | Yes | No | No | Yes Yes |
| Marwaha (2010b) | | Yes | Age, gender | Yes | No | Not reported | Yes | Yes | Yes | No | No | No |
| Wahl (2012) | | Yes | Age, gender | Not applicable | No | Not reported | Yes | Yes | Yes | No | No | Yes |
| **Connective Tissue** | | | | | | | | | | | | |
| Bacci (1999) | | Yes | No data reported | Yes | No | No | Yes | Yes | No | No | No | No |
| Ferrari (2010) | Yes | Age, gender | Yes | No | No | Yes | Yes | Yes | Yes | No | No |  |
| Simpson (2005) | Yes | Age, gender | Not applicable | No | No | Yes | Yes | No | No | No | No |  |
| Yang (2009) | Yes | Age, gender | Can’t tell | No | No | Yes | Yes | No | No | No | No |  |
| **Solid Tumours** | | | | | | | | | | | |  |
| Loh (2012) | Yes | Demographics collected but not reported | Yes | No | Not applicable | Yes (EFS) No (Stage) | Yes | Yes | Can't tell | No | No |  |
| **Leukaemia** | | | | | | | | | | | |  |
| Bertoli (2013) | Yes | Yes | Not applicable | No | Yes | Yes | Yes | Yes | Yes | No | No |  |
| Friese (2011) | Yes | Yes | Not applicable | No | Not applicable | Yes | Yes | Yes | Yes | No | No |  |
| Prabhu (1986) | Not reported | Age, gender | Can’t tell | No | No | Yes | Yes | No | No | No | No |  |
| **Lymphoma** | | | | | | | | | | | |  |
| Foulc (2003) | Not reported | Age, gender | Can’t tell | No | No | Yes | Yes | Yes | Yes | No | Yes |  |
| Jacobi (2008) | Yes | Age, gender | Not applicable | No | No | Yes | Yes | Yes | No | No | No |  |
| Kim (1995) | Yes | Yes | Can’t tell | No | No | Yes | Yes | Yes | Yes | No | Yes |  |
| Maguire (1994) | Yes | Age, gender | Can’t tell | No | No | Yes | Yes | Yes | Yes | Yes | Yes |  |
| Norum (1995) | Yes | Age, gender | Can’t tell | No | No | Yes | Yes | No | No | No | No |  |
| **Myeloma** | | | | | | | | | | | |  |
| Friese (2009) | Yes | Yes | Not applicable | No | No | Yes | Yes | No | No | No | No |  |
| Kariyawasan (2007) | Yes | Age, gender | Can’t tell | No | No | Yes | Yes | No | No | No | No |  |
| **Connective Tissue** | | | | | | | | | | | |  |
| Bacci (2002) | Yes | Age, gender | Can’t tell | No | No | Yes | Yes | Yes | Yes | No | Yes |  |
| Nakamura (2011) | Only 100 sampled | Age, gender | Not applicable | No | Yes | Yes | Yes | Yes | Yes | No | No |  |
| Rougraff (2007) | Yes | Age | Can’t tell | No | No | Yes | Yes | Yes | Yes | No | Yes |  |
| Ruka (1988) | Yes | Age, gender | Can’t tell | No | No | Yes | Yes | No | Yes | No | Yes |  |
| Saiithna (2008) | Yes | Age, gender | Yes | Yes | No | Yes | Yes | Yes | No | No | Yes |  |
| Wurtz (1999) | Yes | Age, gender | Can’t tell | No | No | Yes | Yes | Yes | Yes | No | Yes |  |
| **Carcinoid** | | | | | | | | | | | |  |
| Toth-Fejel (2004) | Yes | Age, gender | Can’t tell | No | No | Yes | Yes | No | No | No | No |  |
| **Thyroid** | | | | | | | | | | | |  |
| Tokuda (2009) | Yes | Age, gender | Not applicable | No | No | Yes | Yes | Yes | No | No | No |  |
| **Multi-Site** | | | | | | | | | | | |  |
| Tørring (2013) | Yes | Age, gender | 16% excluded | Yes | Not applicable | Yes | Yes | Yes | Yes | Yes | Yes |  |
