# Supplementary material for: Is increased time to diagnosis and treatment in symptomatic cancer associated with poorer outcomes? Systematic review
Source: Br J Cancer. 2015 Mar 3;112(Suppl 1):S92–S107. doi: 10.1038/bjc.2015.48 (PMC4385982; doi:10.1038/bjc.2015.48)
Supplement: Supplementary Information [file bjc201548x2.docx]

**Supplementary Online Material - Study Characteristics**

| **Study Identifier** | | **Study Design** | **Study Aim** | **Location** | **Setting** | **Study Population** | **Participants sampled** | **Participants recruited or records available** | **Participants Analysed** | **Definition of time duration** | **Data collection method** | **Outcome measure** | **Data collection method** |
| --- | --- | --- | --- | --- | --- | --- | --- | --- | --- | --- | --- | --- | --- |
| **Breast** | | | | | | | | | | | | | |
| Brazda (2010) | | Retrospective review of patient records Cancer registry | The purpose of this study was to evaluate whether a delay from diagnosis to initial treatment in breast cancer impacts survival when equivalent clinical care protocols are provided. | USA | Specialist care (multi-site) | A retrospective review of patients undergoing breast cancer treatment between August 2005 and December 2008 in a comprehensive, multidisciplinary breast oncology program was undertaken. | Not reported | 1337 patients:  634 in CH  703 in UH | 1337 patients:  634 in CH  703 in UH | T15 | Cancer registry | Survival | Registry data (Tumour registries from two institutions) |
| Eastman (2013) | | Retrospective review of patient records | To evaluate whether delays from diagnosis to initial treatment in patients with triple negative breast cancer (TNBC) impact survival or locoregional recurrence (LRR). | USA | Specialist care (multi-site) | Patients diagnosed with TNBC between January 2004 and January 2011. Patients who received treatment elsewhere or for whom no vital status information was available were excluded. | Not reported. | 301:  County Hospital: 220;  University Hospital: 81 | 301:  County Hospital: 220; University Hospital: 81 | T15 | Patient records | Survival Locoregional recurrence | Medical records Tumour registries from hospital systems. |
| Ermiah (2012) | | Retrospective review of patient records  Patient interview | To study the diagnosis delay and its impact on stage of disease among women with breast cancer on Libya. | Libya. | Specialist care (single site) | Female patients with breast cancer diagnosed at the African (presently (2012) National) Oncology Institute (NOI), Sabratha, during the period from Jan 1, 2008 to Dec 31, 2009. | 419 | 419 | 200 | T4 | Patient records Patient interview | TNM Stage | Medical records Patient questionnaire |
| McLaughlin (2012) | | Cancer registry | To determine the impact of longer periods between biopsy-confirmed breast cancer diagnosis and the initiation of treatment on survival. | USA | North Carolina Central Cancer Registry-Medicaid Claims database. | Adult female North Carolina Medicaid enrollees diagnosed with breast cancer from January 1, 2000, through December, 31, 2002. | 1959 | 1786 | 1786 | T15 | Cancer registry | Survival Overall and breast cancer-specific survival | Registry data (Linked NC Central Cancer Registry (CCR) Medicaid Claims database). |
| Mujar (2013) | | Retrospective review of patient records | To evaluate whether time from diagnosis to primary treatment in breast cancer impacts overall survival. | Malaysia | Specialist care (single site) | Those treated in University Malaya Medical Centre (UMMC) between 1st January 2004 and 31st December 2005. | Not reported. | 648 | 648 | T15 | Patient records | Survival | Medical records Death records |
| Redaniel (2013) | | Cancer registry (West Midlands Cancer Intelligence Unit) | To assess the association between waiting time from diagnosis to first curative surgery and survival (and variations in survival between sociodemographic groups). | UK  (England) | National databases for England | All female breast cancer patients who were identified in the cancer registry, diagnosed between 1 January 1996 and 31 December 2009, who were 15 years old or more at the time of diagnosis and who had surgical resection with curative intent. | 227712 | 53689 | 53689 | T15 | Cancer registry West Midlands Cancer Intelligence Unit,  Hospital Episode Statistics and Office of National Statistics. | Survival | Registry data West Midlands Cancer Intelligence Unit  Death records Hospital Episode Statistics and Office of National Statistics. |
| Smith (2013) | | Cancer registry | To examine the impact of treatment delay time (TDT), race/ethnicity, socioeconomic status (SES), insurance status, cancer stage, and age on the survival after breast cancer diagnosis among adolescents and young adult (AYA) women. | USA | Specialist care (multi-site) | Incident breast cancer cases in AYA women diagnosed from 1997 to 2006. | 12189 | 8860 | 8860 | T15 | Cancer registry | Survival | Registry data (The California Cancer Registry) |
| Sue (2013) | | Retrospective review of patient records | To determine the factors influencing time from ductal carcinoma in-situ (DCIS) diagnosis to definitive treatment and the implications of this on overall outcome. | USA | Specialist care (single site) | Female patients presenting to the Yale Breast Centre from 2000 through 2003 diagnosed with DCIS who were subsequently treated with definitive surgical excision. | Not reported. | 127 | 127 | T15 | Patient records | Survival | Medical records |
| Tørring (2013) | | Prospective cohort study | To assess the association between the length of the diagnostic interval and the ﬁve-year mortality for the ﬁve most common cancers in Denmark while addressing the above methodological and analytical issues. | Denmark | Population based | All patients with newly diagnosed colorectal, lung, melanoma skin, breast or prostate cancer above the age of 17 in the former Danish County of Aarhus during 1 year (inclusion period from 1 September 2004 to 31 August 2005), which was equivalent to 56% of all new cancers in Denmark during that year. | 1543 | 1295 | 1128: Breast=295 (26%) | T8 | Cancer registry  GP Questionnaire | Survival | Registry data Danish Cancer Registry, County Hospital Discharge Registry Danish Civil Registration System. GP Questionnaire |
| Wagner (2011) | | Retrospective review of patient records | To evaluate the effect of time to surgery on tumour growth by comparing initial imaging and pathologic tumour size estimates. | USA | Specialist care (single site) | Patients who had been diagnosed with invasive breast carcinoma from September 2003 to December 2006. | Not reported | 818 | 818 | T15 | Patient records | Tumour size; lymph node status at surgery | Medical records |
| Warner (2012) | | Cancer registry The National Comprehensive Cancer Network (NCCN) Breast Cancer Outcomes Database | To examine the relationship between time to diagnosis and race/ethnicity, and associations between these factors and stage of disease in a multi-ethnic population in the United States. | USA | Specialist care (multi-site) | Women with new stage I-IV breast cancer diagnoses who presented and received primary care at one of eight comprehensive cancer centres between January 1, 2000 and December 31, 2007. | 25131 | 21427 | 21427 | T4 | Cancer registry  Patient intake survey | Stage  AJCC Staging  I-IV | Registry data |
| Wright (2010) | | Cancer registry California Cancer Registry database. | To evaluate demographic factors that are associated with 30 day and 90 day benchmarks for time from diagnosis to definitive treatment of breast cancer. | USA | Specialist care (multi-site) | Women with stage I to III breast cancer treated by primary surgical therapy in California hospitals between 2004 and 2007 with records in the California Cancer Registry database by April 1, 2009 were included for study. | Not reported | 19896 | 19896 | T15 | Cancer registry California Cancer Registry | Stage T Stage 1-4 | Registry data |
| Yun (2012) | | Retrospective review of patient records | To investigate the influence of hospital volume, delay of surgery, and both together on the long term survival of postoperative cancer patients. | South Korea | Population based | Patients aged >20 years who had been diagnosed with cancer of the stomach, colon, rectum, pancreas, lung or breast. | 497,339 | 266,328 | 147,682 (this number for all cancers) | T15 | Cancer registry  Health Insurance Review and Assessment Service | Survival | Registry data (Korea Central Cancer Registry), Korea National Statistical Office Database |
| **Lung** | | | | | | | | | | | | | |
| Annakkaya (2007) | | Retrospective review of medical records Consecutive patient survey | To evaluate the impact of the delayed diagnosis of lung cancer on tumour stage and patient survival and to compare the results with those of previous studies | Turkey | Specialist care | All patients admitted to the service for a suspected diagnosis of lung cancer between 1 January 2002 and 30 June 2005 | 136 | 103 | 103 | T1, T5, T8,T15 | Patient records  Patient questionnaire | TNM Stage  Survival | Medical records |
| Brocken (2012) | | Retrospective review of patient records | To evaluate the impact of symptomatology and referral type on different types of delay, to establish whether delays were related outcome and stage, and to compare the delays with those described in literature and guideline recommendations | The Netherlands | Specialist care (single site) | All consecutive patients referred to the RODP between August 1999 and April 2009. In this period, all outpatients with a radiological suspicion of lung cancer without clinical need for hospitalisation or obvious stage IV disease were diagnosed in this program in this centre. | 570 | 565 | 552 | T1, T6, T10, T13, T15 | Patient records | Stage International staging system version 6 Survival | Medical records |
| Christensen (1997) | | Retrospective review of patient records | To study the correlation between diagnostic delay and the stage of the lung cancer at the time of operation. | Denmark | Specialist care (single site) | Patients consecutively admitted for surgery between 1 January 1994 and 1 June 1995 | 172 | 172 | 172 | T5, T9, T12 | Patient records | TNM Stage | Medical records |
| Diaconescu (2011) | | Retrospective review of patient records | To evaluate prognostic factors including treatment delays in non-small cell lung cancer | Canada | Specialist care (single site) | Patients with a diagnosis of primary lung cancer between Jan 2005 and May 2007. | 665 | 605 | 495 | T8 | Cancer registry | Survival | Registry data (Local tumour registry) |
| Gonzalez-Barcala (2010) | | Retrospective review of patient records | To evaluate the delays in the management of lung cancer, both due to the delay in the patient consulting and those of the diagnostic and therapeutic process, and their relationships with patient survival. | Spain | Population based | All patients with a cytohistological confirmation of lung cancer in a 3 year period (Jan 1997 to Dec 1999) identified from information provided by the Admission and Clinical Records Department of the hospital and living in the Santiago de Compostela health area were included. | 481 | 481 | 415 | T3, T5, T14 | Patient records | Survival | Medical records |
| Gould (2008) | | Retrospective review of patient records. | To describe the variation in the timeliness of care in a sample of veterans with lung cancer, to identify predictors of timely care, and to examine the effect of more timely care on survival. | USA | Specialist care | Consecutive patients in whom non-small cell lung cancer had been diagnosed between January 1, 2002 and December 31, 2003. | 129 | 129 | 129 | T4, T5 | Patient records | Survival | Medical records |
| Loh (2006) | | Retrospective review of patient records | To investigate the time interval between the onset of symptoms and first hospital consultation for suspicion of lung cancer and between first hospital consultation and treatment or decision-to-treat or not-to-treat and to examine the association between delay and survival in patients with NSCLC. | Malaysia | Specialist care (2 sites) | Patients with confirmed NSCLC between 1 January 1996 and 1 April 2004 in two urban based hospitals in Malaysia. | 158 | 133 | 122 | T3, T12 | Patient records | Local staging system, consistent with TNM staging Survival | Medical records |
| Maguire (1994) | | Retrospective review of patient records | To evaluate the risk function of the duration of symptoms upon survival after diagnosis, whilst taking into account the effects of such factors as age, sex, tumour site and stage. | Spain | Specialist care (single site) | Patient records from the hospital tumour registry between 1978 and 1989 for seven most common cancers. | 1920 | 1887 | 566 | T4 | Patient records | Staging system:- local, regional, disseminated, unspecified Survival | Medical records |
| Mohan (2006) | | Consecutive patient survey | To assess the baseline Quality of Life (QoL) in newly diagnosed patients with lung cancer to study the impact variables on their QoL | India | Specialist care (single site) | Patients with a histological or cytological diagnosis of lung (small cell or non-small cell type) seen between 30 September 2003 and 1 April 2004. | 76 | 76 | 76 | T4 | Patient survey | WHO QoL -Brief (questionnaire in Hindi) | Patient survey |
| Murai (2012) | | Retrospective review of patient records | To investigate the relationship between wait times and tumour growth and TNM stage progression in patients with lung adenocarcinoma or squamous cell carcinoma undergoing Stereotactic Body Radio Therapy (SBRT) and to identify any possible differences between AD and SQ. | Japan | Specialist care (multi-site) | Patients enrolled in multi-institutional protocol-based SBRT studies. All patients had NSCLC measuring 5cm or less at diagnosis. Patients with histological confirmation of AD or SQ and with clear lung CT images before referral were considered eligible. | 319 | 201 | 135 | T15 | Patient records | Stage | Medical records |
| Myrdal (2004) | | Retrospective review of patient records and cancer registry | To examine the relation between delay and prognosis in patients with NSCLC and to investigate the delay time from first symptom and from first hospital visit to start of treatment | Sweden | Specialist care (multi-site) | Patients diagnosed with NSCLC between 1 January 1995 and 31 December 1999 | 750 | 466 | 354 | T5, T14 | Patient records | TNM Staging Stage I-IV Survival | Medical records |
| Neal (2007) | | Retrospective review of hospital records | To compare outcomes of cancer patients referred through the urgent referral guidance with those who were not with respect to stage at diagnosis, survival and delays in diagnosis | UK  (England) | Specialist care (multi-site) | Data from a 2 year period (2000-2001) for patients with lung cancer within 1 NHS trust were used to identify two groups of patients: urgent referral through GP fast track and those diagnosed through other referral pathways | 889 | 409 | 409 | T11 | Patient records | TNM Staging Stage I-IV Survival | Medical records |
| Pita Fernandez (2003) | | Retrospective review of hospital records | To evaluate the relationship between delayed diagnosis, the degree of invasion and survival in lung cancer | Spain | Specialist care (single site) | All patients diagnosed with lung cancer between 1 January 1997 to 31 December 1998 | Not reported | Not reported | 378 | T4 | Patient records | TNM Staging Stage I-IV Survival | Medical records |
| Radzikowska (2012) | | Retrospective review of patient records | The goal of this study was to assess prospectively the delay due to patients and doctors and its impact on survival of an unselected population of non- small cell lung cancer (NSCLC) patients registered in Pulmonary out patients departments in Poland. | Poland | Specialist care (multi-site) | Squamous cell lung cancer patients and adenocarcinoma patients registered in pulmonary outpatient departments from all parts of Poland collected in the Register of the National Tuberculosis and Lung Diseases Research Institute. | 10586 | 10586 | 7358 | T1, T7, T9, T11 | Patient questionnaire | Survival | Medical records Registry data Death records |
| Salomaa (2005) | | Retrospective review of patient records | To measure delays of diagnosis and to assess the causes for those delays in patients with lung cancer. In addition, the relation of delay times and survival was analysed | Finland | Specialist care (single site) | Patients who were found to have lung cancer at Turku University Hospital, Finland during 2001. | 133 | n/a | 132 | T1, T6, T10, T13, T15 | Patient records | TNM Stage  Stage I-IV Survival | Medical records |
| Skaug (2011) | | Retrospective review of patient records | To examine the long term survival and possible predictors in all patients with lung cancer in a defined geographical area. Also whether and how respiratory and extra-pulmonary symptoms and the delay from symptom onset to diagnosis influenced survival. | Norway | Population based | All new patients in the Norwegian Cancer Registry with lung cancer in International Classification of Diseases (ICD) 7, (1990-1992) and ICD 9 (1993-1996), and all new patients in the hospital records of Haugeshund Hospital with lung cancer in ICD 9. | 576 | 576 | 271 | T4, T6 | Patient records | Survival | Medical records |
| Tokuda (2009) | Retrospective review of patient records | To investigate relative values of symptom-to-visit intervals in patients with cancer and to classify them into groups with homogenous intervals and to examine the relation of the intervals to distant metastasis in patients with common types of solid tumours | Japan | Specialist care | All patients with a diagnosis of cancer registered in the hospital cancer registry database for 10 years from January 1991 through December 2000. | 3893 |  | 490 | T1 | Cancer registry Patient records | Distant metastasis:  Metastasis No metastasis | Medical records |  |
| Tørring (2013) | Prospective cohort study | To assess the association between the length of the diagnostic interval and the ﬁve-year mortality for the ﬁve most common cancers in Denmark while addressing the above methodological and analytical issues. | Denmark | Population based | All patients with newly diagnosed colorectal, lung, melanoma skin, breast or prostate cancer above the age of 17 in the former Danish County of Aarhus during 1 year (inclusion period from 1 September 2004 to 31 August 2005), which was equivalent to 56% of all new cancers in Denmark during that year. | 1543 | 1295 | 1128: Lung=262 (23%) | T8 | Cancer registry  GP Questionnaire | Survival | Registry data Danish Cancer Registry, County Hospital Discharge Registry  Danish Civil Registration System,  GP Questionnaire |  |
| Yilmaz  (2008) | Retrospective review of patient records and patient questionnaire/ interviews | To investigate the delays from the first symptom to thoracotomy and to examine whether the delays cause stage advancement in lung cancer | Turkey | Specialist care (single site) | All patients with primary lung cancer referred from the pneumology departments to first thoracic surgery department between January 2005 and July 2006. | 192 | 138 | 132 | T1, T7, T9, T13, T15 | Patient records Patient interview | TNM Staging system  Stage I-IV | Medical records |  |
| Yun (2012) | Retrospective review of patient records | To investigate the influence of hospital volume, delay of surgery, and both together on the long term survival of postoperative cancer patients. | South Korea | Population based | Patients aged >20 years who had been diagnosed with cancer of the stomach, colon, rectum, pancreas, lung or breast. | 497,339 | 266,328 | 147,682 (this number for all cancers) | T15 | Cancer registry  Health Insurance Review and Assessment Service | Survival | Registry data (Korea Central Cancer Registry), Korea National Statistical Office Database |  |
| **Gastro-intestinal Tract Cancers** | | | | | | | | | | | | |  |
| **Gastric** | | | | | | | | | | | | |  |
| Arvanitakis (1992) | Prospective cohort study | To investigate the reasons for delayed diagnosis of gastric cancer and to correlate survival with early or late diagnosis | Greece | Specialist care (single site) | Selected patients with gastric cancer between 1 August 1983 and 31 July 1986 | 100 | 100 | 100 | T1, T4 | Patient interview (telephone) | Survival  Resectability | Medical records  Direct patient follow-up |  |
| Fernandez (2002) | Prospective cohort study | To characterise the duration of symptoms and to analyse its influence upon the survival of symptomatic patients with cancer of the stomach (oesophagus, colon and rectum) | Spain | Specialist care (single site) | All symptomatic patients newly diagnosed for a cancer of the stomach (also, oesophagus, colon, rectum) first treated between February 1987-February 1989 and June 1991-January 1992. | 70 | 70 | 70 | T4 | Patient records  Patient interview | Tumour stage  Local , regional, disseminated | Medical records |  |
| Haugstvedt (1991) | Prospective cohort study | To investigate factors influencing delay and, secondly, to evaluate the potential consequences of treatment delay on resectability rate and post-operative morbidity and mortality in patients with stomach cancer included in a Norwegian multi-centre study | Norway | Specialist care (multi-site) | All patients included in the Norwegian Stomach Cancer Trial  (a prospective observational study involving 51 surgical units in Norway), accrual was conducted between September 1, 1982 and December 31, 1984. | 1165 | 1165 | 1000(TD) 964(DD)  939 (PD) | T1, T5, T9 | Patient records | TNM stage I-IV  Post-operative mortality Survival status during primary hospital stay Resection vs non-resectional procedure vs no operation | Medical records |  |
| Lim (1974) | Retrospective review of patient records | To examine the correlation between survival in patients with gastrointestinal cancer and the length of preoperative symptoms | USA | Specialist care (multi-site) | All patients with cancer of the stomach (also, colon and rectum)seen between 1966 and 1970 | 111 | 111 | 111 | T3, T14 | Patient records | Survival | Medical records (tumour registry) |  |
| Maconi (2003) | Retrospective review of patient records | To evaluate whether a 6 month delay in diagnosis in gastric cancer patients with uncomplicated dyspepsia without alarm symptoms (i.e. without weight loss, anorexia, GI bleeding, dysphagia, anaemia) would affect overall survival | Italy | Specialist care (multi-site) | All patients without alarm symptoms < 45 years of age diagnosed with gastric cancer between January 1985 and December 2001 | 54 | 54 | 54 | T4 | Patient records | Survival | Medical records |  |
| Maguire (1994) | Retrospective review of patient records | To evaluate the risk function of the duration of symptoms upon survival after diagnosis, whilst taking into account the effects of age, sex, tumour site and tumour stage at diagnosis. | Spain | Specialist care (single-site) | All patients registered with the hospital Tumour Registry between 1978 and 1989 with a diagnosis of stomach cancer (Also: lung, breast, colon, rectal, lymphoma and urinary bladder). | 217 | 217 | 217 | T4 | Patient records | Survival  Tumour stage Local vs Regional vs Disseminated | Medical records  (tumour registry) |  |
| Martin (1997) | Prospective cohort study | To examine the time taken to diagnose gastric or oesophageal cancer, identify the source of delay, and assess its clinical importance | UK  (England) | Specialist care (single-site) | A consecutive series of patients with gastric or oesophageal cancer referred to the General Infirmary at Leeds over 16 months starting in January 1994. | 88 | 88 | 88 | T1, T6, T10, T13 | Patient records  Patient interview | TNM stage  I-IV  Resectability Cure rates | Medical records |  |
| Tokuda (2009) | Retrospective review of patient records | To investigate relative values of symptom-to-visit intervals in patients with cancer and to classify them into groups with homogenous intervals and to examine the relation of the intervals to distant metastasis in patients with common types of solid tumours | Japan | Specialist care (single site) | All patients with a diagnosis of cancer registered in the hospital cancer registry database for 10 years from January 1991 through December 2000. | 314 | 314 | 314 | T1 | Cancer registry Patient records | Distant metastasis Metastasis No metastasis | Medical records |  |
| Windham (2002) | Retrospective review of patient records | To investigate whether early diagnosis would result in more frequent identification of early-stage gastric cancer, leading to improved survival | USA | Specialist care (single site) | All patients aged 35 years or younger with a diagnosis of gastric adenocarcinoma referred between 1976 and 1993 | 127 | 127 | 127 | T4, T8 | Patient records | Survival | Medical records |  |
| Yun (2012) | Retrospective review of patient records | To investigate the influence of hospital volume, delay of surgery, and both together on the long term survival of postoperative cancer patients. | South Korea | Population based | Patients aged >20 years who had been diagnosed with cancer of the stomach, colon, rectum, pancreas, lung or breast. | 497,339 | 266,328 | 147,682 (this number for all cancers) | T15 | Cancer registry  Health Insurance Review and Assessment Service | Survival | Registry data (Korea Central Cancer Registry), Korea National Statistical Office Database |  |
| Ziliotto (1987) | Retrospective review of patient records | To correlate data concerning malignant stomach tumours detected in 189 patients and to evaluate the direct and indirect role of these variables on the evolution and prognosis of gastric cancer | Brazil | Specialist care (single site) | All patients with malignant neoplasias of the stomach treated at the Surgery Service, between January 1960 and December 1978 | 189 | 138 | 138 | T3 | Patient records | Survival | Medical records |  |
| **Oesophageal** | | | | | | | | | | | | |  |
| Fernandez (2002) | Prospective cohort study | To characterise the duration of symptoms and to analyse its influence upon the survival of symptomatic patients with cancer of the oesophagus | Spain | Specialist care (single site) | All symptomatic patients newly diagnosed for a cancer of the oesophagus, stomach, colon, or rectum who were first treated between February 1987 to February 1989 and June 1991 to January 1992. | 31 | 31 | 31 | T4 | Patient interview | Tumour stage  Local  Regional Disseminated | Medical records |  |
| Martin (1997) | Prospective cohort study | To examine the time taken to diagnose gastric or oesophageal cancer, identify the source of delay, and assess its clinical importance | UK  (England) | Specialist care (single site) | A consecutive series of patients with gastric or oesophageal cancer referred to the General Infirmary at Leeds | 27 | 27 | 27 | T1, T6, T10, T13 | Patient interview | TNM stage, Cure rates | Medical records |  |
| Tokuda (2009) | Retrospective review of patient records | To investigate relative values of symptom-to-visit intervals in patients with cancer and to classify them into groups with homogenous intervals and to examine the relation of the intervals to distant metastasis in patients with common types of solid tumours | Japan | Specialist care (single site) | All patients with a diagnosis of cancer registered in the hospital cancer registry database for 10 years from January 1991 through December 2000. | Not reported | 154 | 154 | T1 | Cancer registry  Patient records | Metastasis No metastasis | Medical records |  |
| Wang (2008) | Retrospective review of patient records | To investigate the delay from the first symptom-to-treatment of oesophageal cancer and possible correlation between symptom-to-treatment delay and the stage at the time of treatment | China | Specialist care (single site) | All patients diagnosed as having oesophageal cancer between 1 January to 30 July 2007 | 80 | 80 | 80 | T5 | Patient records  Patient interview | TNM Stage  Differentiation: Well  Moderate  Poor | Medical records |  |
| **Gastric and Oesophageal** | | | | | | | | | | | | |  |
| Grotenhuis (2010) | Prospective cohort study | To test the hypothesis that longer delays between onset of symptoms, endoscopic diagnosis, and surgical treatment are associated with a worse short-term outcome (morbidity, reoperation rate, and in-hospital mortality), worse tumour stage, and hence, worse long-term outcome (overall survival) following potentially curative oesophagectomy in patients with oesophageal cancer. | The Netherlands | Specialist care (single site) | Between January 1991 and December 2007, 791 patients underwent oesophagectomy for cancer of the oesophagus or gastroesophageal junction in the Erasmus MC. | 791 | 491 | 491 | T4, T15 | Patient records | Stage  TNM Survival (Overall survival)   Pre-hospital and hospital delay and: morbidity; reoperation; in-hospital mortality; radicality of resection | Medical records Data on patients' demographics, diagnostic tests, surgery, postoperative morbidity, in-hospital mortality, and survival have been collected prospectively and stored in a database |  |
| Sharpe (2010) | Retrospective review of patient records | The aim of the current study was to compare the outcome of patients with oesophago-gastric carcinoma in relation to their route of referral. This would either confirm or refute the hypothesis that the two week wait referral leads to an improved outcome for patients with oesophago-gastric carcinoma. | UK  (England) | Specialist care (multi-site) | Patients with gastric or oesophageal cancer referred through the gastro-oesophageal multi-disciplinary meeting at University Hospitals of Leicester NHS Trust between 1st January 2006 and 31st December 2007. | Not reported. | 340:  Gastric cancer: 154;  Oesophageal cancer: 186 | 340:  Gastric cancer: 154; Oesophageal cancer: 186 | T12 | Patient records | Survival | Medical records |  |
| **Pancreatic** | | | | | | | | | | | | |  |
| Gobbi (2013) | Retrospective review of patient records | To verify the prognostic role of each symptom, giving particular attention to diagnostic delays and to relationships with other known clinical and pathological factors. | Italy | Not stated | Patients with newly diagnosed pancreatic cancer in the decade 2001-2010. | 170 | 170 | 170 | T4 | Patient records | Survival | Medical records |  |
| McLean (2013) | Retrospective review of patient records | To define the impact of delays in surgery on patients with pancreatic and periampullary malignancies. | Canada | Specialist care (single site) | Patients undergoing pancreatic resection surgery from July 2000 until October 2008. | 355 | 193 | Resected: 119  Unresectable: 74 | T5 | Patient records | Stage Survival Non-resectability | Medical records |  |
| Raptis (2010) | Prospective cohort study | To evaluate whether the nature of clinical presentation (jaundice, abdominal pain, weight loss) and time delay in referring, diagnosing and treating patients with pancreatic cancer had any effect on operability, resectability and survival. | UK  (England) | Specialist care (single site) | Patients with pancreatic cancer admitted to the Pancreatic Unit at the Middlesex Hospital, London, UK in the period between January 1997 and December 2002.Patients with ampullary, peri-ampullary and duodenal cancer, neuroendocrine and cystic pancreatic neoplasms were not included. | 355 | 355 | 355 | T2, T10, T13, T14 | Patient records | Survival | Medical records |  |
| Tokuda (2009) | Retrospective review of patient records | To investigate relative values of symptom-to-visit intervals in patients with cancer and to classify them into groups with homogenous intervals and to examine the relation of the intervals to distant metastasis in patients with common types of solid tumours | Japan | Specialist care (single site) | All patients with a diagnosis of cancer registered in the hospital cancer registry database for 10 years from January 1991 through December 2000. | Not reported | 57 | 57 | T1 | Cancer registry, patient records | Metastasis No metastasis | Medical records |  |
| Yun (2012) | Retrospective review of patient records | To investigate the influence of hospital volume, delay of surgery, and both together on the long term survival of postoperative cancer patients.   | South Korea | Population based | Patients aged >20 years who had been diagnosed with cancer of the stomach, colon, rectum, pancreas, lung or breast. Excluded were patients with multiple cancers and patients who did not undergo cancer surgery as their first definitive treatment. | 497,339 | 266,328 | 147,682 (this number for all cancers) | T15 | Cancer registry  Health Insurance Review and Assessment Service | Survival | Registry data (Korea Central Cancer Registry), Korea National Statistical Office Database |  |
| **Hepatocellular** | | | | | | | | | | | | |  |
| Singal (2013) | Retrospective review of patient records | To characterize and identify factors associated with underuse and delays in treatment among a cohort of patients with hepatocellular carcinoma. | USA | Specialist care (single site) | Patients with cirrhosis diagnosed with hepatocellular carcinoma (HCC) between January 2005 and June 2012. Patients were identified through a combination of ICD-9 codes for HCC, a prospectively maintained list of patients seen in a multidisciplinary liver tumour clinic and tumour  conference presentation lists. | 457 | 267 | 165 | T15 | Patient records | Survival | Medical records |  |
| Tokuda (2009) | Retrospective review of patient records | To investigate relative values of symptom-to-visit intervals in patients with cancer and to classify them into groups with homogenous intervals and to examine the relation of the intervals to distant metastasis in patients with common types of solid tumours | Japan | Specialist care (single site) | All patients with a diagnosis of cancer registered in the hospital cancer registry database for 10 years from January 1991 through December 2000. | Not reported | 251 | 251 | T1 | Cancer registry  Patient records | Metastasis No metastasis | Medical records |  |
| **Colorectal** | | | | | | | | | | | | |  |
| Cerdan-Santacruz (2011) | Patient interview | To determine the current delay in diagnosing colorectal cancer (CRC) and establish whether there has been any improvement in the past 25 years in the same healthcare setting using the same methods | Spain | Specialist care (single site) | Patients admitted for the surgical treatment of CRC | Number of patients excluded not stated | 152 | 152 | T1, T15 | Patient interview | Stage | Medical records |  |
| Currie (2011) | Prospective Cohort | To investigate the influence of hospital volume, delay of surgery, and both together on the long term survival of postoperative cancer patients. | UK | Specialist care (single site) | 125 consecutive patients diagnosed with rectal cancer between January 2000 and December 2005. | 125 | 125 | 125 | T10, T14 | Patient records | Survival | Medical records |  |
| Deng (2012) | Patient interview | To investigate the diagnostic status of colorectal cancer (CRC) and the influence of early diagnosis and cancer stage in a tertiary care hospital in China. | China | Specialist care (single site) | From August 2008 to December 2009, 364 newly diagnosed CRC patients who were consecutively hospitalized for further treatment were recruited and surveyed at a tertiary care hospital | 364 | 307 | 307 | T4, T15 | Patient questionnaire | Stage | Medical records |  |
| Gort (2010) | Retrospective review of patient records  Cancer registry | This study aims to assess the impact of actionable factors on postoperative complications, disease-free survival (local recurrence and distant metastases) and 5-year relative survival in rectal cancer patients. | The Netherlands | Population based | All rectal cancer patients diagnosed in the northern part of the Netherlands from January 2001 to January 2005 who underwent a curatively intended rectal resection for a histologically proven invasive rectal cancer, without distant metastases (pTNM according to UICC classification stages I–III) | 948 | 819 | 819 | T15 | Patient records  Cancer registry | Stage Survival Disease-free survival (DFS) and relative survival (RS) | Medical records Registry data (Cancer Registry of the Comprehensive Cancer Centre North East) |  |
| Guzman (2011) | Prospective cohort study | To evaluate the effectiveness of a fast track diagnosis and treatment program for CRC in terms of diagnosis to treatment interval (DTI) and tumour stage reduction. Secondly to study the association between the DTI and the tumour stage, independently of the caregiving track. | Spain | Specialist care (single site) | 165 incident cases of CRC referred through a preferential pathway between July 2005 and December 2008 in a tertiary hospital were included and 156 patients cared for in the habitual care track (control group). | 165 from FTDTP group, 156 patients from control group | 165 from FTDTP group, 156 patients from control group | 165 from FTDTP group, 156 patients from control group | T14 | Patient records | Stage | Medical records |  |
| Pruitt (2013) | Retrospective review of patient records | We examined the effect of diagnostic and treatment delays on all-cause and colorectal cancer specific death among US adults aged 66 years and older with invasive colon or rectal cancer. We hypothesized that longer delays would be associated with a greater risk of death. | USA | Population based | We selected all male and female patients aged 66 and older with a diagnosis of a first primary invasive colon or rectal cancer occurring from 1998 to 2005 who had full coverage by both Medicare Part A and Part B during this period. | 10663 | 10663 | 10663 | T15 | Patient records  (Linked Surveillance Epidemiology and End Results (SEER) Medicare data) | Stage Survival | Medical records Registry data |  |
| Ramsay (2012) | Prospective cohort study | to establish whether urgency of referral of patients with large bowel malignancy has any effect on management. | UK  (Scotland) | Specialist care (single site) | An analysis was undertaken of all patients with colorectal cancer referred by primary care and discussed at the regional colorectal multi-disciplinary team (MDT) meetings from January 2009 to December 2010. | 485 | 369 primary case referrals with CRC:  303 (82.1%) urgent; 66 (17.9%) routine | 369 primary case referrals with CRC:  303 (82.1%) urgent; 66 (17.9%) routine | T11 | Patient records | Stage | Medical records |  |
| Roland (2013) | Retrospective review of patient records Cancer registry | To demonstrate whether interval to treatment delivery was associated with differences in survival in a cohort of patients with colorectal adenocarcinoma. | USA | Specialist care (multi-site) | All patients diagnosed as having primary colorectal carcinoma at the main campus of UT Southwestern Medical Centre in Dallas, Texas from January 1, 2000, to October 31, 2008. | 1181 | 592 | 592 | T15 | Patient records  Registry data | Survival | Registry data |  |
| Singh (2012) | Cancer registry | To determine recent trends in time to diagnosis for CRC and its association with clinical outcomes. | Canada | Population based | Patients diagnosed with a colorectal adenocarcinoma between January 1, 2004 and March 31, 2009 as their first cancer were identified from Manitoba's population-based Cancer Registry (MCR). | Not reported | 2310 | 2310 | T8 | Cancer registry  . | Stage Survival | Registry data (MCR and the administrative databases maintained by Manitoba Health) |  |
| Terhaar sive Droste  (2010) | Retrospective review of patient records Prospective cohort study Patient interview Cancer registry | To evaluate the association between delay and survival in symptomatic patients with early stage colorectal cancer (CRC) and late stage CRC. | The Netherlands | Population based | All colonoscopies and sigmoidoscopies performed during a three month period in 2005 in the province Northern Holland were evaluated. All consecutive patients diagnosed with symptomatic colorectal cancer were registered. | 376 | 272 | 272 | T1, T5, T6, T9, T11 | GP records  Medical records Patient questionnaire GP Questionnaire | Stage Survival | Medical records Patient questionnaire |  |
| Thompson (2011) | Mixed retrospective and prospective | To determine whether rectal bleeding is related to stage of bowel cancer and whether earlier diagnosis and treatment are associated with improved survival. | UK  (England) | Specialist care (single site) | The Wessex Bowel Cancer Audit identified all patients presenting with bowel cancer in Wessex over a 4-year period from 1 September 1991 to 31 August 1995, including Portsmouth in a 3-year period during 1991–1994. | 845 | 676 | 671 | T4, T5 | Patient records  GP's referral letter and outpatient clinic and inpatient notes | Stage Survival | Medical records |  |
| Tomlinson (2012) | Mixed retrospective and prospective | To identify factors associated with delays to medical assessment and diagnosis for patients with colorectal cancer (CRC). | Canada | Specialist care (single site) | Patients newly diagnosed with a histologically proven colorectal adenocarcinoma were identified and eligible for the study a convenience sample of postsurgical patients with histologically proven colorectal adenocarcinoma was recruited from a tertiary cancer centre in Edmonton, Alta between August 2008 and June 2009. | 93 | 87 | 87 | T1 | Patient records  Patient interview (Confirmation of patient-recalled dates were obtained from the laboratory, imaging, endoscopy, pathology and operative reports) | Stage | Medical records |  |
| Tørring (2011) | Prospective cohort study | The aim of this study was to analyse the association between the diagnostic interval and mortality after diagnosis of CRC, while taking account of the interpretation of symptoms by the general practitioner (GP) and controlling for confounding factors at first presentation. | Denmark | Population based | All newly diagnosed CRC patients over the age of 17 during 1 year (1 September 2004 to 31 August 2005). | 363 | 268 | 268 | T8 | Patient records GP questionnaire | Survival | Registry data  GP Questionnaire |  |
| Tørring (2012) | Retrospective review of patient records Patient interview Cancer registry | To test the theory of a U-shaped association between time from the first presentation of symptoms in primary care to the diagnosis (the diagnostic interval) and mortality after diagnosis of colorectal cancer. | UK Denmark | Primary care; Population based | The authors’ analysed data from three previously described population-based studies in Denmark and the United Kingdom. From each study, the authors included all newly diagnosed CRC patients older than 39 years. | 1667 | 1243 | 1243 | T8 | Patient records Patient questionnaire GP questionnaire | Survival | Medical records Registry data  GP Questionnaire |  |
| Tørring (2013) | Prospective cohort study | To assess the association between the length of the diagnostic interval and the ﬁve-year mortality for the ﬁve most common cancers in Denmark while addressing the above methodological and analytical issues. | Denmark | Population based | All patients with newly diagnosed colorectal, lung, melanoma skin, breast or prostate cancer above the age of 17 in the former Danish County of Aarhus during 1 year (inclusion period from 1 September 2004 to 31 August 2005). | 1543 | 1295 | 1128: CRC=268 (24%) | T8 | Cancer registry  GP Questionnaire | Survival | Registry data Danish Cancer Registry; County Hospital Discharge Registry  Danish Civil Registration System,  GP Questionnaire |  |
| Valentin-Lopez (2012) | Mixed retrospective and prospective | To assess the results achieved with a rapid referral pathway for suspected colorectal cancer (CRC), comparing with the standard referral pathway. | Spain | Primary care; Specialist care (single site) | Patients suspected of having CRC routed via a rapid referral pathway, and patients with CRC routed via the standard referral pathway of a health care district serving a population of 498,000 in Madrid between August 2004 and October 2007. | 272 patients were referred via rapid referral pathway | 252 rapid referral pathway patients finally underwent colonoscopy | 52 of the rapid referral patients were finally diagnosed with CRC; 311 standard pathway patients | T15 | Patient records | Stage  Astler–Coller | Medical records |  |
| Van Hout (2011) | Retrospective review of patient records | To assess patient and doctor delay and their determinants. | The Netherlands | Primary care | All patients registered with an ICPC code D75 (CRC) between 1 January 1997 and 31 December 2007, with a histological confirmed adenocarcinoma of the colon or rectum, and with initial consultation for GI symptoms at the GP were eligible. | 329 | 222 | 222 | T1, T11, T15 | Primary Care Network Utrecht | Stage | Registry data Primary Care Network Utrecht (PCNU) |  |
| Yun (2012) | Retrospective review of patient records | To investigate the influence of hospital volume, delay of surgery, and both together on the long term survival of postoperative cancer patients. | South Korea | Population based | Patients aged >20 years who had been diagnosed with cancer of the stomach, colon, rectum, pancreas, lung or breast. | 497,339 | 266,328 | 147,682 (this number for all cancers) | T15 | Cancer registry  Health Insurance Review and Assessment Service | Survival | Registry data (Korea Central Cancer Registry), Korea National Statistical Office Database |  |
| Zafar (2012) | Retrospective review of patient records | To compare 5-year survival rates in colorectal cancer (CRC) patients who underwent potentially curative surgery before and after the introduction of the 2-week wait (2WW) referral system. | UK  (England) | Specialist care (single site) | Colorectal cancer patients who underwent surgery in the year 1999, i.e. 1 year prior to the introduction of the 2WW referral system. The post-2WW group comprised patients who underwent surgery in the year 2002, i.e. 18 months after the introduction of the 2WW referral system. | Pre 2WW group: 150 Post 2WW group: 126 | Pre 2WW group: 84 Post 2WW group: 64 | Pre 2WW group: 84 Post 2WW group: 64 | T12 | Patient records | Survival | Medical records |  |
| **Renal Tract Cancers** | | | | | | | | | | | | |  |
| **Prostate** | | | | | | | | | | | | |  |
| Korets (2012) | Retrospective review of patient records | To examine the effect of delay from diagnosis to surgery on outcomes in men with localised prostate cancer and analyse the impact of the delay across well described patient risk categories. | USA | Specialist care (single site) | All patients treated with radical prostatectomy by five high-volume surgeons between 1990 and 2010. Records of patients with surgical delays of >180 days were reviewed to ensure that none of the patients had been placed on active surveillance with delayed curative intervention protocols. | 2739 | 1568 | 1568 | T15 | Patient records | Stage Survival | Medical records |  |
| Neal (2007) | Retrospective analysis of hospital records | To compare outcomes of cancer patients referred through the urgent referral guidance with those who were not, with respect to stage at diagnosis, survival, and delay in diagnosis. | UK  (England) | Specialist care (single site) | Data from a 2 year period(2000-2001) for patients with prostate cancer within 1 NHS trust were used to identify two groups of patients: urgent referral through GP fast track and those diagnosed through other referral pathways. | 146 | 136 | 136 (survival)  120 (stage) | T11 | Patient records | Survival  Stage Gleason score | Medical records |  |
| O'Brien (2011) | Retrospective review of patient records | To determine the impact of delaying radical prostatectomy for 6 months or more on surgical pathology and biochemical recurrence rates in a contemporary population of men with low risk prostate cancer. | USA | Specialist care (multi-site) (assumed) | Men with D'Amico low risk prostate cancer who underwent radical prostatectomy by a single surgeon using standardised technique. | 1900 | 1,111 | 1,111 | T15 | Patient records | Stage Survival | Medical records |  |
| Sun (2012) | Cancer registry | To compare the effect of radical prostatectomy delay on postoperative functional outcomes and mortality. | Canada | Population based | Individuals aged >66 years diagnosed with prostate cancer as their first malignant disease who had Medicare A and B and were not enrolled in a health maintenance organisation were abstracted. | 17,153 | 17,153 | 17,153 | T15 | Cancer registry | Stage Survival | Registry data (SEER Medicare database) |  |
| Tokuda (2009) | Retrospective review of patient records | To investigate relative values of symptom-to-visit intervals in patients with cancer and to classify them into groups with homogenous intervals and to examine the relation of the intervals to distant metastasis in patients with common types of solid tumours. | Japan | Specialist care (single site) | All patients with a diagnosis of cancer registered in the hospital cancer registry database for 10 years from January 1991 through December 2000. | 76 | 76 | 76 | T1 | Cancer registry, patient records | Distant metastasis No metastasis | Medical records |  |
| Tørring (2013) | Prospective cohort study | To assess the association between the length of the diagnostic interval and the ﬁve-year mortality for the ﬁve most common cancers in Denmark while addressing the above methodological and analytical issues. | Denmark | Population based | All patients with newly diagnosed colorectal, lung, melanoma skin, breast or prostate cancer above the age of 17 in the former Danish County of Aarhus during 1 year (inclusion period from 1 September 2004 to 31 August 2005). | 1543 | 1295 | 1128: Prostate=187 (17%). | T8 | Cancer registry  GP Questionnaire | Survival | Registry data Danish Cancer Registry; County Hospital Discharge Registry  Danish Civil Registration System,  GP Questionnaire |  |
| **Renal** | | | | | | | | | | | | |  |
| Holmang (2006) | Retrospective review of patient records | To measure the total delay from the day of initial haematuria until surgery and correlate it with survival. | Sweden | Specialist care (multi- site) | The Swedish Cancer Registry was used to identify patients in Western Sweden diagnosed with a malignant ureteral or renal pelvic tumour between 1971 and 1998. | 943 | 394 | 394 | T1, T5, T9 | Patient records | Survival  TNM Stage  Ta,T1 ,T2,T3, T4 | Medical records |  |
| Tokuda (2009) | Retrospective review of patient records | To investigate relative values of symptom-to-visit intervals in patients with cancer and to classify them into groups with homogenous intervals and to examine the relation of the intervals to distant metastasis in patients with common types of solid tumours. | Japan | Specialist care (single site) | All patients with a diagnosis of cancer registered in the hospital cancer registry database for 10 years from January 1991 through December 2000. | Not reported | 74 | 74 | T1 | Cancer registry  Patient records | Metastasis No metastasis | Medical records |  |
| **Bladder** | | | | | | | | | | | | |  |
| Gulliford (1991) | Retrospective cohort study | To determine whether, after allowing for the severity of the underlying disease, survival of patients with cancer of the bladder in the South Thames regions was influenced by processes of care. | UK  (England) | Specialist care (multi-site) | Men aged under 75 years, resident in the South Thames Region and registered as new cases with the South Thames Cancer Registry in 1982. | 574 | 430 | 430 | T10, T12, T14 | Patient records | Survival | Medical records  Thames Cancer Registry |  |
| Hollenbeck (2010) | Cancer registry | To evaluate relations between a delay in diagnosis and outcomes among patients with bladder cancer. | USA | Population based | From SEER-Medicare linked files, all Medicare patients aged ≥66 years with incident cases of bladder cancer were identified by the appropriate code in SEER. The database for the years 1992 through 2002 was used to identify patients with bladder cancer. | 37,972 | 29,740 | 29,740 | T4 | Cancer registry | Survival | Registry data  Surveillance, Epidemiology, and End Results (SEER)-Medicare linked database |  |
| Liedberg (2003) | Retrospective review of patient records | To correlate diagnostic delay and disease specific survival with different tumour stages and evaluate whether earlier diagnosis influences survival. | Sweden | Specialist care (multi-site) | Cases of invasive bladder cancer T1-T4 from the Southern Swedish Health Care Region notified to the population based Regional Tumour Registry in 1988. | 393 | 193 | 177 | T1, T8 | Regional tumour registry | Survival  Tumour stage  T1 vs T2- T4 | Medical records Regional Tumour Registry  Cause of Death Register |  |
| Maguire (1994) | Retrospective review of patient records | To assess the possible forms of the risk function of symptom to diagnosis interval (SDI) upon cancer survival. | Spain | Specialist care (single site) | Symptomatic cases registered in the Tumour registry between 1978 and 1989 were selected for the study. | 144 | 136 | 136 | T4 | Patient records | Survival | Medical records Tumour registry |  |
| Mommsen (1983) | Prospective cohort study | To elucidate causes on delay, on the assumption that shortening of delay could improve survival in bladder cancer. | Denmark | Specialist care (single site) | Consecutive patients with newly diagnosed bladder tumour admitted to the Department of Urological Oncology and Radiotherapy during a three year period beginning in September 1977. | 212 | 212 | 211 | T1, T5, T7, T14 | Patient interview  GP records | Survival  TNM Stage  T1-T2, T3, T4 | National Health Board  Medical records |  |
| Tokuda (2009) | Retrospective review of patient records | To investigate relative values of symptom-to-visit intervals in patients with cancer and to classify them into groups with homogenous intervals and to examine the relation of the intervals to distant metastases in patients with common types of solid tumours. | Japan | Specialist care (single site) | All patients with a diagnosis of cancer registered in the hospital cancer registry database for 10 years from January 1991 through December 2000. | 83 | 83 | 83 | T1 | Patient records  Cancer registry | Metastases No metastases | Medical records |  |
| Wallace (2002) | Prospective cohort study | To assess in detail and evaluate the effect on survival of delays and component delays in the diagnosis and treatment of bladder cancer. | UK  (England) | Specialist care (multi- site) | Prospective data on all newly diagnosed cases of urothelial cancer in the West Midlands from 1 January 1991 to 30 June 1992. | Not reported | 1537 | 1500 (T2) 1504 (T10) 1504 (T14) 1504 (T12)  1511 (T5) | T2, T5, T10, T12, T14 | Patient questionnaire | Survival  TNM stage pTa,  pT1 T2-T4 | West Midlands Cancer Intelligence Unit  Radiotherapy departments |  |
| **Testicular** | | | | | | | | | | | | |  |
| Adkas (1986) | Retrospective review of patient records | To examine the possible effects of delay in diagnosis and treatment in advanced (Stage III) disease. | Turkey | Specialist care (single site) | Selected patients diagnosed and treated between January 1979 and September 1984 in the Department of Urology. | Not reported | 29 | 29 | T4, T5 | Patient records | Survival rates Stage level  (not defined) | Medical records |  |
| Bosl (1981) | Retrospective review of patient records | To define the extent and causes of delay in diagnosis and to assess the impact of delay on the stage of the disease. | USA | Specialist care (multi-site) | All patients diagnosed with testicular cancer between January 1941 and September 1978 . | 335 | 335 | 335 | T1, T8, T4 | Patient records | Stage I-III | Medical records |  |
| Chilvers (1989) | Retrospective review of patient records | To investigate the relationship between delays in diagnosis and tumour stage, serum marker levels, tumour volume and MRC prognostic group. | UK  (England) | Specialist care (single site) | Patients diagnosed to have testicular teratoma between 1 January 1980 and 31 December 1986. | 257 | 257 | 257 | T5 | Patient records | Survival  Stage I-IV  (Royal Marsden Hospital Staging System) | Medical records |  |
| Dieckmann (1987) | Retrospective review of patient records | To investigate the influence of diagnostic delay on the prognosis of testicular germ cell tumours on the basis of the patient population of the department and a literature review. | Germany | Specialist care (single site) | All patients with testicular germ cell tumours treated between 1969 and 1986. | 180 | 174 | 151 | T5 | Patient records | Stage I-III  (modified classification of staging - Boden and Gibb) | Medical records |  |
| Fossa (1981) | Retrospective review of patient records | To study the history, and the initial symptoms and signs of the disease in patients with malignant germ cell tumours, with special reference to the patient's delay and the doctor's delay as prognostic factors. | Norway | Specialist care (single site) | All patients with malignant germ cell tumours admitted during 1978. | 103 | 103 | Patient delay: 101  Doctor delay: 102  Duration of symptoms:  103 | T1, T8, T4 | Patient records | Survival  Stage I - III | Medical records |  |
| Hanson (1993) | Retrospective review of patient records | To determine predictors of prognostic significance for patients with nonsemitomous testicular cancer who have advanced disease at the time of presentation. | Canada | Specialist care (single site) | All patients with advanced nonsemitomous testicular cancer treated between 1980 and 1990. | Not reported | 33 | 33 | T1 | Patient records | Survival | Medical records Personal communication with patient or physician |  |
| Harding (1995) | Retrospective review of patient records | To ascertain whether delayed diagnosis or type of orchidectomy affected outcome for men with non-seminomatous germ cell tumours. | UK (Scotland) | Population-based (West of Scotland cancer registry) | All males with a diagnosis of teratoma between 1 January 1975 and 31 December 1989, resident in the West of Scotland Health Board areas. | 442 | 442 | 406 | T4 | Patient records | Survival  MRC staging Stage I: localised Metastatic: good or poor prognosis | Medical records |  |
| Huyghe (2007) | Retrospective review of patient records  Prospective patient survey | To describe diagnostic features, analyse trends in diagnostic delay over the study period and assess the influence of diagnostic delay on survival. | France | Specialist care (multi-site) | All patients diagnosed with germ cell tumours from 1983 to 2002 at health facilities in the Mid-Pyrenees region. | 542 | 439 | 439 | T4 | Patient records Patient questionnaire | Survival  Stage I-III  (Boden and Gibb Classification) | Medical records |  |
| Meffan (1991) | Retrospective review of patient records | To assess the effect of length of history on both tumour stage and survival and to determine whether early recognition is likely to improve prognosis. | New Zealand | Specialist care (single site) | All patients with testicular cancer who were diagnosed and treated between 1976 and 1985. | 79 | 79 | 79 | T5 | Patient records | Survival  Stage I: early  II-IV: advanced  (Royal Marsden Hospital Staging System)  Diameter in centimetres | Medical records |  |
| Moul  (1990) | Retrospective review of patient records | To assess the impact of delayed diagnosis on presenting stage and long term survival. | USA | Specialist care (single site) | All testicular cancer patients registered at the cancer centre tumour registry between 1970 and 1987. | 244 | 148 | 148 | T4 | Patient records | Survival  Stage I-III (as per convention) | Medical records |  |
| MRC Working Party (1985) | Retrospective review of patient records | To identify prognostic criteria for prospective randomised-treatment studies in which low-toxicity chemotherapy for good-prognosis patients and more effective chemotherapy for high risk patients will be evaluated. | UK  (England) | Specialist care (multi-site) | Selected patients with histologically proven non-seminomatous testicular germ-cell tumours between January 1976 and June 1982. | 458 | 448 | 448 | T5 | Patient records | Survival  Stage I-IV  (Royal Marsden Hospital Staging System) | Medical records |  |
| Napier (2000) | Retrospective review of patient records | To examine the relationship between diagnostic delay, and subsequent risk of relapse and outcome in patients with stage I nonseminomatous germ cell tumour (NSGCT) followed by active surveillance. | UK  (England) | Specialist care (single site) | Selected patients with Stage I primary testicular NSGCT who were diagnosed between January 1983 and December 1998. | 185 | 185 | 185 | T5 | Patient records | Date of orchidectomy to date of radiological or confirmed marker relapse | Medical records |  |
| Prout (1984) | Retrospective review of patient records | To investigate if , by improving delay times, survival of patients with germ cell tumours would improve. | USA | Specialist care (single site) | Selected patients with a germ cell tumour or history of such a tumour who were seen between 1970 and 1981. | 161 | 89 | 89 | T5 | Patient records | Survival  Presence of metastatic disease | Medical records |  |
| Scher (1983) | Retrospective review of patient records | To determine whether or not the symptomatic interval was related to response to chemotherapy and extent of disease in patients presenting with advanced Stage III disease. | USA | Specialist care (single site) | All patients with Stage III testicular cancer treated between September 1972 and January 1979. | 123 | 123 | 123:  (extent of disease)  119: (treatment response) | T4 | Patient records | Complete response to treatment or not.  Absent, non-palpable mass Palpable mass | Medical records |  |
| Ware (1980) | Retrospective review of patient records | To review clinical and pathological data in order to check if duration of symptoms correlated with stage of disease. | USA | Specialist care (multi-site) | Selected patients with testicular germ cell tumour treated from 1965 through 1977. | 111 | 100 | 100 | T5 | Patient records | Stage I-III (Staging system based on combination clinical and pathological features) | Medical records |  |
| Wishnow (1990) | Retrospective review of patient records | To determine the potential role of prompt diagnosis and orchiectomy in reducing morbidity and mortality. | USA | Specialist care (single site) | Consecutive series patients with non-seminomatous germ-cell testicular tumours between 1 June 1983 and 31 December 1986. | 154 | 154 | 154 | T5 | Patient records | Survival  Stage I-III (Modified Boden and Gibb 1961) Presence or absence of metastasis | Medical records |  |
| **Upper Tract Urothelial** | | | | | | | | | | | | |  |
| Sundi (2012) | Retrospective review of patient records | In this study, we sought to evaluate whether a delay in time to extirpative surgery had any effect on survival of these patients with UTUC. | USA | Specialist care (single site) | Patients with UTUC treated with nephroureterectomy or ureterectomy at the University of Texas M. D. Anderson Cancer Centre between 1990 and 2007. | 247 | 240 | 240 | T15 | Patient records | Survival | Medical records |  |
| Waldert (2010) | Retrospective review of patient records | To evaluate the effect of the duration from diagnosis to definitive surgery on disease progression and clinical outcomes in patients with upper tract urothelial carcinoma. | Location not specified:  data was obtained from a database covering three institutions | Specialist care (multi-site) | 187 patients with a diagnosis of upper-tract urothelial carcinoma treated with radical nephroureterectomy between January 2000 and December 2007 at 3 institutions. | 187 | 187 | 187 | T15 | Patient records | Stage Survival | Medical records |  |
| **Gynaecological Cancers** | | | | | | | | | | | | |  |
| **Cervical** | | | | | | | | | | | | |  |
| Fruchter (1981) | Consecutive patient survey | To examine the impact of delay on stage at diagnosis in gynaecologic cancer. | USA | Specialist care (single site) | All symptomatic patients with new histologic diagnoses of carcinoma cervix between July 1976 and December 1979. | 120 | 120 | 120 | T3, T4, T13 | Patient interview Patient records | FIGO  Stage I-IV | Medical records |  |
| Tokuda (2009) | Retrospective review of patient records | To investigate relative values of symptom-to-visit intervals in patients with cancer and to classify them into groups with homogenous intervals and to examine the relation of the intervals to distant metastasis in patients with common types of solid tumours. | Japan | Specialist care (single site) | All patients with a diagnosis of cancer registered in the hospital cancer registry database for 10 years from January 1991 through December 2000. | 201 | 201 | 201 | T1 | Cancer registry  Patient records | Distant  metastasis No metastasis | Medical records |  |
| Umezu (2012) | Retrospective review of patient records | To identify the prognostic factors in a group of patients with stage IA-IIA cervical cancer who underwent hysterectomy, and to evaluate differences in the recurrence-free and overall survival rates based on the waiting time from the initial visit to a gynaecologist until the date of radical hysterectomy. | Japan | Specialist care (single site) | The study population consisted of stage IA-IIA cervical cancer patients who were treated between September 1999 and June 2010. Each of these patients underwent radical hysterectomy and systematic pelvic lymphadenectomy at Nagoya University Hospital, Japan. | 177 | 177 | 177 | T14 | Patient records | Survival | Medical records |  |
| **Endometrial** | | | | | | | | | | | | |  |
| Crawford (2002) | Retrospective review of patient records | To investigate links between delays in treatment and survival. | UK (Scotland) | Population-based | All women resident in Scotland who were diagnosed with endometrial cancer between January 1996 and December 1997. | 781 | 714 | 618 | T12 | Patient records | Survival  FIGO Stage level | Medical records |  |
| Elit (2013) | Cancer registry | To investigate whether wait time from diagnosis of uterine cancer to definitive surgery (by hysterectomy) was associated with poorer overall survival when accounting for patient factors, tumour factors, and structural factors. | Canada | Specialist care (multi-site) | All patients with uterine cancer who had a hysterectomy in Ontario, Canada, and were diagnosed between April 2000 and March 2009. | 14,225 | 9,417 | 9,417 | T15 | Cancer registry | Survival | Registry data |  |
| Franceschi (1983) | Consecutive patient survey | To evaluate how patterns of health care perception and delivery may interact with risk factors influencing disease evaluation and outcome. | Italy | Specialist care (single site) | All cases of endometrial cancer admitted to an obstetric /gynaecological clinic between January 1978 and December 1980. | 179 | 173 | 161 | T4 | Patient interview | Undefined staging and grading system  Stage, Invasion, Differentiation | Medical records |  |
| Fruchter (1981) | Consecutive patient survey | To examine the impact of delay on stage at gynaecological diagnosis. | USA | Specialist care (single site) | All symptomatic patients with a new histologic diagnoses of adenocarcinoma of the endometrium from July 1976 to December 1979. | 146 | 146 | 146 | T3, T4, T13 | Patient interview Patient records | FIGO  Stage level | Medical records |  |
| Menczer (1995) | Retrospective review of patient records | To assess the association of diagnosis and treatment delay with established prognostic factors and survival. | Israel | Specialist care (single site) | All women diagnosed with endometrial cancer between 1970 and 1986. | 204 | 181 | 181(delay in diagnosis)  174 (treatment delay/total delay) | T4, T5, T15 | Patient records | Survival  FIGO Stage | Medical records |  |
| Obermair (1996) | Retrospective review of patient records | To evaluate the influence of delayed diagnosis on prognostic factors in endometrial cancer. | Austria | Specialist care (single site) | Selected (from records) postmenopausal women with no history of hormonal replacement therapy use. | Not reported | Not reported | 116 | T4 | Patient records | FIGO  Stage level, Vessel invasion,  Histological  grade | Medical records |  |
| Pirog (1997) | Retrospective review of patient records | To evaluate the relationship between late presentation and the stage at presentation of patients with endometrial cancer. | USA | Specialist care (single site) | Selected records of patients treated for endometrioid type of uterine carcinoma. | 220 | 182 | 165 | T4 | Patient records | FIGO  Stage level | Medical records |  |
| Robinson (2012) | Consecutive patient survey | To investigate the association between diagnostic delay (total delay), quality of life (QoL) and patient satisfaction, and the associations between QoL and patient satisfaction scores and survival for women diagnosed with ovarian or endometrial cancer. | Denmark | Specialist care (multi-site) | All women who received their first diagnosis of cancer between 1 October 2006 and 1 December 2007 in four out of the five specialized centres for gynaecological cancer surgery in Denmark. | 723 | 453 | Total: 353  Endometrial: 165 | T5 | Patient questionnaire | Quality of life (EORTC-C30 QoL) and patient satisfaction | Medical records  Patient questionnaire |  |
| Tokuda (2009) | Retrospective review of patient records | To investigate relative values of symptom-to-visit intervals in patients with cancer and to classify them into groups with homogenous intervals and to examine the relation of the intervals to distant metastasis in patients with common types of solid tumours. | USA | Specialist care (single site) | All patients with a diagnosis of cancer registered in the hospital cancer registry database for 10 years from January 1991 through December 2000. | 98 | 98 | 98 | T1 | Cancer registry  Patient records | Distant metastasis No metastasis | Medical records |  |
| **Ovarian** | | | | | | | | | | | | |  |
| Fruchter (1981) | Consecutive patient survey | To examine the impact of delay on stage at diagnosis of gynaecological cancer | USA | Specialist care (single site) | All patients with carcinoma of the ovary diagnosed from 1970 to 1979 | 80 | 80 | 80 | T3, T4, T13 | Patient interview  Patient records | FIGO  Stage I-IV | Medical records |  |
| Lurie (2010) | Patient interview  Cancer registry | To evaluate the association of symptoms with stage at diagnosis, ethnicity, and tumour histological type and grade. | USA | Population based | Women 19-88 years of age, who were diagnosed with histologically confirmed primary invasive epithelial ovarian carcinoma between 1993 and 2008. | 622 | 622 | 622 | T4 | Patient questionnaire  Telephone interview | Stage | Medical records |  |
| Menczer (2009) | Prospective cohort | To assess the association between duration of symptoms and main prognostic factors of invasive epithelial ovarian cancer (EOC). | Israel | Population based | All incident cases of histologically confirmed cancer of the ovary diagnosed between 1 March 1994 and 30 June 1999 identified within the framework of a nation-wide case-control epidemiological study. | 371 | 371 | 187 | T4 | Patient records | Stage I-IV | Medical records |  |
| Nagle (2011) | Prospective cohort study  Consecutive patient survey | To test the assumption that shorter time to diagnosis leads to a more favourable stage at diagnosis and improved survival by examining the association between time to diagnosis, stage of disease at diagnosis, and survival among a large, representative group of Australian women with symptomatic ovarian cancer. | Australia | Population based | All women age 18 to 79 years with a suspected diagnosis of invasive or borderline epithelial ovarian, fallopian tube, or primary peritoneal cancer between January 2002 and June, 2005, identified through the gynaecologic oncology units and state-based cancer registries. | Identified: 3,550  Sampled: 2,745 | Agreed to take part: 2,319 | 1,463 | T1, T4, T8 | Patient interview | Stage Survival | Medical records |  |
| Neal (2007) | Retrospective analysis of hospital records | To compare outcomes of cancer patients referred through the urgent referral guidance with those who were not, with respect to stage at diagnosis, survival and delays in diagnosis. | UK  (England) | Specialist care (single site) | Data from a 2 year period(2000-2001) for patients with ovarian cancer within 1 NHS trust were used to identify two groups of patients: urgent referral through GP fast track and those diagnosed through other referral pathways (lung, colo-rectal, prostate were also studied). | 95 | 95 | 58(survival)  45(stage) | T11 | Medical records | Staging system not defined.  Stage I-IV Survival Delays | Medical records |  |
| Robinson (2012) | Consecutive patient survey | To investigate the association between diagnostic delay (total delay), quality of life (QoL) and patient satisfaction, and the associations between QoL and patient satisfaction scores and survival for women diagnosed with ovarian or endometrial cancer. | Denmark | Specialist care (multi-site) | All women who received their first diagnosis of cancer between 1 October 2006 and 1 December 2007 in four out of the five specialized centres for gynaecological cancer surgery in Denmark. | 723 | 453 | Total: 353  Ovarian: 188 | T5 | Patient questionnaire | Quality of life (EORTC-C30 QoL) and patient satisfaction | Patient questionnaire |  |
| Smith (1985) | Consecutive patient survey | To evaluate characteristics of symptoms, their perceived cause and delay in seeking a diagnosis associated with stage, grade and histologic features of the disease at diagnosis. | USA | Population based (Cancer registry) | Patients identified from NCI -SEER Cancer Registry who took part in the Centers for Disease Control Cancer and Steroid Hormone Study, recently diagnosed (1-3 months) aged 20-54, white. Diagnosed with 1st primary between 1.11.80 and 31.12.82. | 107 | 82 | 56 | T1 | Two pre-tested standardised questionnaires. Cancer Registry | SEER Summary Staging Guide  Stage: local or distant | Cancer Registry  (California Tumor Registry) |  |
| Tokuda (2009) | Retrospective review of patient records. | To investigate relative values of symptom-to-visit intervals in patients with cancer and to classify them into groups with homogenous intervals and to examine the relation of the intervals to distant metastasis in patients with common types of solid tumours. | Japan | Specialist care (single site) | All patients with a diagnosis of cancer registered in the hospital cancer registry database for 10 years from January 1991 through December 2000. | 58 | 58 | 58 | T1 | Cancer registry Patient records | Distant metastasis No metastasis | Medical records |  |
| **Head and Neck** | | | | | | | | | | | | |  |
| Alho (2006) | Population-based retrospective cohort study | To determine the prevalence of symptoms and the association between detection patterns of head and neck cancer in primary care and survival. | Finland | Specialist care (Single site) | Patients diagnosed with histologically verified squamous cell carcinoma of Head and Neck (tongue, pharynx and larynx) between Jan 1986 to Dec 1996. | 318 | 221 | 221 | T1, T8 | Patient medical and dental charts | Survival | Medical and dental records |  |
| Allison (1998) | Prospective patient survey | To test the hypothesis that, controlling for tumour site, patient, professional and/or total diagnostic delays are associated with disease stage at diagnosis among a sample of upper aero digestive tract (UADT) cancer patients. | Canada | Specialist care (multi- site) | Patients diagnosed with squamous cell carcinoma of oral cavity sites, oro-, naso- and hypopharynx and larynx during an 18 month period beginning 1 July 1995. | Not reported | 188 | 188 | T1, T3, T7 | Patient interview (standardised questionnaire) | TNM Stage | Medical records |  |
| Al-Rajhi (2009) | Prospective patient survey | To determine the time lapse between the onset of tumour-related symptoms and the time of presentation to the tertiary care centre to identify the factors contributing to nasopharyngeal carcinoma (NPC) diagnostic delay and to evaluate the impact of delay on tumour staging. | Saudi Arabia | Specialist care (single site) | All newly diagnosed, untreated patients with NPC who attended the combined head and neck oncology outpatient clinic between January 2000 and December 2003. | 307 | 307 | 307 | T1, T4, T8 | Patient interview (structured, face-to-face interviews) | TNM Stage | Nasopharyngeal carcinoma database |  |
| Brouha (2000) | Retrospective review of patient records | To ascertain whether waiting times have a measurable effect on the outcome of T1N0M0 glottic laryngeal tumours treated by radiotherapy. | The Netherlands | Specialist care (single site) + affiliated centres | Patients with T1 squamous cell carcinoma of the glottic larynx irradiated at the radiotherapy department between 1980 to 1996. | 362 | 361 | 360 | T4, T15 | Patient records | Survival  (5 year recurrence free) | Medical records |  |
| Brouha  (2005a) | Prospective patient survey using semi-structured interviews | To determine the length of stages of patient delay in patients with laryngeal cancer and to find out whether these delays are related to the stage of the disease at diagnosis. | The Netherlands | Specialist care (single site) + affiliated centres | Newly diagnosed patients with a squamous cell carcinoma of the larynx during 2000 to 2002 with no previous or synchronous malignancies in the head and neck region. | 169 | 117 | 117 | T1 | Patient interview GP questionnaire  Close relative questionnaire | TNM Stage T Stage | Medical records |  |
| Brouha  (2005b) | Prospective patient survey using semi-structured interviews | To determine the length of stages of patient delay in patients with pharyngeal and oral cancer and to find out whether these delays are related to the stage of the disease at diagnosis. | The Netherlands | Specialist care (single site) + affiliated centres | Newly diagnosed patients with a squamous cell carcinoma of the oropharynx, hypopharynx or oral cavity with no previous or synchronous malignancies in the head and neck during 2000 to 2002. | 258 | 189 | 189 (55 Pharyngeal, 134 Oral) | T1 | Patient interview  GP/Dentist questionnaire  Close relative questionnaire | TNM Stage  ICD-O stage | Medical records |  |
| Caudell (2011) | Retrospective review of patient records | To assess whether the diagnosis-to-treatment interval (DTI) affected outcomes in cases involving patients with locoregionally advanced head and neck cancer. | USA | Specialist care (single site) | Patients with squamous cell carcinoma of the head and neck treated with radiotherapy. | 781 | 781 | 427 | T15 | Patient records | Survival Locoregional control (LRC); Distant metastasis-free survival (DMFS) | Medical records |  |
| Hansen (2005) | Retrospective review of patient records | To study the relationship between the durations of symptoms before the start of radiotherapy and treatment outcome in Stage I–III glottic cancer. | Denmark | Specialist care (single site) | Series of patients treated with radical radiotherapy for glottic cancer between 1965 to 1997. | 611 | 544 | 544 | T5 | Patient records | Survival (5-year recurrence-free) | Medical records |  |
| Ho (2004) | Retrospective review of patient records | To evaluate the prognostic impact of presentation-to-diagnosis interval (PDI) and its association with other clinical factors in patients with oropharyngeal squamous cell carcinoma (OpSCC). | USA | Specialist care (single site) | A consecutive sample of patients diagnosed with carcinoma of the oropharynx and treated with aggressive therapy with curative intent from March 1994 to August 2001. | 124 | 87 | 87 | T4 | Patient and referral records | TNM stage Survival (4-year overall) | Medical records |  |
| Koivunen (2001) | Retrospective review of patient records | To study the impact of patient and professional delay on survival. | Finland | Specialist care (single site) | Patients diagnosed with pharyngeal cancer during January 1986 to December 1996 identified from the registers of the tertiary center, from Statistics Finland and from the cause of death statistics. | 96 | 84 | 84 | T1, T4, T8 | Primary health centres and the private medical and dental practitioner records | Survival  Stage | Tertiary care registry data Statistics Finland Cause of death statistics |  |
| Kumar (2001) | Prospective patient survey | To analyse the psychosocial factors related to delay in presentation of oral cancer patients through a health risk-taking behavioural model and also examine the relationship between delay and the stage of cancer. | India | Specialist care (single site) | Consecutive oral cancer patients who presented at the department of surgery. | 79 | 79 | 79 | T1, T7, T14 | Patient interview (using piloted questionnaires) | TNM Stage | Patient records |  |
| Lee (1997) | Retrospective review of patient records | To review the presenting features of nasopharyngeal carcinoma and the detrimental effect of a delay in presentation on the final outcome. | Hong Kong | Specialist care (single site) | Patients with undifferentiated or non-keratinising carcinoma of the nasopharynx who were treated during 1 Jan 1976 to 31 Dec 1985. | 5020 | 4768 | 4768 | T4 | Patient records | Survival (10-year actuarial disease-specific)  TNM stage | Medical records |  |
| McGurk (2005) | Prospective series of consecutive patients compared with a historical cohort | To establish if there is a positive correlation between increased delay in diagnosis and advanced stage at presentation of mouth and throat cancers. | UK  (England) | Specialist care (single site) | Two cohorts of patients treated between 1961-1986 and 1992-1999 respectively with a diagnosis of squamous cell carcinoma (SCC) of the mouth and throat. | 1296:  930:  Retrospective  366:  Prospective | 1027:  695:  Retrospective  332: Prospective | 605:  400:  Retrospective  205:  Prospective | T1, T10, T14 | Patient records Patient interview | Stage T1,T2,T3, T4 Survival | Medical records |  |
| Miziara (1998) | Retrospective review of patient records | To understand the correlation between clinical characteristics, site of origin, stage of the tumour, histology and the influence of diagnostic delay on the prognosis. | Brazil | Specialist care (single site) | Case records of histologically diagnosed cancers of larynx registered at the Department of Otolaryngology- Clinic from 1985 to 1995. | Not reported | 108 | 108 | T4 | Patient records | Tumour size and nodal involvement | Medical records |  |
| Pitchers (2006) | Retrospective review of patient records | To investigate delay in referral, (from symptom- onset to the date of GPs referral letter to secondary care)and it’s relation to tumour stage and survival. | UK  (England) | Specialist care (single site) | Patients presenting with oropharyngeal squamous carcinoma over the last 10 years between 1995 and 2005 at the Department of Oncology of author's institution. | 110 | 69 | 69  (67 stage) | T1, T6, T10, T13, T15 | Patient records (referral letter , case notes from first outpatient consultation, oncology notes) | TNM stage Survival | Medical records |  |
| Scott (2005) | Consecutive patient survey | To investigate the relationship between diagnostic delay and stage of disease to determine which of the two explanations is more plausible. | UK  (England) | Specialist care (single site) | Consecutive patients with untreated oral squamous cell carcinoma and who did not have a history of cancer who were referred to one oral surgeon between 1992 and 2003. | 245 | 245 | 245 | T4 | Patient interview | TNM Stage | Medical records  Clinical examination |  |
| Seoane (2010) | Mixed retrospective and prospective | To study whether diagnostic delay influences survival to oral cancer when tumour proliferative activity is considered. | Spain | Not stated (assume speciality care single site) | Pathologically diagnosed consecutive incident cases of primary oral squamous cell carcinoma diagnosed between December 1997 and March 2002. | 63 | 63 | 63 | T4 | Patient records | Survival | Medical records |  |
| Sheng (2008) | Retrospective review of patient records | To investigate the related factors leading to patient related diagnostic delay (PRDD) and to evaluate the relationship between PRDD and the degree of invasion, nodal status and disease stage in nasopharyngeal carcinoma patients. | China | Specialist care (single site) | Newly diagnosed and histologically confirmed nasopharyngeal carcinoma patients between Jan 2002 to Dec 2006. | 305 | 216 | 216 | T1 | Patient records | TNM stage T Stages I, II, III & IV  N Stages 0,I,II,III | Medical records |  |
| Sidler (2010) | Retrospective review of patient records | To characterize patients treated for naso-pharyngeal cancer (NPC) in a single institution and to identify important prognostic factors with an impact on overall survival. | Switzerland | Specialist care (single site) | Patients with histologically proven naso-pharyngeal carcinoma. All patients received megavoltage radiation therapy. From 1990-1998, an adjuvant chemotherapy with cisplatin was offered. After 1998, all patients received a concurrent radiochemotherapy with cisplatin. | 34 | 34 | 29 (5 lost to follow up) | T15 | Patient records | Survival | Medical records |  |
| Teppo (2003) | Retrospective review of patient records | To determine the effects of patient and professional diagnostic delays on survival in patients with laryngeal squamous cell carcinoma (LSCC). | Finland | Specialist care (single site) | All patients diagnosed with laryngeal squamous cell carcinoma (LSCC) identified between 1990 and 1995. | 99 | 66 | 66 | T1, T4, T8 | Registry data | TNM stage Survival | Medical records  Registry data  National Death Register |  |
| Teppo (2005) | Retrospective review of patient records | To evaluate the impact of patient and professional diagnostic delays on the risk of recurrence in laryngeal squamocellular carcinoma (LSCC). | Finland | Specialist care (multi-site) | All patients with invasive laryngeal squamocellular carcinoma diagnosed histologically between 1 January 1990 and 31 December 1995. | 99 | 66 | 66 | T1, T4, T8 | Patient records | Risk of recurrence:  No, Local, Neck Distant | Medical records |  |
| Teppo (2008) | Population-based retrospective cohort study | To evaluate the relative importance of patient and professional diagnostic delays in the prognosis of tongue, pharynx and larynx cancers. | Finland | Specialist care (single site) | All patients with histologically verified squamous cell carcinoma of tongue, pharynx or larynx diagnosed between Jan 1986 and Dec 1996. | 318 | 221 | 221 | T1, T8 | Patient medical and dental records | Survival  (2 and 5 year from diagnosis) | Medical records |  |
| Teppo (2009) | Retrospective review of patient records | To characterise the current delay in vestibular schwannoma diagnosis, and to evaluate its impact on tumour size and symptoms at the time of diagnosis, as well as on symptoms or disabilities after treatment. | Finland | Specialist care (multi-site) | All consecutive patients diagnosed on MRI as having a schwannoma of the VIIIth cranial nerve within one district between 1998 and 2006 and within another district between 1992 and 2006. | 91 | 59 | 59 | T1, T4, T8 | Patient records | Tumour size in centimetres on MRI Hearing loss Tinnitus Vertigo Deafness Tinnitus Vertigo Headache/pain Facial weakness | Medical records |  |
| Tokuda (2009) | Retrospective review of patient records | To investigate relative values of symptom-to-visit intervals in patients with cancer and to classify them into groups with homogenous intervals and to examine the relation of the intervals to distant metastasis in patients with common types of solid tumours. | Japan | Specialist care (single site) | All patients with a diagnosis of cancer registered in the hospital cancer registry database for 10 years from January 1991 through December 2000. | 303 | 303 | 303 | T1 | Cancer registry  Patient records | Distant  metastasis No metastasis | Medical records |  |
| Tromp (2005) | Prospective patient survey | To test the different forms of delay for their independent effect on tumour size  and to evaluate the relationship of several patient-related and tumour-related factors with tumour size at diagnosis. | Belgium | Specialist care (single site) | Newly diagnosed patients with squamous cell carcinoma of the larynx, pharynx or oral cavity during a two-year period beginning 1 December 2000. | 306 | 306 | 306 (Patient delay 291; Referral delay 288) | T1, T4, T7, T13 | Patient medical and/or dental records  Patient questionnaire | ICD-O  TNM Stage | Medical records and/or dental records |  |
| Vernham (1994) | Prospective cross sectional study | To investigate whether advanced stage (III & IV) patients present after longer duration of symptoms than those with early stage (I & II). | UK (Scotland) | Specialist care (single site) | All patients with newly diagnosed and histologically confirmed head and neck malignancies presenting between Jan 1990 and Dec 1991. | 127 | 127 | 127 | T2, T10, T13 | Patient records | TNM Stage | Medical records |  |
| Wildt (1995) | Prospective patient survey | To assess and describe the importance of the different elements of the delay; to investigate the possible correlation between the delay and some tumour and patient factors; and to examine whether the delay can be used as an independent prognostic indicator. | Denmark | Specialist care (multi- site) | All patients with oral squamous cell carcinoma seen between Jan 1986 to Nov 1990. | 167 | 167 | 167  (158 for histological score outcome) | T1, T5, T8 | Patient questionnaire | Tumour size TNM Stage  Histological score Corrected Survival | Medical records |  |
| **Brain/CNS (Central Nervous System)** | | | | | | | | | | | | |  |
| Balasa (2012) | Prospective cohort study | To evaluate pre-operatory myellic involvement and post-operative follow up and to establish the interval and capacity of recovery for adults with primary intramedullary tumours. | Romania | Specialist care (single site) | Adult patients diagnosed with, and operated for intramedullary tumours from January 2001 until December 2007. | 68 (all spinal cord tumours) | 14 (intra-medullary tumours) | 13 | T4 | Not stated | Neurological function. | Medical records |  |
| **Melanoma** | | | | | | | | | | | | |  |
| Baade (2006) | Telephone survey | To examine the relationship between melanoma thickness and reported time from first recognition and from first physician contact to the diagnosis of invasive melanoma. | Australia | Population-based | Patients diagnosed as having melanoma up to 4 years before the study. | 4839 | 3887 | 3772 | T1, T4, T8 | Patient interview (Computer-aided standardised telephone interview) | Breslow thickness -millimetres | Melanoma Patient Register |  |
| Carli (2003) | Consecutive patient survey | To report the pattern of detection of and variables independently associated with early diagnosis in melanoma. | Italy | Specialist care (multi- site) | Cases of cutaneous melanoma consecutively identified from January 1, 2001 to December 31, 2001. | 816 | 816 | 785 | T3, T14 | Patient questionnaire | Breslow thickness -millimetres | Medical records |  |
| Cassileth (1982) | Consecutive patient survey | To explore the correlation between time to diagnosis and thickness of lesion. | USA | Specialist care (single site) | Patients with superficial spreading melanoma diagnosed since 1976. | 245 | 245 | 238 | T4 | Routine data collection, checked by patient re-interview | Breslow thickness -millimetres | Medical records |  |
| Helsing (1997) | Consecutive patient survey | To determine correlation between delay and Breslow thickness. | Norway | Primary and specialist care (single site) | Primary cutaneous malignant melanoma patients diagnosed between February and September 1994. | 478 | 352 | 240 | T1, T9, T5 | Patient questionnaire | Breslow thickness -millimetres | Cancer registry |  |
| Krige (1991) | Consecutive patient survey | To define the extent of delay and the consequences of such delay. | South Africa | Specialist care (single site) | Patients with newly diagnosed Stage 1 malignant melanoma, referred between 1987 and 1989. | 250 | 250 | 250 | T3, T5, T14 | Patient interview | Clark level  Breslow thickness -millimetres | Medical records Patient interview |  |
| Metzger (1998) | Retrospective review of patient records | To discuss the frequency of physician’ misdiagnoses and the consequences of a delay in diagnosis. | Germany | Specialist care (single site) | Patients treated from January 1986 to March 1997 with palmoplantar and subungual melanoma. | 83 | 83 | 83 | T8 | Medical reports | Breslow -thickness millimetres | Medical records |  |
| Montella (2002) | Consecutive patient survey | To test the relationship between diagnosis/treatment delay and some clinical variables. | Italy | Specialist care (single site) | Patients who had surgery for histologically confirmed melanoma between January 1996 and December 2000. | 530 | 530 | 472 | T3, T5, T14 | Patient interview | Breslow thickness -millimetres | Medical records |  |
| Richard (1999) | Consecutive patient survey | To assess the correlation between the delay in diagnosis and the Breslow thickness. | France | Specialist care (multi-site) | Patients recently diagnosed with malignant melanoma from January 1, 1995 to 31 July, 1996. | 645 | 590 | 418 | T3, T5, T13, T15 | Patient records  Patient interview | Breslow thickness -millimetres | Medical records |  |
| Schmid-Wendtner (2002) | Consecutive patient survey | To assess the extent of delay in the diagnosis and treatment of cutaneous melanoma and the consequences of such a delay and the associated factors. | Germany | Specialist care (single site) | Patients treated for histologically proven primary cutaneous melanoma between January 1999 and January 2001. | 233 | 233 | 233 | T3, T14 | Patient interview | Breslow thickness -millimetres | Medical records |  |
| Temoshok (1984) | Consecutive patient survey | To investigate the relationship between patient delay in seeking medical attention and prognostic indicators, tumour characteristics, and demographic and behavioural factors. | USA | Specialist care (multi-site) | Patients who had biopsy-confirmed diagnoses of malignant melanoma. | 106 | 106 | 106 | T3 | Patient interview | Clark level  Breslow thickness -millimetres | Medical records |  |
| Tørring (2013) | Prospective cohort study | To assess the association between the length of the diagnostic interval and the ﬁve-year mortality for the ﬁve most common cancers in Denmark while addressing the above methodological and analytical issues. | Denmark | Population based | All patients with newly diagnosed colorectal, lung, melanoma skin, breast or prostate cancer above the age of 17 in the former Danish County of Aarhus during 1 year (inclusion period from 1 September 2004 to 31 August 2005), which was equivalent to 56% of all new cancers in Denmark during that year. | 1543 | 1295 | 1128: Melanoma=116 (10%) | T8 | Cancer registry  GP Questionnaire | Survival | Registry data Danish Cancer Registry; County Hospital Discharge Registry  Danish Civil Registration System,  GP Questionnaire |  |
| **Non-melanoma skin** | | | | | | | | | | | | |  |
| Alam (2011) | Prospective cohort study | To clarify the reasons for delay in the presentation for diagnosis and treatment of non-melanoma skin cancer. | USA | Specialist care (single site) | Eligible subjects were consecutive patients presenting for Mohs micrographic surgery for NMSC between March and December 2005. | 982 | 860 | 823 | T3 | Patient questionnaire | Increase in tumour size | Medical records  Patient questionnaire |  |
| Renzi (2010) | Retrospective review of patient records Patient interview | To evaluate factors associated with SCC size, including diagnostic/treatment delay and patient and tumour characteristics. | Italy | Specialist care (single site) | Patients with SCC treated between June 2004 and February 2006 at our hospital, a dermatologic referral centre for central and southern Italy. | 2179 | 1895 | 308 | T1, T9 | Patient records Patient interview | Size of squamous cell carcinoma | Medical records |  |
| Tokuda (2009) | Retrospective review of patient records | To investigate relative values of symptom-to-visit intervals in patients with cancer and to classify them into groups with homogenous intervals and to examine the relation of the intervals to distant metastasis in patients with common types of solid tumours. | Japan | Specialist care (single site) | All patients with a diagnosis of cancer registered in the hospital cancer registry database for 10 years from January 1991 through December 2000. | Not reported | 123 | 123 | T1 | Cancer registry, patient records | Metastasis No metastasis | Medical records |  |
| **CTYA (Children, Teenagers and Young Adults)** | | | | | | | | | | | | |  |
| **Brain/CNS(Central Nervous System)** | | | | | | | | | | | | |  |
| Brasme (2012) | Retrospective review of patient records Population based cohort study | To analyse, in a paediatric population-based study, the consequences of the time to diagnosis of medulloblastoma on initial tumour stage, survival, and neuropsychological and neurological outcome, while taking confounding factors into account. | France | Population based | All patients in one French region (Ile-de-France, the Paris metropolitan region) who were younger than 15 years when diagnosed with a histologically-confirmed medulloblastoma from 1990 through 2005. | 170 | 166 | 166: survival; 96 (survivors): neurological disability. | T4 | Patient records Cancer registry | Stage  Chang-Harisiadis classification Survival Tumour volume; neurological disability; IQ score | Medical records |  |
| Crawford (2009) | Retrospective review of patient records | To determine the relationship between clinical presentation, radiographic features, pathology and treatment on overall survival of newly diagnosed paediatric primary spinal cord tumours. | USA | Specialist care (single  site) | All patients identified with primary spinal cord tumours diagnosed between 1995 and present (2008). | 25 | 25 | 25 | T4 | Patient records | Grading based on WHO criteria Survival | Medical records |  |
| Halperin (2001) | Retrospective review of patient records | To test the hypothesis that children with a longer duration of symptoms prior to diagnosis of medulloblastoma have more advanced disease. | USA | Specialist care (single  site) | Selected cases with histologically confirmed medulloblastoma who were evaluated from January 1, 1974 to October 31, 1999 (with the addition of patients who were seen initially at associated community hospitals). | 116 | 108 | 108 | T4 | Patient records | Tumour stage Chang-Harisiadis classification | Medical records |  |
| Kameda-Smith (2013) | Retrospective review of patient records | The objectives were to (a) identify the common presenting symptoms of posterior fossa tumours, (b) determine the time interval from the first attributable symptom to the radiological diagnosis of a posterior fossa tumour, (c) compare the West of Scotland with other international centres, and (d) identify which factors correlate with outcome for these children. | UK (Scotland) | Specialist care (single site) | Children diagnosed with posterior fossa tumours from January 2000 to September 2011 in a large West of Scotland Neurosurgical Centre. | 69 | 66 | 66 | T4 | Patient records triangulated with data from the HISS (Hospital Information Support Systems), PACS (Picture Archive and Communication Systems) and Clinical Portal. | Survival | Medical records |  |
| Kukal (2009) | Retrospective review of patient records | To study the hypothesis that a delay in the diagnosis of paediatric brain tumours results in decreased survival outcome probability. | Switzerland | Specialist care (single site) | Children up to the age of 16 years with primary brain tumours, admitted consecutively to the hospital from January 1980 to December 2004. | 315 | 315 | 315 (T4) 234 (T8) | T4, T1, T8 | Patient records | Survival (overall progression free) | Medical records |  |
| Sethi (2013) | Retrospective review of patient records | To raise awareness of the variety of symptom complexes related to specific central nervous system (CNS) germ cell tumours (GCTs), and to assess the impact of delayed diagnosis on outcomes. | USA | Specialist care (single site) | Patients treated for intracranial pure germinoma and nongerminitomous GCTs at Massachusetts General Hospital between 1998 and 2012. | 70 | 70 | 70 | T4 | Patient records | Stage  Disseminated disease Survival | Medical records |  |
| **Head and Neck** | | | | | | | | | | | | |  |
| Butros (2002) | Retrospective review of patient records | To assess the degree, cause and consequence of delays from presenting signs to diagnosis of retinoblastoma. | USA | Specialist care (single site) | Consecutive patients who presented with newly diagnosed retinoblastoma between Nov 1993 and Jan 1998. | 64 | 64 | 57 | T1, T6 | Parent or primary care giver interviews | Choroidal extension Metastatic disease Enucleation versus eye salvage Survival | Medical records |  |
| Erwenne (1989) | Retrospective review of patient records | To analyse the importance of pre-treatment, time-dependent factors associated with lateness of referral of cases that could be explanatory of the risk of extraocular disease and of poor survival. | Brazil | Specialist care (single site) | Consecutive patients of histopathologically confirmed retinoblastoma admitted between Jan 1975 and Dec 1985. | 161 | 158 | 158(stage) 153(survival) | T5 | Patient records | Stage  Intra vs extra ocular disease Survival (3-year) | Medical records |  |
| Goddard (1999) | Retrospective parental survey | To establish the extent of diagnostic delay in retinoblastoma, to ascertain whether any risk factors were associated with delayed diagnosis, and to examine whether or not delay in diagnosis altered treatment outcome. | UK  (England) | Specialist care (single site) | Patients with retinoblastoma treated between Jan 1993 and Dec 1996. | 112 | 100 | 100 | T1, T7, T2 | Parent interviews (Face-to-face and telephone)  Patient records | Type of treatment received (No further information provided) | Medical records |  |
| Wallach (2006) | Retrospective review of patient records | To determine how time to diagnosis of retinoblastoma has evolved over a 40-year period in Switzerland. | Switzerland | Specialist care (multi- site) | Swiss patients diagnosed with retinoblastoma and treated between 1963 and 2004. | 139 | 139 | 139 | T4 | Patient records | Tumour stage  Murphree group E classification | Medical records  Cancer Registry  Oncologists |  |
| **Leukaemia** | | | | | | | | | | | | |  |
| Lins (2012) | Retrospective review of patient records | To describe the interval between symptom onset and diagnosis of acute leukaemia; secondly, to assess the risk factors for possible delayed diagnosis; and lastly to investigate the eﬀect of delayed diagnosis on early morbid-mortality and EFS. | Brazil | Specialist care (single site) | Patients aged 1 month through 18 years with acute leukaemia were analysed and admitted at the Paediatric Oncology Unit at IMIP between January 1, 2000, and December 31, 2004. | Not explicitly reported but does say two were excluded, so could infer 290 | 288 | 288 | T4 | Patient records | Survival (event-free) Early death  (< 30 days from diagnosis). | Medical records |  |
| Marwaha (2010a) | Retrospective review of patient records | To identify acute lymphoblastic leukaemia (ALL) patients with initial diagnosis of juvenile rheumatoid arthritis (JRA), compare their clinic laboratory characteristics and outcome with other ALL patients treated at our centre. | India | Specialist care (single site) | Children with acute lymphoblastic leukaemia (ALL) registered in the Paediatric Oncology Clinic of the Advanced Paediatric Centre of PGIMER between January 1990 to December 2006. | 762 | 762 | 762 | T13 | Patient records | Survival | Medical records |  |
| Marwaha (2010b) | Retrospective review of patient records | To determine the pattern of deaths in children with acute lymphoblastic leukaemia (ALL) and identify the problem areas in management. | India | Specialist care (single site) | All children with acute lymphoblastic leukaemia in the period between January 1990 and December 2006. For purpose of analysis, the period of accrual was divided into 2 eras: January 1990-December 1997 and January 1998-December 2006. | 532 | 316 | 316 | T4 | Patient records | Survival: event free; overall | Medical records |  |
| Wahl (2012) | Retrospective review of patient records | To evaluate the effect of treatment delay from time of hospital admission on the risk of relapse, death, intensive care unit (ICU) transfers, and episodes of bacteremia in children with newly diagnosed acute lymphoblastic leukaemia (ALL). | USA | Specialist care (single site) | All patients consecutively diagnosed with ALL at Children’s Hospital & Research Centre Oakland from 1995 to 2007. | 207 | 207 | 207 | T15 | Patient records | Survival Relapse rate; bacteremia; admission to ICU. | Medical records |  |
| **Connective tissue** | | | | | | | | | | | | |  |
| Bacci (1999) | Prospective cohort study | To verify the assumption that delayed diagnosis of Ewing's sarcoma may affect the stage of the disease and, in turn, that it may negatively influence prognosis. | Italy | Specialist care (single site) | Consecutive series of newly diagnosed cases of Ewing's sarcoma seen between 1979 and 1997. | 618 | 618 | 618 | T4 | Patient records | Stage:  Localised vs Metastatic | Medical records |  |
| Ferrari (2010) | Retrospective review of patient records | To investigate how symptom interval correlates with tumour stage or other variables of known prognostic value, and with disease outcome. | Italy | Specialist care (single site) | Consecutive cases of previously untreated patients up to 21 years of age with a histological diagnosis of soft tissue sarcomas, who were treated at the unit between 1977 and 2005 and whose data were recorded in the database. | 653 | 575 | 575 | T4 | Patient records | Survival: 5 year event free | Medical records |  |
| Simpson (2005) | Retrospective review of patient records  (Tumour registry) | To analyse the presenting features, Enneking stage, patient and doctor delay, and size of primary tumour to observe the effects on local recurrence, metastasis and survival. | UK (Scotland) | Population-based | Patients with Ewing's sarcoma of the upper extremities identified from the Scottish Bone Tumour Registry in the 40-year period up to 2005. | 19 | 19 | 19 | T1, T8 | Tumour registry | Enneking stage | Medical records/Tumour registry |  |
| Yang (2009) | Retrospective review of patient records | To investigate the presenting patterns of osteosarcoma in Hong Kong children and its management and evaluate any possible corresponding impact on disease outcomes. | Hong Kong | Specialist care (single site) | Children diagnosed to have osteosarcoma who received treatment between 1 March 1994 and 31 October 2005. | 51 | 51 | 51 | T1, T8 | Patient records | Metastases/  no metastases  Chemonecrosis factor >90% Limb salvage surgery Relapse Death | Medical records |  |
| **Solid tumours** | | | | | | | | | | | | |  |
| Loh (2012) | Cancer registry | To identify factors associated with diagnostic delay (time between symptom onset and diagnosis) in paediatric tumours in a population-based study, and to assess the impact of delay on subsequent outcome. | China | Specialist care (multi-site) | All children aged 18 and below with a newly diagnosed solid tumour reported to the Singapore Childhood Cancer Registry between September 1997 and December 2007. | 712 | 390 | 390 | T1, T4, T8 | Patient records Cancer registry Emergency room charts and referral letters from the hospitals. Children's Cancer Foundation. | Stage Survival | Registry data  (The Singapore Childhood Cancer Registry) |  |
| **Leukaemia** | | | | | | | | | | | | |  |
| Bertoli (2013) | Retrospective review of patient records | To assess the effect of time from diagnosis to treatment (TDT) on overall survival, early death, and complete response in a retrospective cohort of patients with acute myloid leukaemia treated with intensive chemotherapy between 2000 and 2009. | France | Specialist care (single site) | All consecutive patients with AML (excepting acute promyelocytic leukaemia) were registered. | 1117 | 599 | 599 | T15 | Patient records | Survival | Medical records |  |
| Friese (2011) | Cancer registry | To estimate the time between chronic lymphoctic leukaemia (CLL) associated signs and symptoms and diagnosis and to assess several covariates of delay. Also to evaluate the effect that delays in diagnosis and receipt of flow cytometry might have on survival. | USA | Population based | Patients in the participating registries who were diagnosed with CLL between 1992 and 2002 and who met eligibility criteria. | 5831 | 5831 | 5086 : survival ; 4081: delays | T4 | Cancer registry (SEER Medicare dataset) | Survival | Registry data (SEER Medicare database) |  |
| Prabhu (1986) | Retrospective review of patient records | To study the prognostic value of certain factors in relation to survival in patients with chronic myeloid leukaemia (CML). | India | Specialist care (single-site) | All cases of chronic myeloid leukaemia (CML) seen between September 1975 and March 1983. | 239 | 117 | 117 | T4 | Patient records | Survival | Medical records |  |
| **Lymphoma** | | | | | | | | | | | | |  |
| Foulc (2003) | Retrospective review of patient records | To determine the prognostic factors associated with survival in patients with Sezary's Syndrome (epidermotropic cutaneous T-cell lymphoma). | France | Specialist care (single site) | Selected patients diagnosed with Sezary's Syndrome between January 1989 and May 2000. | 30 | 28 | 28 | T4 | Patient records | Survival | Medical records |  |
| Jacobi (2008) | Retrospective review of patient records | To test whether the prognosis of patients with follicular lymphoma is dependent on the length of symptoms before diagnosis and whether the course of the disease is determined by how long the 'Watch and Wait' approach lasts. | USA | Hospital Cancer Registry | Patients diagnosed between 1992 and 2002 selected from the Minnesota Cancer Surveillance System Database. | 130 | 77 | 77 | T4 | Patient records | Survival | Medical records |  |
| Kim (1995) | Retrospective review of patient records | To identify the important prognostic factors in a large group of patients with erythrodermic mycosis fungoides (MF) and Sezary Syndrome (SS) treated with traditional therapies. | USA | Specialist care (single site) | Selected patients classified as having erythrodermic (T4) disease and who were treated between 1958 and 1993. | 106 | 106 | 106 | T4 | Patient records | Survival | Medical records |  |
| Maguire (1994) | Retrospective review of patient records | To evaluate the risk function of the duration of symptoms upon survival after diagnosis, whilst taking into account the effects of age, sex, tumour site and tumour stage at diagnosis. | Spain | Specialist care (single site) | All patients registered with the Tumour Registry of the Hospital Del Mar between 1978 and 1989 with a diagnosis of lymphoma cancer. | 150 | 150 | 150 | T4 | Patient records  (Tumour registry) | Survival  Stage Local vs Regional vs Disseminated | Medical records |  |
| Norum (1995) | Retrospective review of patient records | To clarify the impact of delay as a prognostic factor in Hodgkin's disease. | Norway | Specialist care (single site) | All patients treated for Hodgkin's Lymphoma between January 1985 and June 1993. | 50 | 50 | 50 | T4 | Patient records | Survival  Clinical stage Ann Arbor classification | Medical records |  |
| **Myeloma** | | | | | | | | | | | | |  |
| Friese (2009) | Retrospective review of SEER-Medicare database | To identify the predictors of diagnostic delay and associated complications in patients with multiple myeloma. | USA | SEER-Medicare database | Patients diagnosed with multiple myeloma between 1 February 1992 and 31 December 2002. | 8735 | 5406 | 5406 | T4 | SEER-Medicare database | Complications  Renal failure  /dysfunction or skeletal | Medical records |  |
| Kariyawasan (2007) | Retrospective review of patient records | To analyse the causes and consequences of a delay in diagnosis of myeloma. | UK  (England) | Specialist care (single site) | All patients attending the hospital myeloma clinic between 2001 and 2006. | 103 | 92 | 92 | T4 | Patient records | Survival  Durie-Salmon stage level | Medical records |  |
| **Connective tissue** | | | | | | | | | | | | |  |
| Bacci (2002) | Prospective cohort study | To investigate whether there is a relationship between the stage of disease at presentation and several clinical and pathological characteristics, including the interval between the onset of first symptoms or signs and the final diagnosis. | Italy | Specialist care (multi-site) | All patients with primary high-grade central osteosarcoma of the extremities seen at the Rizzoli Institute between January 1980 and December 1999 and the Department of Orthopaedic Oncology between January 1995 and December 1999. | 1090 | 1071 | 1071 | T4 | Patient records | Stage at presentation:  Localised vs Metastatic | Medical records |  |
| Nakamura (2011) | Retrospective review of patient records | To investigate whether symptom-treatment delay are associated with the presence of metastasis at diagnosis, overall survival, and distant metastasis free survival in primary soft tissue sarcomas. | Japan | Specialist care (single site) | 100 newly diagnosed patients with primary soft tissue sarcoma referred to the hospital between January 2001 and December 2009. | 100 | 100 | 82 | T1, T4 | Patient records | Survival | Medical records |  |
| Rougraff (2007) | Prospective cohort study | To assess whether patient's duration of symptom before diagnosis correlated with survival (overall and disease-free), size of the tumour at diagnosis, and presence of metastatic disease. | USA | Specialist care (single  site) | A consecutive series of patients referred to the hospital between 1992 to 2003 for extremity or flank sarcoma. | 624 | 624 | 624 | T4 | Patient interview | Survival  Tumour size at diagnosis | Medical records |  |
| Ruka (1988) | Retrospective review of patient records | To determine whether a relationship between duration of symptoms and size of the sarcoma with high-grade malignancy in the absence of distant metastases might exist before definitive surgery, and whether this interdependence can provide additional predictive information to other prognostic factors. | Poland | Specialist care (single site) | All patients with high-grade soft tissue sarcoma (STS) who underwent curative resection between January 1950 and December 1984. | 285 | 266 | 266 | T5 | Patient records | Survival  Tumour size | Medical records |  |
| Saithna (2008) | Prospective analysis of collected patient data | To assess whether symptom duration has any impact on patient survival, and also whether or not patient and tumour related factors are related to the duration of symptoms prior to presentation. | UK  (England) | Specialist care (single site) | All patients diagnosed or treated with a soft tissue sarcoma over a 25 year period. | 1508 | 1508 | 1508 | T4 | Patient records | Tumour grade: High  Intermediate  Low  Tumour depth: Subcutaneous Deep  Disease spread: Metastatic Non-metastatic | Medical records |  |
| Wurtz (1999) | Retrospective review of patient records | To determine the duration of delay before the diagnosis of primary bone sarcoma of the pelvis and their effect with respect to survival. | USA | Specialist care (single site) | A consecutive series of patients referred for primary bone sarcoma of the pelvis from 1975 to 1995. | 70 | 68 | 68 | T4 | Patient records | Survival  TNM Stage and Enneking Stage | Medical records |  |
| **Carcinoid** | | | | | | | | | | | | |  |
| Toth-Fegel (2004) | Retrospective review of patient records and patient interviews | To determine whether there are significant correlations between delay of diagnosis of abdominal carcinoid tumours the extent of disease at diagnosis, between extent of disease and survival, and between delay of diagnosis and survival. | USA | Specialist care | Patients with abdominal carcinoid tumours proved by histopathology and abnormally elevated levels of 5-hydroxyindolacetic acid in 24 hour urine collections. | 115 | 115 | 115 | T4 | Patient records Patient interviews | Primary tumour only; presence of lymph node metastases, presence of liver metastases; presence of carcinomatosis; presence of extra-abdominal metastases. Survival | Medical records |  |
| **Thyroid** | | | | | | | | | | | | |  |
| Tokuda (2009) | Retrospective review of patient records | To investigate relative values of symptom-to-visit intervals in patients with cancer and to classify them into groups with homogenous intervals and to examine the relation of the intervals to distant metastasis in patients with common types of solid tumours. | Japan | Specialist care (single site) | All patients with a diagnosis of cancer registered in the hospital cancer registry database for 10 years from January 1991 through December 2000. | Not reported | 99 | 99 | T1 | Cancer registry, patient records | Metastasis No metastasis | Medical records |  |
| **Multisite** | | | | | | | | | | | | |  |
| Tørring (2013) | Prospective cohort study | To assess the association between the length of the diagnostic interval and the ﬁve-year mortality for the ﬁve most common cancers in Denmark while addressing the above methodological and analytical issues. | Denmark | Population based | All patients with newly diagnosed colorectal, lung, melanoma skin, breast or prostate cancer above the age of 17 in the former Danish County of Aarhus during 1 year (inclusion period from 1 September 2004 to 31 August 2005). | 1543 | 1295 | 1128 | T8 | Cancer registry  GP Questionnaire | Survival | Registry data Danish Cancer Registry; County Hospital Discharge Registry  Danish Civil Registration System,  GP Questionnaire |  |
