# Supplementary material for: Is increased time to diagnosis and treatment in symptomatic cancer associated with poorer outcomes? Systematic review
Source: Br J Cancer. 2015 Mar 3;112(Suppl 1):S92–S107. doi: 10.1038/bjc.2015.48 (PMC4385982; doi:10.1038/bjc.2015.48)
Supplement: Supplementary Information [file bjc201548x3.docx]

**Supplementary Online Material – Summary of Outcomes**

| **Study Identifier** | | **Measure of Association** | **Main Results** | **Statistical Significance** | **CI Lower** | **CI Upper** | **Reported Interpretation** | **Sub-group analyses** |
| --- | --- | --- | --- | --- | --- | --- | --- | --- |
| **Breast** | | | | | | | | |
| Brazda (2010) | | Kaplan-Meier (survival). Fishers exact test and chi-square analysis. | Survival and interval to treatment of:  0-45 days, 46-90 days, >90 days  Time to treatment:  <90 and >90 days | Non-significant. |  |  | There was no association between the interval to treatment after a diagnosis of breast cancer and survival. | Not reported |
| Eastman (2013) | | Kaplan-Meier, Chi square analysis and Cox regression. | Survival:  Time to treatment:  0-45 days, 46-90 days, >90 days  Time to treatment:  <90 and >90 days  LRR:  Time to treatment and LRR vs no LRR:  38 ± 6 vs 44 ± 2; mean ± SEM.  Cox regression. | p=0.25  p=0.06  p=0.37  p=0.5 |  |  | No association between the interval to treatment and survival was seen.  There was no significant difference in time to treatment for those patients with a LRR compared to those without a recurrence. In addition, LRR and time to treatment were evaluated in a Cox regression model, and there was no increased risk of recurrence in patients with increased interval to treatment. | Not reported |
| Ermiah (2012) | | Chi-square, Fisher's exact test. | Diagnosis delay of < 3 months/3-6 months /> 6 months and:  Stage 1 (n=18): 94.4%/ 5.6%/ 0.0%  Stage 2 (n=51): 74.5%/ 23.5% 2.0%.  Stage 3 (n=108):2.8%/ 10.2%/ 87.0%.  Stage 4 (n=23):0.0%/ 0.0%/ 100.0%.  Early stages (n= 69): 79.75%/ 18.8%/ 1.4%.  Late stages (n= 131): 2.3%/ 8.4%/ 89.3%.  T 1 (n=25): 96.0%/ 4.0%/ 0.0%.  T 2 (n=54): 60.8%/ 33.3%/ 5.9%.  T 3 (n=78): 4.3%/ 25.7%/ 70.0%.  T 4 (n=43): 0.0%/ 4.8%/ 95.2%.  N 0 (n=50): 80.0%/ 18.0%/ 2.0%.  N 1 (n=76): 22.4%/ 31.6%/ 46.1%.  N 2 (n=58): 1.7%/ 12.1%/ 86.2%.  N 3 (n=16): 0.0%/ 0.0%/ 100.0%.  M 0 (n=177): 32.8%/ 21.5%/ 45.8%.  M 1 (n=23): 0.0%/ 8.7%/ 91.3%. | p<0.0001.  p <0.0001.  p <0.0001.  p <0.0001.  p <0.0001. |  |  | Diagnosis delay was significantly associated with large tumour size and with positive lymph nodes. 23 patients presented with metastasis at time of diagnosis, 91.3% of those had diagnosis delay >6 months after symptoms. Late clinical stage of breast cancer was found in 65.5% of all patients and it tended to be more frequent among women with diagnosis delay >6 months than among women who had diagnosed < 3 months after onset of symptoms. | Not reported |
| McLaughlin (2012) | | Chi-square test, Cox proportional hazards regression. | Diagnosis to treatment of ≥ 60 days vs 0 to 59 days and breast cancer specific survival:  Early stage: (n=1065), HR 0.58.  Late stage: (n=721), HR 1.42.  Diagnosis to treatment of ≥ 60 days vs 0 to 59 days and overall survival:  Early stage (n=1065), HR 0.72.  Late stage: (n=721), HR 1.28. | P=0.46  P=0.22  P=0.40  P=0.32 | 0.14  0.81  0.33  0.79 | 2.45  2.48  1.55  2.09 | Crude results showed no effect of Dx2Tx length on breast cancer-specific or overall survival. Adjusted results showed that the relationship between Dx2Tx length and both overall and breast cancer-specific survival was modified by stage at diagnosis. | Not reported |
| Mujar (2013) | | Kaplan-Meier, Cox regression, adjusted for ethnicity and stage. | Time to primary treatment of:  ≤ 2 weeks and > 2 weeks: 281(43.3%) HR 1.000 and 367(56.6%) HR 0.810  ≤ 1 month and > 1 month: 506 (78.1%) HR 1.000 and 142(21.9%) HR 1.238  ≤ 2 months and > 2 months: 602(92.9%)  HR 1.000 and 46(7.1%) HR 1.236. | P=0.191  P=0.250  P=0.465 | 0.591  0.861  0.700 | 1.111  1.779  2.181 | Delay in TPT in days, weeks and months did not affect survival. There was also no signiﬁcant difference found in survival when using the 3 cut off points of 2 weeks, 1 month and 2 months. Therefore, time to primary treatment had no impact on breast cancer survival. | Not reported |
| Redaniel (2013) | | Relative survival expressed as percentages, estimated using Ederer II method.  Mortality: Excess hazards ratios (EHRs) at 5 years were computed using a generalised linear model with a Poisson error structure. | Association of waiting times of:  < 25 days/25-38 days/39-62 days and relative survival.  RS: 93%/ RS: 93.5%/ RS: 92.1%.  Crude Model.  Association of waiting times of:  < 25 days/25-38 days/39-62 days and excess mortality:  EHR 1.10/ EHR 1.00/ EHR 1.06. | Not reported | 92.5/92.8/90.8  0.99/-/0.88 | 993.4/94.2/93.4  1.23/-/1.27 | Relative survival estimates did not differ by waiting time categories. In comparison with patients with waiting times of 25 to 38 days, patients waiting for 25 days had a 10% higher excess mortality. However, this effect was reversed after adjusting for all covariables. | Not reported |
| Smith (2013) | | Kaplan-Meier, log rank test, Cox proportional hazards regression, Cox regression using covariates including race, insurance status, SES, age group, cancer stage, tumour markers, and tumour characteristics for association of treatment delay and survival. | 5 year survival of all patients and treatment delay (HR (95% CI):  Less than 2 weeks: HR 0.84  2-4 weeks: HR 0.83  4-6 weeks: HR 0.80  More than 6 weeks: HR 0.80 | P=0.005 | 0.83  0.81  0.77  0.74 | 0.85  0.85  0.83  0.82 | Young women with breast cancer with a longer treatment delay time have significantly decreased survival time compared with those with a shorter treatment delay time. | Not reported |
| Sue (2013) | | Kaplan-Meier, Mann-Whitney U test. Association of delay and overall and disease free survival. | Disease free survival and delay:  < 39.5 days: 86.1%  39.5 days or more: 95.2%  Overall survival and delay:  < 39.5 days: 93.0%  39.5 days or more: 97.6% | P=0.114  P=0.322 | Not reported | Not reported | We did not observe any differences in disease-free survival or overall survival when comparing patients with less than 39.5 days of delay time with those with greater than 39.5 days. | Not reported |
| Tørring (2013) | | Kaplan-Meier method, conditional logistic regression to estimate ﬁve-year mortality odds ratios as a function of the diagnostic interval using restricted cubic splines and adjusting for comorbidity, age, sex and type of cancer. | Mortality rates (MR %) and crude/adjusted odds ratios (OR) for the 1st and 4th compared with 2nd and 3rd diagnostic interval quartiles (DIQ) for patients with a) alarm or serious symptoms and b) vague symptoms.  Alarm or serious symptoms  DIQ MR% Crude OR Adjusted OR  1^st^ 20.6 1.02 1.05  2^nd^ + 3^rd^ 20.3 1 1  4^th^  25.0 1.31 1.18  Vague symptoms  DIQ MR% Crude OR Adjusted OR  1^st^ 8.3 Comparison unjustified  2^nd^ + ^3rd^ 28.6  4^th^ 9.1 |  | 0.48/0.47  Reference  0.63/0.52 | 2.15/2.35  Reference  2.70/2.66 | In patients with alarm or any serious symptoms, mortality grew the longer the diagnostic intervals in those 40% from this group who experienced the longest delays. In patients with alarm or any serious symptoms, having very short diagnostic intervals was also associated with a high mortality. Inversely, in patients presenting with vague symptoms, we saw much longer diagnostic intervals, the same survival probability and an opposite, concave trend between the length of the diagnostic interval and mortality. However, the latter association was not statistically signiﬁcant and the association did not apply to prostate cancer. The study displays the immense complexity and difficulty of diagnosing cancer. Yet, we can infer from the increasing trends in mortality that a few weeks can make a difference – that time matters. | Not reported |
| Wagner (2011) | | Univariate and multivariate generalized linear models for an unbalanced analysis of variance. Effect of treatment delay on tumour size. | Effect of treatment delay on tumour size: Univariate analysis.  Multivariate Logistic Regression to predict nodal status: Months from imaging to surgery, OR 1.31. | P=0.0110  P=0.1065 | Not reported  0.95 | Not reported  1.82 | In both univariate and multivariate models, no significant association was detected between time from first imaging until surgery and the change in tumour size according to the mammogram and ultrasound. In univariate analysis, the delay between imaging at our institution and surgery was significantly associated with nodal metastasis. In multivariate analysis, however, time to surgery was not significantly associated with nodal status after we controlled for patient age; tumour grade and histologic type; ER, PR, and HER2 status; lymphovascular invasion; and baseline imaging size. | For the 635 patients who underwent imaging at M.D. Anderson Cancer Centre, a sub group analysis was performed of those with a delay over the median of 21 days, those with a delay over 35 days (3rd quartile), 49 days (top 10%), and 59 days (top 5%). No association between the delay from M. D. Anderson imaging until surgery and change in tumour size was found for any of these subgroups. |
| Warner (2012) | | Multinomial logistic regression models.  Time to diagnosis and risk of advanced stage breast cancer. | Odds ratios for time to diagnosis and risk of advanced-stage breast cancer  Stage II vs. Stage I (n = 5840):  0-30 (days) OR 1.00  30-59 days OR 0.95  60-89 days OR 0.90  90-179 days OR 1.06  ≥180 days OR 1.01.  Stage III/IV vs. Stage I (n = 2872):  0-30 (days) OR 1.00  30-59 days OR 1.02  60-89 days OR 1.22  90-179 days OR 1.62  ≥180 days OR 1.92. |  | Ref  0.85  0.77  0.92  0.88  Ref  0.89  1.03  1.37  1.63 | Ref  1.06  1.04  1.22  1.17  Ref  1.17  1.46  1.91  2.26 | Among symptomatic women, time to diagnosis ≥60 days was associated with increased risk of stage III/IV diagnosis. Women with 60-89 days between first symptom and diagnosis were 22 % more likely than women with 0-30 days to have stage III/IV breast cancer, and those with ≥180 days were 92 % more likely. | Not reported. |
| Wright (2010) | | Pearson's Chi Square test and multinominal logistic regression. | Stage and treatment delay ≤ 30 days/> 30 days  Stage I: 4103 (77.0%)/1223 (23%)  Stage II: 7944 (75.2%)/2616 (24.8%)  Stage III: 3017 (75.2%)/993 (24.8%)  T stage and ≤ 90 days/> 90 days  Stage I: 5106 (95.9%)/220 (4.1%)  Stage II: 10,073 (95.4%)/487 (4.6%)  Stage III: 3822 (95.3%)/188 (4.7%)  T Stage and delay ≤ 30 days/> 30 days  T1: 7276 (75.4%)/2372 (24.6%)  T2: 6564 (74.0%)/2017 (23.5%)  T3: 868 (74.0%)/305 (26.0%)  T4: 329 (73.6%)/118 (26.4%)  T stage and delay ≤ 90 days/> 90 days  T1: 9201 (95.4.0%)/447 (4.6%)  T2: 8222 (95.8%)/359 (4.2%)  T3: 1115 (95.1%)/58 (4.9%)  T4: 422 (94.4%)/25 (5.6%)  Multivariate analyses: >30 days OR/>90 days OR  Stage I:1/1  Stage II: 1.14/1.17  Stage III: 0.97/0.97  Multivariate analyses: >30 days OR/>90 days OR T Stage T1:1/1  T2: 0.82/0.75  T3: 0.87/0.71  T4: 0.95/0.95 | P=0.03  P=0.31  P=0.007  P=0.03 | 1.03/1.27  0.85/0.73  0.76/0.62  0.73/0.50  0.73/0.57 | 0.94/1.45  1.12/1.28  0.90/0.91  1.04/1.03  1.24/1.59 | Smaller tumour size was associated with longer delays. | Not reported |
| Yun (2012) | | X2 test  Cox proportional hazards modelling   | Surgical treatment delay of > 31 days versus ≥ 31 days and five-year survival  Unadjusted HR 1.57  Adjusted HR 1.59 | Not reported | 1.35  1.37 | 1.81  1.84 | Among surgery patients, treatment delays of > one month were associated with worse survival for patients with rectal, pancreatic, lung or breast cancer than treatments delays of < one month.  Adjusted HR for age, sex, Charlson score, hospital type, insurance, radiotherapy, chemotherapy, type of medical care institution, year of diagnosis and waiting time. | Not reported |
| **Lung** | | | | | | | | |
| Annakkaya (2007) | | Comparison of survival in those with symptom to doctor interval ≤ 45days vs >45 days (Kaplan-Meier, log-rank):  All patients NSCLC SCLC Comparison of survival in those with symptom to treatment interval ≤ 60 days vs >60 days (Kaplan-Meier, log-rank):  All patients NSCLC SCLC  Comparison of survival in those with symptom to treatment interval ≤ 90 days vs >90 days (Kaplan-Meier, log-rank):  All patients NSCLC SCLC  TNM (Mann-Whitney U test)  All other data for association of stage or histological type of cancer with diagnostic interval is in the column 'Sub-group analyses' |  | p=0.516 p=0.525 p=0.561    p=0.015 p=0.032 p=0.765    p=0.240 p=0.526 p=0.862   Non-significant | n/a | n/a | No significant difference in survival was detected between lung cancer patients with an STI shorter than 45 days; patients with an STI longer than 60 days had significantly increased survival; patients with shorter diagnostic intervals also had a shorter median survival.   No significant difference was found when the symptom to treatment interval was correlated with stage.  Median survival was shorter in patients with a symptom to treatment interval of less than 60 days in both SCLC and NSCLC but was not statistically significant in SCLC. | Not undertaken  Comparison of stage (I-IV), and: Symptom to doctor interval (p=0.535) Doctor to diagnosis interval (p=0.026) Diagnosis to treatment interval (p=0.010) Symptom to treatment interval (p=0.357) (Kruskal-Wallis test)  Comparison of early stage (I-II) and advanced stage (III-IV) and: Symptom to doctor interval (p=0.191) Doctor to diagnosis interval (p=0.901) Diagnosis to treatment interval (p=0.672) Symptom to treatment interval (p=0.541) (Mann-Whitney U Test)  Comparison of patients with SCLC/NSCLC and: Symptom to doctor interval (p=0.010) Doctor to diagnosis interval (p=0.810) Diagnosis to treatment interval (p=0.251) Symptom to treatment interval (p=0.013) (Mann-Whitney U Test) |
| Brocken (2012) | | Continuous variables were compared using the Mann-Whitney-U test, categorical variables were compared using the X2 test, delay per stage was compared using the Kruskal-Wallis test. | Clinical stage and medial survival in months:  Ia - 45, Ib - 35, IIa - 32, II2 - 17, IIIa - 12, IIIb - 16, IV - 6, overall - 17.  Diagnostic delay was inversely related to clinical stage.  Therapeutic delay for higher stage patients  When survival time was analysed per delay, the Cox proportional hazard model showed no relation between any delay and survival. | P=0.008  P=0.001  Not reported | Not reported | Not reported | When all defined delays were analysed per stage, diagnostic delay was inversely related to clinical stage, while therapeutic delay showed the opposite, being longer for higher stage patients. However, when patients with a diagnostic thoracotomy (with longer diagnostic and shorter therapeutic delays) were excluded, none of the delays showed a relation with clinical stage and no specific delay was related with pathological stage.  When survival time was analysed per delay, the Cox proportional hazard model showed no relation between any delay and survival. | Not reported |
| Christensen (1997) | | Correlation of time intervals and stage I and II vs III and IV of disease  (Mann-Whitney, U-test): Symptom to operation or inoperability Contact with health system to operation or inoperability |  | p=0.037 p=0.017 | n/a | n/a | A few months delay before final treatment of NSCLC seems to have an impact on the preoperative stage of the cancer. | Not undertaken |
| Diaconescu (2011) | | Both univariate and multivariate Cox regression models were used to explore the association between survival and different patient characteristics. | Overall survival of advanced patients according to the treatment delay compared with the median wait time (50 days).  Urgent (wait time lower than median): 6.8 months.  Treatment delays beyond the median: 11.6 months | P=0.027 | Not reported | Not reported | Patients with advanced NSCLC were treated within a shorter delay than those with local and regional stages. For the latter, the relationship between outcome and treatment delay was inconclusive. With equivalent chemotherapy protocols, advanced patients treated on a more urgent basis had a worse survival. We hypothesize that urgent treatment might be a surrogate marker for higher symptom burden, and this parameter would be useful to collect in future prospective studies. | Not reported |
| Gonzalez-Barcala (2010) | The comparison between patients in each of the groups of each delay category was made using the chi-square test for qualitative variables and using analysis of variance for the continuous variables. Using a Cox regression, the influence of each of the delay categories, adjusted for age, sex, stage, histology, and comorbidity, on survival was analysed. | Multivariate analysis of total delay on survival:  Probability ratio <3 months  3-6 months  >6 months  Multivariate analysis of specialist consultation delay on survival:  Probability ratio <1 month  1-2 months  2-3 months  >3 months  Multivariate analysis of hospital delay on survival <1 month  1-2 months  2-3 months  >3 months | 1  0.940  0.706  1  0914  1.370  0.836  1  0.676  0.769  0.695 | 0  0.744  0.523  0  0.690  0.996  0.635  0  0.530  0.505  0.475 | 0  1.188  0.952  0  1.212  1.884  1.100  0  0.861  1.170  1.016 | The delay in the study is less in the more advanced stages and in the histology corresponding to small cell type. On analysing the influence of the total delay on survival, a total delay of >6 months assumes a 29% reduced risk of dying. The delay in specialist consultation does not influence survival. Hospitalisation delay showed a 32% less probability of death in the group with a delay between 1 and 2 months compared to the group with a delay of <1 month. On analysing the groups with a hospital delay >2 months, the same trend towards longer survival as in the shorter delay groups is observed, though it is not statistically significant, and probably due to small numbers of patients in longer delay groups. | Not reported |  |
| Gould (2008) | Comparison of the two groups with time to diagnosis of: >84 days with respect to: Likelihood of death Comparison of the two groups with time to treatment of: >84 days with respect to: Median survival (days)  Hazard of death with shorter time (log transformed days) to treatment | 80%/55%      142/535 HR 1.6 | p=.003  p<0.0001 | 1.3 | 1.9 | Survival time was better in patients who received less timely care | Not undertaken |  |
| Loh (2006) | Hazard ratio for those with patient delay of < 3 months versus 3-6 months, versus> 6 months  Hazard ratio for those with doctor delay of < 30 days versus 30-60 days versus > 60 days. (Kaplan-Meier, Log rank) | Not reported | 0.648  0.577 |  |  | Most patients presented and were treated in a short time and delays did not significantly affect survival. | Not undertaken |  |
| Maguire (1994) | Association between SDI (as a continuous variable/linear function) and survival:  Unadjusted (Cox proportional hazard) Adjusted by age & stage (Cox proportional hazard) Association between SDI (as a categorical variable/quintiles) and survival-Hazard ratio (unadjusted):  Lee desu test (test of differing survival by strata) Log rank Cox proportional hazards model (quintile 1 versus higher quintile)- SDI < 1  1≤SDI<2 SDI = 2 2<SDI≤4 5≤SDI Association between SDI (as a categorical variable/quintiles) and survival-Hazard ratio (Adjusted by age & stage): Cox proportional hazard model (quintile 1 versus higher quintile) SDI < 1  1≤SDI<2 SDI = 2 2<SDI≤4 5≤SDI | 0.992 0.993  1.0 1.292 1.753 1.377 2.71  1.0  1.223 1.634 1.293 1.183 | 0.458 0.512  0.001 0.004  0.082 <0.001 0.039 1.125  0.174 <0.001 0.099 0.282 | 0.971 0.971  0.968 1.310 1.016 0.936  0.915 1.220 0.953 0.871 | 1.013 1.015  1.725 2.344 1.866 1.725  1.636 2.189 1.753 1.608 | SDI showed a weak relationship with tumour stage at diagnosis and a non-linear relationship with prognosis  Most favourable survival was in patients with a SDI of less than 1 month. Patients diagnosed at 2 months of their first symptom showed a 75% worse survival | Not undertaken  Additionally, the ratio of the median SDI to the median survival was computed for each site (SDI to survival ratio, SSR). SSR can be thought of as an estimate of the relative opportunity for clinical lead-time bias.  Results continued: Comparison of median SDI in patients with local (2 months) versus regional (2 months) versus disseminated (2 months) : p=0.677 (Kruskall-Wallis test) |  |
| Mohan (2006) | Correlation of duration of symptoms and WHOQoL (Pearson Rho) Physical Psychological Social Environmental | -0.06 -0.01 +0.01 +0.13 | p=0.58 p=0.89 p=0.90 p=0.26 | -0.29  -0.24 -0.21 -0.09 | 0.16 0.21 0.24 0.35 | The WHO-QoL had no correlation with various symptoms or their duration | Not undertaken |  |
| Myrdal (2004) | Comparison of median symptom to treatment delay between patients with Stage IV versus all lower stages (descriptive) Comparison of median symptom to treatment delay between patients with Stage I-II versus IIIb (descriptive) Comparison of proportion of patients with stage I-II versus IIIA versus IIIB versus IV among those with symptom to treatment delay  Comparison in hospital delay of those who underwent surgery versus chemo/radiotherapy Comparison in hospital delay among those with stage I-II versus IIIB or IV Comparison of proportion of patients with stage I-II versus IIIA versus IIIB versus IV among those with hospital delay <1month Hazard ratio for patients with hospital delay: < 1 month vs. 1-2, vs 2-3, vs >3 (multivariate Cox model) Hazard ratio for patients with symptom to treatment delay: < 3 months vs. 3-6, vs >6 (multivariate Cox model) | Not reported  Not reported   Not reported    Not reported  Not reported  Not reported   0.87   0.79 | Not reported  Not reported   Not reported    Not reported  Not reported  Not reported | 0.75   0.61 | 1.00   0.97 | The symptom to treatment delay in patients with Stage IV tumours was significantly lower compared to patients with less advanced disease.  Patients with stage IIIb disease had a shorter delay than those with stage I-II disease.  Neither longer symptom to treatment delay nor longer hospital delay time are associated with a poorer prognosis. On the contrary, the prognosis was poorer in patients with a shorter delay.  The association between short delay and poor outcome was most pronounced in patients with advanced disease. | Not undertaken |  |
| Neal (2007) | Comparison of survival rates between urgent referrals and those diagnosed through other routes (log-rank test) (n=303) Comparison of proportion in T-stage among those diagnosed via urgent referral vs other routes (chi-square test) Comparison of extent of cancer among those diagnosed via urgent referral vs other routes (chi-square test) Comparison of TNM stage among those diagnosed via urgent referral vs other routes (chi-square test) * Median duration (GP referral to diagnosis) for the Urgent referral= 28 days vs 25 days for the other routes | 5.40   11.97   8.52   11.79 | p=0.020 (df=1)   p=0.035 (df=5)   p=0.004 (df=1)  p=0.038 (df=5) |  |  | There was a difference between urgent guideline referrals and other routes and TNM stage. Urgent guideline referrals had more advanced stage at diagnosis. Although there were smaller numbers in sub groups, trends were seen between referral source. There was a difference between urgent guideline referrals and other routes with respect to survival with urgent referrals having shorter survival times. | Not undertaken |  |
| Murai (2012) | Linear single regression analysis, Fisher's exact test, Mann-Whitney U test. | Not reported |  |  |  | T-stage progression was exclusively seen in patients with a wait time >4 weeks. | Not reported |  |
| Pita Fernandez (2003) | Correlation between SDI and stage Hazard ratio for those with delay lower than the median versus higher than the median (Median 2.1 months)  Univariate all patients (Log rank) - Univariate stratified by stage (Log rank) -  Multivariate (Cox model-age, sex, degree of invasion) | Not reported    Not reported Not reported 1.01 | Non-significant    p=0.34 Non-significant for all stages at p<0.05 | 0.94 | 1.08 | In univariate analysis, SDI was independent of the stage. Survival was not affected by severity of delay. | Not undertaken |  |
| Radzikowska (2012) | Survival and patient and doctor delay.  Univariate and multivariate analysis by Cox proportional hazards ratio model and log-rank test. | Univariate  No patient delay: HR=0.81  No doctor delay: HR=1.18  Multivariate  No patient delay: HR=0.88  No doctor delay: HR=1.14 | Not reported | Not reported | Not reported | Lack of patient's delay had a positive impact on survival, but lack of doctor's delay had a negative impact on survival. Multivariate analysis of survival revealed that lack of doctor's delay was a negative prognostic factor but lack of patient's delay was a positive prognostic factor. | Not reported |  |
| Salomaa (2005) | Correlation between median delay time (days) and stage (Spearman correlation coefficient) Stage I to IIIa - 13 Stage IIIb to IV - 22 Hazard ratio for patients with post presentation time (to treatment) with delay longer than or equal to the median versus patients with delay shorter than the median (Log rank test)* *Analysis also done for symptom duration (to treatment) and from first visit to GP until beginning of treatment: results similar to above (not reported in full for these intervals). | Not reported    0.60 | p = 0.0012    p = 0.02 | 0.39 | 0.91 | Patients with a longer delay are likely to have advanced stage disease. | Not undertaken |  |
| Skaug (2011) | Kaplan-Meier curves and log-rank tests, Cox proportional models and Hazard Ratios. | Diagnostic delay time and survival:  Below 1 year: N= 192; N (%)  < 2 months: 91(47)  2-5 months: 75 (39)  >5 months: 26 (14)  1 year but below 5 years: N= 56; N (%)  < 2 months: 26(46)  2-5 months: 20 (36)  >5 months: 10 (18)  5 years or more: N=23; N (%)  < 2 months: 12(52)  2-5 months: 9 (39)  >5 months: 2 (9)  Diagnostic delay time and survival: unadjusted  < 2 months: HR 1  2-5 months: HR 1.05  >5 months: HR 1.01  Diagnostic delay time and survival: adjusted  < 2 months: HR 1  2-5 months: HR 0.99  >5 months: HR 1.08 | P=0.94  P=0.94  P=0.91 | 0.80  0.70  0.74  0.72 | 1.36  1.45  1.32  1.62 | The median survival time was 5.7 months from diagnosis. The interval from symptom onset to diagnosis did not influence survival. | Not reported |  |
| Tokuda (2009) | Comparison of the mean symptom to visit intervals between patients with and without metastasis: (Student's t-test) | 53.7/47.6 | Non-significant |  |  | Delayed diagnosis is a significant positive predictor of survival but this could be partially explained by the fact that most patients who had delayed diagnosis suffered from indolent rather than the more aggressive cancers. | Not undertaken |  |
| Tørring (2013) | Kaplan-Meier method, conditional logistic regression to estimate ﬁve-year mortality odds ratios as a function of the diagnostic interval using restricted cubic splines and adjusting for comorbidity, age, sex and type of cancer. | Mortality rates (MR%) and crude/adjusted odds ratios (OR) for the 1st and 4th compared with 2nd and 3rd diagnostic interval quartiles (DIQ) for patients with a) alarm or serious symptoms and b) vague symptoms.  Alarm or serious symptoms  DIQ MR% Crude OR Adjusted OR  1^st^ 95.7 3.67 5.16  2^nd^ + 3^rd^ 85.7 1 1  4^th^  85.7 1.00 1.13  Vague symptoms  DIQ MR% Crude OR Adjusted OR  1^st^ 100.0 Comparison unjustified  2^nd^ + ^3rd^ 92.9  4^th^ 95.0 |  | 0.79/1.03  Reference  0.35/0.37 | 17.00/25.94  Reference  2.84/3.46 | In patients with alarm or any serious symptoms, mortality grew the longer the diagnostic intervals in those 40% from this group who experienced the longest delays. In patients with alarm or any serious symptoms, having very short diagnostic intervals was also associated with a high mortality. Inversely, in patients presenting with vague symptoms, we saw much longer diagnostic intervals, the same survival probability and an opposite, concave trend between the length of the diagnostic interval and mortality. However, the latter association was not statistically signiﬁcant and the association did not apply to prostate cancer. The study displays the immense complexity and difficulty of diagnosing cancer. Yet, we can infer from the increasing trends in mortality that a few weeks can make a difference – that time matters. | Not reported |  |
| Yilmaz (2008) | Comparison of mean interval* according to pathological stage (I, II, IIIa. IIIb, IV) (Kruskall Wallis test) Proportion of patients with pre-presentation time of >30 days according to pathological stage (I, II, IIIa. IIIb, IV) (Fishers exact test) Proportion of patients with post-presentation time of >6 weeks according to pathological stage (I, II, IIIa. IIIb, IV) (Fishers exact test)  Proportion of patients with symptom duration of >72 days according to pathological stage (I, II, IIIa. IIIb, IV) (Fishers exact test) * Analyses of intervals of pre-presentation time, post-presentation time and symptom duration conducted separately. | Not reported   Not reported | p>0.05 (for all 3 intervals*)  p>0.05 (for all 3 intervals*) |  |  | Presence of delay or length of delay does not correlate with tumour stage in patients who underwent thoracotomy. | Not undertaken |  |
| Yun (2012) | X2 test  Cox proportional hazards modelling   | Surgical treatment delay of > 31 days versus ≥ 31 days and five-year survival  Unadjusted HR 1.12  Adjusted HR 1.16 | Not reported | 1.02  1.06 | 1.23  1.27 | Among surgery patients, treatment delays of > one month were associated with worse survival for patients with rectal, pancreatic, lung or breast cancer than treatments delays of < one month.  Adjusted HR for age, sex, Charlson score, hospital type, insurance, radiotherapy, chemotherapy, type of medical care institution, year of diagnosis and waiting time. | Not reported |  |
| **Gastro-intestinal tract** | | | | | | | |  |
| **Gastric and Oesophageal** | | | | | | | |  |
| Grotenhuis (2010) | Stage:  Chi square test.  Survival:  Pre-hospital delay: Chi square test.  Hospital delay: Log rank test. | Stage:  Hospital delay <5 weeks/5-8 weeks/> 8 weeks and the following pTNM stage:  pT1-2: 30(23.4%)/57(30.6%)/54(30.5%).  pT3-4: 98(76.6%)/129(69.4%)/123(69.5%).  pN0: 42(32.8%)/66(35.5%)/62(35.0%).  pN1: 86(67.2%)/120(64.5%)/115(65.0%).  pM0: 103(80.5%)/150(80.6%)/131(74.0%). pM1a-pM1b: 25(19.5%)/36(19.4%)/46(26.0%).  Survival:  Pre-hospital delay:  ≤ 3 months vs > 3 months: 24.0%/29.3%. Hospital delay:  <5 weeks/5-8 weeks/>8 weeks: 24.7%/21.7%/32.3%.  Prehospital delay ≤3 months vs >3 months: Morbidity: 199(64.6%)/104(56.8)  Reoperation: 34(11.0%)/16(8.7%)  In hospital mortality: 18(5.8%)/9(4.9%)  Hospital delay <5 weeks/5-8 weeks/> 8 weeks:  Morbidity: 62(48.4%)/122(65.6%)/119(67.2%)  Reoperation: 7(5.5%)/20(10.8%)/23(13.0%)  In hospital mortality: 2(1.6%)/10(5.4%)/15(8.5%)  Radicality of resection:  RO 86(67.2%)/124(66.7%)/130(73.4%  R1-R2 42(32.8%)/62(33.3%)/47(26.6% | P=0.31  P=0.88  P=0.24  P=0.10  P=0.09  P=0.42  P=0.66  P<0.01  P=0.10  P=0.03  P=0.32 |  |  | Length of hospital delay did not affect pTNM stage.  Long-term outcome (overall 5-year survival) after oesophagectomy was comparable for patients who experienced symptoms for a period of 3 months or less versus more than 3 months until endoscopy was performed. Longer hospital delay did not result in worse survival.  Patient's short-term (morbidity, reoperation rate, and in-hospital mortality) outcomes after oesophagectomy were comparable for patients who experienced symptoms for a period of 3 months or less versus more than 3 months until endoscopy was performed. A shorter hospital delay between establishing the diagnosis of oesophageal cancer on patient's first endoscopy and surgery was associated with significantly lower overall morbidity and in-hospital mortality. Length of hospital delay did not affect pTNM stage or R0 resection rate. | Not reported |  |
| Sharpe (2010) | Kaplan-Meier, log rank test. | Median survival in days: Routine: 405  Two-week wait: 239  Emergency: 121 | P<0.001 | 305  188  58 | 501  289  184 | Referral by 2WW resulted in more rapid treatment than routine referral but this did not translate into an improvement in survival. | Not reported |  |
| **Gastric** | | | | | | | |  |
| Arvanitakis (1992) | Comparison of mean survival time after diagnosis (during the 3-year study period) in those diagnosed < 3 months vs > 3 months (method not reported): Patients with resectable tumours: Patients with non-resectable tumour: Comparison of survival rates at the end of the 3-year study period in those diagnosed < 3 months vs > 3 months: Patients with resectable tumour: Patients with non-resectable tumour: Incidence of resectable tumour in patients diagnosed 3 months: | 9.9+4.8 vs 13.5+11.7 4.8+4.0 vs 8.5+6.9    8/18 vs 17/35 9/10 vs 33/37  35.85 | < 0.05 < 0.05    not reported not reported  <0.001 |  |  | An unexpected finding was that patients with early diagnosis did not have better survival (than) patients with late diagnosis. One explanation for this observation may be that tumours with aggressive behaviour develop rapidly with early onset of symptoms. | No correlation was noted between patient survival and the anatomical location of the tumour or the degree of histological differentiation |  |
| Fernandez (2002) | Comparison of median symptom to diagnosis interval for local vs regional vs disseminated | 5/26(19%)  26/111(23%) | p<0.05  p-0 |  |  | Correlation between survival and patient and doctor delay was negative in gastric cancer patients.  Patients with symptoms < 1 month duration compared with the overall group showed no improvement in curability | Not undertaken |  |
| Haugstvedt (1991) | Comparison of percentage of patients within each TNM stage with delay exceeding the median for the whole group Patient delay: Doctor delay: Total delay:  Trend in type of operative procedure (resection vs. non-resectional procedure vs. no operation) based on length of- Patient delay: Doctor delay: Total delay: Post-operative mortality rate based on delay - Patient delay (0-30days vs > 30days): Doctor delay (0-30days vs > 30days): Total delay (0-90days vs > 90days): Logistic regression analysis with postoperative mortality as dependent variable- Patient delay: Doctor delay: Total delay: | 39/375 vs 51/562 54/389 vs 43/573 53/425 vs 50/573 | 0.39 0.004 0.63    0.22 <0.0001 0.46   0.08 0.04 0.06    0.006 0.07 Non-significant | n/a | n/a | Delay influences outcome, to the effect that a short diagnostic delay signifies advanced disease, and secondly, that long delay does not give any information about resectability of the malignancy...A more fundamental question is whether short delay reflects an aggressive biologic behaviour of the gastric cancer itself or whether long delay reflects reduced awareness of severe symptoms by the patient and physician. Supplementary data from this study gives strength to the latter hypothesis. | Not undertaken |  |
| Lim (1974) | Correlation coefficient: Survival and patient delay  Survival and doctor delay | 0.05  (curative group:-0.15) -0.01 (curative group:-0.22) | n/s  n/s |  |  | Survival once symptoms have developed is not related to length of symptomatic period but to clearly defined pathologic criteria...The failure to correlate survival with delay does not negate the value of early diagnosis | Curability of those presenting with symptoms less than 1 month: no improvement in curability |  |
| Maconi (2003) | Comparison of 5-year survival rates for patients (without alarm symptoms) diagnosed:  > 6 months  within 6 months  (log-rank test) | 1 d.f= 3.93  93.4%  66.5% | 0.048 | n/a | n/a | We and other authors have shown a trend in the opposite direction; i.e. The longer the duration of symptoms, the earlier the tumours are diagnosed and the longer the survival. This may be due to the wide variation in doubling-time of GC and the long duration of dyspeptic symptoms not related to GC | Not undertaken |  |
| Maguire (1994) | Comparison of survival rates for quintile 1 of symptom-to-diagnosis-interval (i.e. 20% of the shortest intervals) vs higher quintiles (log-rank test) Comparison of SDI in months for:  Local/Regional/Disseminated  (Kruskal-Wallis test) | Not reported    2/3/3 | 0.655  0.182 | n/a | n/a | No association between symptom-to-diagnosis interval and survival | A ratio of median SDI to median survival (SSR) was derived and used as an estimate of the relative opportunity for clinical lead-time bias. The opportunity for a decrease in SDI to appear spuriously associated to increased survival would be lower in tumours with a lower SSR (this was not the case for stomach cancer which had a high SSR). |  |
| Martin (1997) | Comparison of median overall delay among patients presenting with a particular tumour stage (Mann-Whitney U test) Comparison of median delay in those with: successful operations  unsuccessful operations | Not reported  17.7 weeks  17.8 weeks | Non-significant    Non-significant | n/a | n/a | For patients with stomach cancer, there was no clear relation between tumour stage and delay in diagnosis  There was no relation between delay in diagnosis and the success of potentially curative resection | Not undertaken |  |
| Tokuda (2009) | Comparison of the mean symptom to visit intervals between patients:  with metastasis  without metastasis (Student's t-test) | 51.8  63.3 | Non-significant |  |  | There were no significant differences in symptom-to-visit intervals for patients with and without metastasis | Not undertaken |  |
| Windham (2002) | Comparison of survival rates for those diagnosed after onset of symptoms to presentation:  within 2 months (mean/median)  > 2 months (mean/median)  (log-rank test) Comparison of survival rates for those diagnosed after presentation:  within 2 months (mean/median)  > 2 months (mean/median)  (log-rank test) | 11.4/7.0  16.7/9.0  14.5/7.5  13.2/7.5 | 0.14     0.67 | n/a | n/a | Early diagnosis from either the onset of symptoms or presentation to a physician does not improve survival in young patients | Not undertaken |  |
| Yun (2012) | X2 test  Cox proportional hazards modelling   | Surgical treatment delay of > 31 days versus ≥ 31 days and five-year survival  Unadjusted HR 0.94  Adjusted HR 1.03 | Not reported | 0.90  0.99 | 0.98  1.08 | Among surgery patients, treatment delays of > one month were associated with worse survival for patients with rectal, pancreatic, lung or breast cancer than treatments delays of < one month.  Adjusted HR for age, sex, Charlson score, hospital type, insurance, radiotherapy, chemotherapy, type of medical care institution, year of diagnosis and waiting time. | Not reported |  |
| Ziliotto (1987) | Correlation coefficient for association of pre-presentation time with length of survival after beginning of treatment (for those who died, excluding intraoperative deaths) | 0.269 | not given (check meaning of slope of the regression line) | n/a | n/a | This comparison permitted (us) to confirm a positive correlation between survival and longer time of digestive history | Extract information on stage-based analysis |  |
| **Oesophageal** | | | | | | | |  |
| Fernandez (2002) | Comparison of median symptom to diagnosis interval (days) for  local vs regional vs disseminated | 190/94/130 | p=0.045 |  |  | In digestive cancers, symptom to diagnosis interval (SDI) does not influence survival per se and is not positively associated with stage. |  |  |
| Martin (1997) | Comparison of median overall delay among patients presenting with a particular tumour stage  (Mann-Whitney U test)  Comparison of median delay in patients with successful vs. unsuccessful operation | Median delay 6.7 weeks (stages I & II) vs 20.9 weeks (stages III & IV)  15 v 24 weeks | p<0.02     p=0.2 | n/a | n/a | There was a significant difference between the delay for early vs late stage, but sample used was small |  |  |
| Tokuda (2009) | Comparison of the mean symptom to visit intervals between patients with and without metastasis: (Student's t-test) | 84.7/61.3 | Non-significant |  |  | The risk for distant metastasis is higher among patients with solid tumours and a short interval of symptom onset to clinical presentation. |  |  |
| Wang (2008) | Comparison of median delay in patients diagnosed in:  Stage I/II  StageIII/IV  (Mann-Whitney U test) Comparison of median delay between patients with well, moderately and poorly differentiated tumours  (Kruskal-Wallis) | 1.8months  2.2months)  Well: 2.3 Mod: 2.1 Poor: 2.1 | p=0.0177  p=0.881 | n/a | n/a | This may indicate that a few months delay in diagnosis and treatment has a significant influence on stage, and therefore prognosis. |  |  |
| **Pancreatic** | | | | | | | |  |
| Gobbi (2013) | Prognostic value of diagnostic delay on survival. (<4weeks, 4-16 weeks, >16 weeks) (Cox proportional hazards model) The role of individual symptoms was checked through an evaluation of the relative survival, which was calculated as the ratio of the overall survival rate observed in the patient's series to the expected survival rate drawn from the general reference population for similar subjects in respect to age, sex, calendar year of initial observation and duration of observation. | Median diagnostic delay (weeks) = 8.4;  Range = 0.4-44.7 Hazards Ratio = 1.0240  Median diagnostic delay and median survival recorded in patients presenting one single symptom: Symptom No. of patients Diagnostic delay Survival (weeks) (months)  Pain 38 4.6 (4.3-5.2) 15.0 (12.5-21.9)  Jaundice 21 7.1 (6.7-7.7) 11.1 (10.8-11.7)  Weight loss 33 12.1 (11.8-12.7) 6.5 (3.4-7.2) | P=0.001 | 1.0117 | 1.0365 | Prognostic weight of the diagnostic delay was able to discriminate survival significantly. The different symptoms, when presenting alone at onset of disease, were related with rather different diagnostic delays, the symptoms leading to the earliest diagnosis being abdominal pain, followed by jaundice and then by weight loss. The study of the survival corresponding to each symptom showed that symptoms which led to an earlier diagnosis were individually related to better prognosis. The study of the relative survival confirmed that the deviations from the survival of the reference population agree with the worsening prognosis observed when moving from pain to jaundice and to weight loss. |  |  |
| McLean (2013) | Continuous data were compared using a Student's t-test or one-way ANOVA, categorical data were compared using the Chi-squared test | Stage: T-status  ≤30 days N (%)  >30 days N (%)  T1 1 (2.9) 2 (3.4)  T2 9 (26.5) 6 (10.3)  T3 19 (55.9) 28 (48.3)  T4 5 (14.7) 22 (37.9)  Survival:  Median survival for patients who waited (regardless of resectability):  ≤30 days = 11.6 months  >30 days = 12.3 months.  Likelihood of nonresectability in patients waiting:  ≤30 days = 37.7% (26/69)  >30 days = 38.2% (42/110) | P=0.055  Not reported  Not reported | Not reported | Not reported | Longer wait times may be associated with stage migration.  There were no differences in survival between the ≤30 days and >30 days group.  The difference was not significant between the ≤30 days vs >30 days wait times. At none of the time intervals assessed was there any obvious change in resection rate, nor was there a trend toward lower resection rate with progressively longer wait times. | Survival as a function of wait time was also evaluated in the resectable and unresectable subgroups. Resectable: Median survival = 19.4 months; there was no difference between patients who waited ≤30 days compared to >30 days.  Unresectable: Median survival = 7.1 months; there was no difference between patients who waited ≤30 days compared to >30 days. |  |
| Raptis (2010) | Comparison of various time delays on 1, 3, and 5 year survival; Student's t-test, Hazards risk ratio. | The time delay from symptoms to referrals, hazard ratio = 1.001;  Initiation of symptoms to GP visit (143 days; 75% quartile) to 1/3/5 year survival: HR  Median <143 days 31/6/5 >143 days 17/1/1  Initiation of symptoms to GP visit (65 days; median survival) to 1/3/5 year survival:  Median <65 days 32/6/5 >65 days 23/4/4  Initiation of symptoms to GP visit (31 days; 25% quartile) to 1/3/5 year survival:  Median <31 days 30/7/7 >31 days 26/4/3  No. of patients (%);1/3/5 years Median p value (%)  N (%) 1/3/5 years survival  Symptomatic 329 (93) 27, 4, 3  Jaundice 83 (25) 35, 3, 0  Abdominal pain 63 (19) 18, 1, 0  Weight loss 3 (1) 0, 0, 0  Other 16 (5) 26, 1, 1  Combined 164 (50) 27, 5, 3  Symptoms not 26 (7) 26, 6, 6  recorded  Time from symptom to GP referral  <31 days 177 (50) 30, 7, 7  >31 days 178 (50) 26, 4, 3  <65 days 177 (50) 32, 6, 5  >65 days 178 (50) 23, 4, 3  <143 days 177 (50) 31, 6, 5  >143 days 178 (50) 17, 1, 1  Inoperable (IP) 254 (71) 24, 2, 0  Operable (OP) 101 (29) 35, 13, 9  Bypass 68 (19) 21, 5, 0  Resectable 33 (10) 66, 31, 25    * Inoperable vs operable  ** Inoperable vs bypass  # Inoperable vs resectable, resectable vs bypass | P=0.043  P=0.14  P=0.18  P=0.492  P=0.098  P=0.715  P=0.068  P=0.456  P=0.074  P=0.956  P=0.492  P=0.180  P=0.014  P<.001*  P=.258**  p <.001# | 0.001 | 0.002 | The time delay from symptoms to referral alone had minimal clinical relevance. Patients that presented within 143 days from the initiation of symptoms to their GP visit had a favourable survival when compared to those that presented after 143 days. There were no significant differences in survival of patients that were diagnosed or treated before or after 62 days (NHS Cancer Plan - Cancer waiting time targets) from referral.  Isolated or combined mode of clinical presentation had no significant effect on survival. Survival of patients with no recorded symptoms had survival similar to the symptomatic patients. | Not reported |  |
| Tokuda (2009) | Comparison of the mean symptom to visit intervals (days) between patients with and without metastasis: (Student's t-test) | 52.5/29.4 | Non-significant |  |  | The risk for distant metastasis is higher among patients with solid tumours and a short interval of symptom onset to clinical presentation. | Not undertaken |  |
| Yun (2012) | X2 test  Cox proportional hazards modelling   | Surgical treatment delay of > 31 days versus ≥ 31 days and five-year survival  Unadjusted HR 1.33  Adjusted HR 1.23 | Not reported | 1.16  1.07 | 1.53  1.41 | Among surgery patients, treatment delays of > one month were associated with worse survival for patients with rectal, pancreatic, lung or breast cancer than treatments delays of < one month.  Adjusted HR for age, sex, Charlson score, hospital type, insurance, radiotherapy, chemotherapy, type of medical care institution, year of diagnosis and waiting time. | Not reported |  |
| **Hepatocellular** | | | | | | | |  |
| Singal (2013) | Kaplan-Meier, Cox univariate and multivariate regression. Association of delayed treatment and survival. | Delayed treatment: 1 and 2 year survival rates: 63.7% and 50.1%  Without delayed treatment: 1 and 2 year survival rates: 89.8% and 64.5%.  HR 0.33 |  | 0.24 | 0.46 | After adjusting for stage and Child-Pugh class, treatment delays were associated with significantly worse survival. | Not reported |  |
| Tokuda (2009) | Comparison of the mean symptom to visit intervals between patients with and without metastasis: (Student's t-test) | 50.5/32.4 | Non-significant |  |  | The risk for distant metastasis is higher among patients with solid tumours and a short interval of symptom onset to clinical presentation. | Not undertaken |  |
| **Colorectal** | | | | | | | |  |
| Cerdan-Santacruz (2011) | ANOVA, Student’s t-test Relationship between diagnostic delay and Dukes’ tumour stage | Relationship between diagnostic delay and Dukes’ tumour stage.  Delay Stage: A B C D  Patient delay: 5.18; 0.85; 4.09; 1.74 Physician delay: 2.84; 3.03; 0.67; 1.62  Test delay: 3.14; 2.40; 2.92; 2.35  Overall delay: 11.16 6.28 7.68 5.71 | Non-significant |  |  | For correlations between tumour stage and diagnostic delay, results were also non-significant and paradoxically a reduced diagnostic delay was related to a more advanced tumour stage, stage D, while stage A was linked to a greater delay. | Not reported |  |
| Currie (2011) | X2 test; Mann-Whitney U-Test or the Kruskal-Wallis test.  Kaplan-Meier method with the log rank test.  Comparison of referral pathways and survival | 5 year disease free survival and:  Group 1 (TWW): 49%  Group 2 (non-TWW): 52%  Overall disease free survival and:  Group 1 (TWW): 1521 days  Group 2 (non-TWW): 1591 days | P=0.003  P=0.29 | Not reported   | Not reported   | Within a well-run colorectal unit, the TWW referral pathway does not appear to improve 5-year survival outcomes in rectal cancer. This study also demonstrated that patients have a similar stage of disease at presentation. | Not reported |  |
| Deng (2012) | Nonparametric test, Student’s t-test and Pearson’s x2 delay in diagnosis and advanced cancer stage; binary logistic regression model | Association between a long delay in diagnosis and advanced cancer stage:  colon cancer  rectal cancer  both | P=0.367  P=0.953  P=0.968 |  |  | No association between a long delay in diagnosis and advanced cancer stage was detected. | Not reported |  |
| Gort (2010) | Cox proportional Hazard model | Association of time from diagnosis to first treatment with  Disease-free survival (DFS)  Relative survival (RS)  Multivariate analysis: HR=0.69 | P=0.018  P=0.048  P=0.018 | 0.51  0.44 | 0.94  0.99 | The univariate analysis revealed that time to treatment (TTT) was a strong predictor for DFS and RS. Patients receiving treatment within 7 weeks after diagnosis had significant better survival. Multivariate analysis demonstrated TTT as an independent predictor of survival. | Not reported |  |
| Guzman (2011) | T Student, chi-square, linear regression, logistic regression. Association between diagnosis to treatment interval (DTI) and tumour stage. | Mean DTI in patients with stages 0-II and III-IV was 45.60 and 46.62 days,  DTI > 30 days, colonic tumours: increased risk of an advanced stage  DTI > 30 days, rectal tumours: decreased risk of advanced stage | p = 0.81  p=0.053 |  |  | There were no significant differences in tumour stage according to the pathway followed. Independently of the track followed, a diagnosis to treatment interval (DTI) longer than 30 days was associated with advanced tumour stages for colon cancer, while it was associated with low stages for rectal cancer. | Not reported |  |
| Pruitt (2013) | Median diagnostic delay and median treatment delay associated with localised stage versus distant stage.  Median diagnostic delay and median treatment delay associated with all-cause death and CRC-specific death (logistic regression). | Stage and diagnostic delay (median):  Colon cancer, Localised: 68 days  Colon cancer, Distant: 47 days  Rectal cancer, Localised: 43 days  Rectal cancer, Distant: 29 days  Stage and treatment delay (median):  Colon cancer, Localised: 15 days  Colon cancer, Distant: 8 days  Rectal cancer, Localised: 18 days  Rectal cancer, Distant: 13 days  Death and diagnostic delay:  Colon cancer patients with the longest diagnostic delays (8-12 months vs. 14-59 days) had higher odds of all-cause (aOR: 1.31 CI: 1.08-1.58), but not CRC-specific death.  Colon cancer patients with the shortest treatment delays (<1 vs. 1-2 weeks) had higher odds of all-cause (aOR: 1.23 CI: 1.01-1.49), but not CRC-specific death. | Not reported  aOR: 1.31  aOR: 1.23 | Not reported  1.08  1.01 | Not reported  1.58  1.49 | As with diagnostic delays, treatment delays were longer among those with localized stage compared with distant stage for both colon and rectal cancer.  Longer treatment delays among colon cancer patients did not increase the risk of all-cause or CRC-specific death. For rectal cancer patients, neither diagnostic nor treatment delays were associated with risk of all-cause or CRC-specific death in adjusted models. Colon cancer patients with the shortest treatment delays had higher odds of all-cause death. Previous literature has also demonstrated that CRC patients with shorter delays have worse prognosis.  Although we attempted to limit our sample to non-emergent cases only, it is likely that this finding indicates higher odds of death among patients with emergent or urgent situations that were not excluded using our algorithm. | Not reported |  |
| Ramsay (2012) | For categorical data: x2 test, Fisher’s exact tests for scale data: Mann-Whitney U-test the effect of delay from referral to diagnosis on tumour characteristics, patient demographics and resection rates when routinely referred patients were compared with those identified through the urgent route. | Dukes stage and referral groups:  (urgent referrals n=220; routine referrals n=53)  Dukes stage A  Urgent referrals 35 (15.9%); routine referrals 16 (30%)  Dukes stage B  Urgent referrals 108 (49%); routine referrals 28 (52.8%)  Dukes stage C  Urgent referrals 97 (44.1%); routine referrals 8 (15%) | P= 0.002 | Not reported | Not reported | In this study of comparable populations, urgency of referral resulted in a decreased time to cancer diagnosis. This did not influence resection rates or Dukes stage despite significant resource investment. Long-term follow-up is required to determine any impact on survival. | Not reported |  |
| Roland (2013) | Relationship between interval to treatment and survival.  x2 or Fisher exact test, two-sample t test or Wilcoxon rank sum test, Kaplan-Meier method, log-rank test, Stepwise Cox regression. | Patients were divided into two groups (A) or quartiles (B) based on their median interval to treatment.  A. Interval to treatment /Median survival  <16 days/(4.5 years  >16 days/ 5.1 years  B. Interval to treatment/Median survival  <7 days/3.5 years; 8-16 days/5.2 years; 17-34 days/4.6 years; >35 days/5.1 years | P=0.128  P=0.114 | Not reported | Not reported | When patients were grouped into quartiles on the basis of time to treatment intervals, we were unable to identify a point in which an increased interval to treatment was associated with decreased survival. Also, unable to identify a threshold of interval to treatment at which survival was significantly different when interval to treatment was analysed as a continuous or categorical variable. | Not reported |  |
| Singh (2012) | Multivariate logistic regression  (between time to diagnosis and tumour stage).  Multivariate Cox regression  (association between time to diagnosis and survival).  Potential confounding factors considered included age, sex, socioeconomic status (SES),  Co-morbidity index score, the calendar year of colon cancer diagnosis, stage at diagnosis (for survival analysis), tumour grade, site of CRC (colon versus rectum), and admission through an emergency room in the month before diagnosis and the type of first gastrointestinal test.  Potential confounders were retained in the multivariate models if they resulted in a 10% or higher change in the crude HRs or ORs. | Stage III/IV versus I/II:  Time to diagnosis (days) adjusted OR  For each increase of 30 days 0.94  Quartile 1 (<15) 1.00  Quartile 2 (15 to <51) 0.79  Quartile 3 (51 to <116) 0.59  Quartile 4 (≥116) 0.50  ≤90 percentile (<239) 1.00  >90 percentile (≥239) 0.84  Survival  Time to diagnosis (days) adjusted HR  For each increase of 30 days 1.01  Quartile 1 (<15) 1.00  Quartile 2 (15 to <51) 1.03  Quartile 3 (51 to <116) 0.99  Quartile 4 (≥116) 1.07  ≤90 percentile (<239) 1.00  >90 percentile (≥239) 1.40 | P=0.05 | 0.89  Reference  0.52  0.39  0.33  Ref  0.53  0.98  Ref  0.78  0.72  0.79  Ref  1.00 | 0.98  Reference  1.20  0.89  0.75  Ref  1.33  1.05  Ref  1.37  1.35  1.44  Ref  1.96 | The longer time to diagnosis did not have an adverse effect on clinical outcomes, such as stage at diagnosis. Individuals with the longest time to diagnosis were less likely to have stage III/IV CRC at diagnosis.  The longer time to diagnosis did not have an adverse effect on survival after CRC diagnosis. | Not reported |  |
| Terhaar sive  Droste(2010) | Kaplan-Meier, Cox regression, independent samples t-test.  Stage: Comparison of patient's delay, healthcare delay and total diagnostic delay in patients with early stage CRC to patients with late stage CRC.  Kaplan Meier  Survival: Total diagnostic delay shorter than median was compared to the survival of patients with a total diagnostic delay longer than median in patients with early and late stage CRC. | Early vs late stage CRC and:  Mean total diagnostic delay  Mean patient's delay  Mean healthcare delay  Early stage CRC and total diagnostic delay shorter versus longer than the median delay. Late stage CRC and a total diagnostic delay shorter than the median. | P=0.27  P=0.56  P=0.46  P=0.93  P=0.01 | Not reported | Not reported | No significant differences were observed in the mean total diagnostic delay, the mean patient's delay and the mean healthcare delay in early versus late stage CRC.  In early stage CRC, no difference in survival was observed between patients with total diagnostic delay shorter versus longer than the median delay In late stage CRC, patients with a diagnostic delay shorter than the median had a shorter survival than patients with a diagnostic delay longer than the median. | Not reported |  |
| Thompson (2011) | Fisher’s exact test, Student’s t-test.  Stage: association of delay from onset to treatment and stage.  Survival analysis was used to calculate the hazard ratio (delay from onset to treatment and survival). | No data presented.  Survival in patients treated within 6 months of onset of symptoms: 55.1%  Survival in patients treated > 6 months from onset of symptoms: 53.5% | Not reported | Not reported | Not reported | Delays in treatment of longer than 6 months did not affect the stage of disease.  Delays in treatment of longer than 6 months did not affect survival. | Not reported |  |
| Tomlinson (2012) | Fisher exact of x2 tests. Delay from symptom onset to first physician assessment associated with tumour stage. | TNM stage and < 1 month/> 1 month  Stage I or II: 16 (37%)/16 (36%)  Stage III: 16 (37%)/14 (32%)  Stage IV: 11 (26%)/14 (32%) | P=.98 | Not reported | Not reported | Forty-three (49%) patients had CRC symptoms for 1 month or less before seeing a physician, and 44 (51%) had symptoms for longer than 1 month before seeing a physician. There were no statistical differences between these groups with respect to marital status, education, or TNM stage. | Not reported |  |
| Tørring (2011) | Logistic regression overall 3-year mortality associated with the diagnostic interval. | Diagnostic interval (DI) and 3 year cumulative mortality (MR).  Presenting with alarm or serious symptoms:  DI Weeks MR Crude OR Adjusted OR  0-4 48% 2.15 2.56  5-11 30% 1 1  ≥ 12 47% 2.09 2.04  Presenting with vague symptoms:  DI Weeks MR Crude OR Adjusted OR  0-4 10% Comparison not  justified  5-11 56% 1 1  ≥ 12 57% 0.96 0.71  Diagnostic interval (DI) and 1 year cumulative mortality (MR).  Presenting with alarm or serious symptoms:  DI Weeks MR Crude OR Adjusted OR  0-4 25% 1.70 2.09  5-11 17% 1 1  ≥ 12 31% 2.20 2.35  Presenting with vague symptoms:  DI Weeks MR Crude OR Adjusted OR  0-4 10% Comparison not  justified  5-11 37% 1 1  ≥ 12 23% 0.52 0.40 |  | 1.14/1.29  Reference  0.94/0.87  Reference  0.37/0.32  0.79/0.92  Reference  0.89/0.88  Reference  0.16/0.11 | 4.08/5.05  Reference  4.62/4.77  Reference  2.98/2.91  3.63/4.73  Reference  5.41/6.26  Reference  1.64/1.48 | In patients presenting with symptoms suggestive of cancer or any other serious illness, the risk of dying within 3 years decreased with diagnostic intervals up to 5 weeks and then increased (P=0.002). In patients presenting with vague symptoms, the association was reverse, although not statistically significant. This study provides evidence for the hypothesis that the length of the diagnostic interval affects mortality in CRC patients. | Not reported |  |
| Tørring (2012) | Kaplan-Meier association between time from the first presentation of symptoms in primary care to the diagnosis (the diagnostic interval) and 5-year mortality after diagnosis of colorectal cancer (CRC).  Adjusted for tumor site (colon/rectum), age (40-64/65 -74/ 75 years), and sex using proportional hazard Cox regression. | 5 year hazard ratio (HR) of diagnostic interval quartiles (DIQ) for combined data.  DIQ N Crude HR Adjusted HR  1^st^ 300 1.33 1.33  2^nd^ + 3^rd^ 623 1 1  4^th^  320 1.31 1.28 |  | 1.10/1.10  Reference  1.09/1.06 | 1.60/1.61  Reference  1.59/1.55 | Despite variations in the potential selection and information bias when using different methods of identifying the date of first presentation, the association between the length of the diagnostic interval and 5-year mortality rate after the diagnosis of CRC was the same for all three types of data: displaying a U-shaped association with decreasing and subsequently increasing mortality with longer diagnostic intervals. The authors observed the same U-shaped association using three different data collection methods in different health care systems and over different time periods. This provides considerable support for initiatives to expedite the cancer diagnosis for CRC. | Not reported |  |
| Tørring (2013) | Kaplan-Meier method, conditional logistic regression to estimate ﬁve-year mortality odds ratios as a function of the diagnostic interval using restricted cubic splines and adjusting for comorbidity, age, sex and type of cancer. | Mortality rates (MR%) and crude/adjusted odds ratios (OR) for the 1st and 4th compared with 2nd and 3rd diagnostic interval quartiles (DIQ) for patients with a) alarm or serious symptoms and b) vague symptoms.  **Colorectal cancer:**  Alarm or serious symptoms  DIQ MR% Crude OR Adjusted OR  1^st^ 66.7 3.44 4.74  2^nd^ + 3^rd^ 36.7 1 1  4^th^  53.1 1.95 2.01 |  | 1.71/2.20  Reference  0.97/0.93 | 6.93/10.19  Reference  3.90/4.36 | In patients with alarm or any serious symptoms, mortality grew the longer the diagnostic intervals in those 40% from this group who experienced the longest delays. In patients with alarm or any serious symptoms, having very short diagnostic intervals was also associated with a high mortality. Inversely, in patients presenting with vague symptoms, we saw much longer diagnostic intervals, the same survival probability and an opposite, concave trend between the length of the diagnostic interval and mortality. However, the latter association was not statistically signiﬁcant and the association did not apply to prostate cancer. The study displays the immense complexity and difficulty of diagnosing cancer. Yet, we can infer from the increasing trends in mortality that a few weeks can make a difference – that time matters. | Not reported |  |
| Valentin-Lopez (2012) | Analysis of variance and the chi-square test stage of disease at diagnosis associated with rapid pathway compared to standard pathway. | Astler-Coller stage and rapid referral/standard referral pathway.  Stage A: 26.0% (13)/11.6% (28)  Stage B: 36.0% (18)/41.1% (99)  Stage C: 24.0% (12)/32.4% (78)  Stage D: 14.0% (7)/14.9% (36) | P=0.007 | Not reported | Not reported | The stage of disease at diagnosis was influenced by the referral route. With the rapid referral pathway, 26.0% of cancers were diagnosed at Stage A, while the standard referral pathway only diagnosed 11.6% at this stage. The difference was maintained after stratifying by cancer site; 25.8% of colon cancers were diagnosed at Stage A in the rapid pathway patients compared to 11.0% in standard route patients (P = 0.030) and 22.2% of rectum cancers at Stage A in the rapid pathway patients compared to 12.5% in the standard pathway patients (P = 0.276). | Not reported |  |
| Van Hout (2011) | w2 test. Time between initial symptoms and onset of treatment, categorised into four periods and stage. | Median duration from first symptom until onset treatment and duration T1/ T2/ T3/T4/T1-T4.  Localised disease: 31/14/29/18/147.5  Metastasised disease: 30/14/28/19/120 | Not reported | Not reported | Not reported | The study demonstrates considerable delay in the management of CRC, especially in the time to consultation and time to onset of clinical treatment. We were not able to confirm that as a result of patient and physician delay, CRC would be diagnosed in a more advance stage, but it does not imply that earlier detection could not have caused better survival in some patients. | Not reported |  |
| Yun (2012) | X2 test  Cox proportional hazards modelling   | Surgical treatment delay of > 31 days versus ≥ 31 days and five-year survival  Colon cancer  Unadjusted HR 1.08  Adjusted HR 1.10  Rectal cancer  Unadjusted HR 1.29  Adjusted HR 1.28 | Not reported | 0.98  1.00  1.18  1.17 | 1.19  1.21  1.41  1.40 | Among surgery patients, treatment delays of > one month were associated with worse survival for patients with rectal, pancreatic, lung or breast cancer than treatments delays of < one month.  Adjusted HR for age, sex, Charlson score, hospital type, insurance, radiotherapy, chemotherapy, type of medical care institution, year of diagnosis and waiting time. | Not reported |  |
| Zafar (2012) | Kaplan-Meier, log rank test Log rank test to compare survival rates between the two groups (before and after the introduction of the 2-week wait referral system) | 5 year survival rate:  Pre-2WW: 71%  Post-2WW: 72% | Not reported | Not reported | Not reported | This study shows that the introduction of the 2WW referral system has had no significant impact on the 5-year survival of CRC patients who underwent potentially curative surgery. This finding may reflect the fact that patients were being treated in a timely fashion prior to the introduction of the 2WW system and that, once a cancer became symptomatic, early treatment did not improve survival. | Not reported |  |
| **Renal tract** | | | | | | | |  |
| **Prostate** | | | | | | | |  |
| Korets (2012) | Stage:  Cox proportional hazards model  Survival:  Kaplan Meier and log rank tests | Delays and risk of Biochemical recurrence. Hazard ratios:  <60 days  61-90 days - 1.26  >90 days - 1.13  Delays and 5 year survival:  <60 days - 80% of men  61-90 days - 78% of men  >90 days - 85% of men | Reference  P=0.12  P=0.43  P=0.11 | Reference  0.94  0.73  Not reported | Reference  1.70  1.31 | Delay to prostatectomy of >60 days was not associated with higher rates of clinical progression.  5 year survival rates were similar across all 3 groups. | Not reported |  |
| Neal (2007) | Comparison of survival rates between urgent referrals and those diagnosed through other routes (log-rank test) (n=136) Comparison of proportion in T-stage among those diagnosed via urgent referral vs other routes (chi-square test) Comparison of Gleason score among those diagnosed via urgent referral vs other routes (chi-square test) * Median duration (GP referral to diagnosis) for the Urgent referral= 100 days vs 143 days for the other routes | 0.12    1.79    0.44 | 0.731 (df=1)    0.616(df=3)  0.505(df=1) | n/a | n/a | No difference was found for differences in outcomes for prostate cancer between urgent guidelines referrals (and all referrals marked as urgent) and those diagnosed through other routes. These findings to an extent were unexpected but can be explained by the fact that aggressive tumours may lead to the rapid progression of symptoms leading to earlier presentation and fulfilment of urgent referral criteria; such patients are also likely to have disease that is less amenable to life prolonging treatments. | Not undertaken |  |
| O'Brien (2011) | Stage, biochemical recurrence and surgical delay: Cox proportional hazards model.  Survival and surgical delay: Kaplan-Meier and log rank tests. | Stage T2 or greater (%)and surgical delay of  < 6 months: 18.9%  > 6 months: 6.8%  Risk of recurrence (%) and surgical delay of  < 6 months: 5%  > 6 months: 12%  Survival  Delay of >6 months and 5 year progression free survival | P=0.04  P=0.02  P=0.009 | Not reported | Not reported | A delay in surgery of 6 months or more was associated with a proportionately greater risk of biochemical recurrence despite a similar follow up interval for both groups. Biochemical recurrence occurred in 5% of men treated within 6 months and 12% of men with a delay of 6 months or more.  A surgical delay of 6 months or more was associated with a significantly lower 5 year progression free survival. | Not reported |  |
| Sun (2012) | Mann Whitney test and Chi Square test.  Treatment delay and stage/survival. | Stage:  Adjusted outcomes (upstaging >pT3) according to treatment delay.  O-3 months - Ref.  >3 months – OR 0.74  Survival  Data not shown | P<0.001  Not reported | Reference  0.66  Not reported | Reference  0.82  Not reported | A treatment delay of >3 months was significantly associated with a lower rate of pathological upstaging. The rates of upstaging at radical prostatectomy decreased with increasing durations of treatment delay.  The results showed no difference in long term survival rates between the two groups. Increasing duration of treatment delay was not significantly associated with worse survival in adjusted analysis. | Not reported |  |
| Tokuda (2009) | Comparison of the mean symptom to visit intervals between patients with and without metastasis: (Student's t-test) | 55.4/101.2 | Non-significant |  |  | There was no significant difference in symptom to visit interval for patients between patients with and without metastasis with prostate cancer | Not undertaken |  |
| Tørring (2013) | Kaplan-Meier method, conditional logistic regression to estimate ﬁve-year mortality odds ratios as a function of the diagnostic interval using restricted cubic splines and adjusting for comorbidity, age, sex and type of cancer. | Mortality rates (MR%) and crude/adjusted odds ratios (OR) for the 1st and 4th compared with 2nd and 3rd diagnostic interval quartiles (DIQ) for patients with a) alarm or serious symptoms and b) vague symptoms.  Alarm or serious symptoms  DIQ MR% Crude OR Adjusted OR  1^st^ 62.1 2.45 2.35  2^nd^ + 3^rd^ 40.0 1 1  4^th^  50.0 2.46 1.42  Vague symptoms  DIQ MR% Crude OR Adjusted OR  1^st^ 63.2 2.22 2.93  2^nd^ + ^3rd^ 40.0 1 1  4^th^ 50.0 0.71 0.63 |  | 0.97/0.89  Reference  0.94/0.54  0.72/0.79  Reference  0.22/0.14 | 6.18/6.22  Reference  6.44/3.74  6.85/10.88  Reference  2.29/2.77 | In patients with alarm or any serious symptoms, mortality grew the longer the diagnostic intervals in those 40% from this group who experienced the longest delays. In patients with alarm or any serious symptoms, having very short diagnostic intervals was also associated with a high mortality.  Inversely, in patients presenting with vague symptoms, we saw much longer diagnostic intervals, the same survival probability and an opposite, concave trend between the length of the diagnostic interval and mortality. However, the latter association was not statistically signiﬁcant and the association did not apply to prostate cancer. The study displays the immense complexity and difficulty of diagnosing cancer. Yet, we can infer from the increasing trends in mortality that a few weeks can make a difference – that time matters. | Not reported |  |
| **Renal** | | | | | | | |  |
| Holmang (2006) | Hazard ratio for delay and disease specific survival        Median delay by tumour stage | Univariate analysis of hazard ratio for different delay types and disease-specific survival  Delay between 1.000 symptom and urography Delay between 1.002 urography and surgery Delay between 1.001 symptom and surgery  Multivariate analysis Delay not in model Ta  T1  T2  T3  T4 | p=0.4269  p= 0.0440  p=0.0779  88.5  101  78  76  56 | 0.998  1.000  1.000 | 1.001  1.005  1.002 | Univariate analysis revealed a short delay between urography and surgery was associated with short survival  Delay had no prognostic significance in the multivariate analysis  Overall delay for patients with ureteral and renal pelvis tumours had no significant impact on prognosis. The delay among patients with advanced renal pelvic and ureteral tumours was shorter than that for patients with non-invasive tumours | Not undertaken |  |
| Tokuda (2009) | Comparison of the mean symptom to visit intervals between patients with and without metastasis: (Student's t-test) | 64.9/95.8 | Non-significant |  |  | The risk for distant metastases is higher among patients with solid tumours and a short interval of symptom onset to clinical presentation. | Not undertaken |  |
| **Bladder** | | | | | | | |  |
| Gulliford (1991) | Association of total delay before treatment with unadjusted survival and survival adjusted for case severity (Proportional hazards model)  < 27 days =>27- =>48- => 84 days Association of hospital delay before treatment with unadjusted survival and survival adjusted for case severity (Proportional hazards model)  < 14 days =>14- =>26- => 51 days Association of duration of symptoms at referral with unadjusted survival and survival adjusted for case severity  (Proportional hazards model) < 2 weeks =>2- >8 weeks not known | Adj/UnAdjust  1.00/1.00 0.59/0.62  0.65/0.85 0.63/1.01  Adj/UnAdjust  1.00/1.00 0.51/0.60 0.60/0.68 0.59/1.02   Adj/UnAdjust  1.00/1.00 1.34/1.27 1.39/1.75 | Adjusted p=0.193 Unadjusted p=0.095     Unadjusted p=0.014  Adjusted p=0.085     Unadjusted p=0.249  Adjusted p=0.062 | Adj/Unadj  0.37/0.37 0.41/0.51 0.40/0.60  Adj/Unadj   0.32/0.37 0.60/0.43 0.39/0.63   Adj/Unadj  0.86/0.80 0.91/1.1 | Adj/Unadj  0.93/1.03 1.01/1.41 1.00/1.70  Adj/Unadj   0.79/0.98 0.92/1.09 0.91/1.65   Adj/Unadj  2.1/2.02 2.14/2.79 | After adjusting for severity there was little evidence to suggest that processes of care contributed greatly to the variation in survival of patients with cancer of the bladder.  Results suggest that patients with worst prognosis were selected for early treatment, and there was little evidence to suggest that the prognosis deteriorated with increasing delay.  There was no evidence that the overall prognosis was worse for the patients referred later as the unadjusted hazard rate was similar to that for patients referred for radiotherapy at initial assessment. | Not undertaken |  |
| Hollenbeck (2010) | Harms Ratio; Cox proportional hazards model. Comparison of interval between initial claim of haematuria and diagnosis (<3 months, <6 months, <9 months, 9-12 months) on mortality. | Harm Ratio Unadjusted/Adjusted:  <3 months = 1.0/1.0 3  <6 months = 1.09/1.00 6  <9 months = 1.19/1.16  9-12 months = 1.39/1.34 |  | 0.99/0.89  1.07/1.03  1.26/1.20 | 1.20/1.11  1.33/1.31  1.54/1.50 | Compared with patients who were diagnosed within 3 months, patients who had delays ≥9 months were 34% more likely to die from bladder cancer after adjusting for patient demographics. After incorporating disease severity measures into the model, the risk was attenuated by only 14.7%, suggesting that differences in tumour grade and disease stage explained little of the observed differences in bladder cancer mortality across diagnostic delay strata. | Not reported |  |
| Liedberg (2003) | Relation between tumour stage and total delay analysed by box plot.   Relation between tumour stage:  T1  T2-T4  and diagnostic delay studied using Cox regression analysis.  Diagnostic delay was split into four periods:  0-3 months  3-6 months  6-12 months  > 12months | Not reported   Not reported | p=0.02  p=0.12  p=0.001 | 0.84  0.23 | 4.7  0.69 | A significantly longer diagnostic delay was detected for more advanced tumour stages. | Among patients with T1 tumours those with a diagnostic delay of >6months showed a relative risk of bladder cancer death of 2.0. In the group with muscle-invasive tumours (T2-T4) there was a relative risk of bladder cancer death of 0.39 in the two groups with a longer diagnostic delay (>6 months) compared to patients with a shorter diagnostic delay. |  |
| Maguire (1994) | Comparison of median survival times (in months) by symptom to diagnosis (SDI) quintile groups  (Lee-Desu test) : Q1, Q2, Q3, Q4, Q5  Hazard ratio for patients in Q1 vs Q2 vs Q3 vs Q4 vs Q5 (log rank test) Comparison of median SDI for those with local vs regional vs disseminated disease (Kruskall Wallis Test) | Not reported   Not reported  Not reported | p=0.627    p=0.322   p=0.324 | n/a  n/a  n/a | n/a  n/a  n/a | The SSR for bladder cancer was low in comparison to the high value for stomach or lung cancer (two sites with the worst prognosis).  The duration of symptoms was found to bear a weak relationship with tumour stage and with survival. | Not undertaken |  |
| Mommsen (1983) | Four year crude survival rate for 100 patients with bladder cancer stage T1-T2 correlated with delay from onset of symptoms to definitive treatment  (Life table (actuarial) method) Delay: =<20 weeks 21-52 weeks >52 weeks | Not reported | Non-significant | n/a | n/a | Among the 100 patients with T1 or T2 tumour there was a tendency towards longer survival with short total delay. | Not undertaken |  |
| Tokuda (2009) | Comparison of the mean symptom to visit intervals between patients with and without metastasis: (Student's t-test) | 39.8/85.4 | Non-significant | n/a | n/a | The risk for distant metastases is higher among patients with solid tumours and a short interval of symptom onset to clinical presentation. | Not undertaken |  |
| Wallace (2002) | Stratified survival analysis to test for differences within delay times adjusting for tumour stage (Log rank test) Median survival by delay times stratified for tumour category (Log rank test) Total delay pTa =< 110 days  pTa >110 days  pT1 =< 110 days  pT1>110 days  T2-T4 =< 110 days  T2-T4>110 days  Median survival by total delay  =< 110 days >110 days Median survival by delay times stratified for tumour category (Log rank test) Hospital delay pTa =< 68 days  pTa >68 days  pT1 =< 68 days  pT1>68 days  T2-T4 =< 68 days  T2-T4>68 days Median survival by hospital delay  =< 68 days >68 days  Median survival by delay times stratified for tumour category (Log rank test) Delay 1 pTa =< 14 days  pTa >14 days  pT1 =< 14 days  pT1>14 days  T2-T4 =< 14 days  T2-T4>14 days Median survival by Delay 1  =< 14 days >14 days  Median survival by delay times stratified for tumour category (Log rank test) Delay 3 pTa =< 20 days  pTa >20 days  pT1 =< 20 days  pT1>20 days  T2-T4 =< 20 days  T2-T4>20 days Median survival by Delay 3  =< 20 days >20 days Median survival by delay times stratified for tumour category (Log rank test) Delay 2 pTa =< 28 days  pTa >28 days  pT1 =< 28 days  pT1>28 days  T2-T4 =< 28 days  T2-T4>28 days Median survival by Delay 2  =< 28 days >28 days | Not reported | p= 0.43  p=0.17  p=0.01  p=0.001  p=0.06         p=0.02  p=0.29  p=0.47   p=0.001         p=0.001 | 6.3 5.4 0.9 1.3  6.3 7.4    9.1  5.4 5.8 0.9 1.3  5.7 8.4    6.3 5.4 1.1 1.0  8.0 6.0  9.2  5.4 5.8 0.9 1.4  6.9 6.9  9.2  5.0 6.4 0.9 1.3  5.6 8.5 | 1.4 2.3  1.4 2.4  8.1    2.1 1.7   8.5    1.3 2.4  1.6 2.3  8.0 | The total delay had no effect on survival (p=0.17) this was also true after adjusting for tumour stage (p=0.43) Survival is significantly better for those patients with bladder cancer referred to hospital by their GPs within 14 days of the onset of symptoms.  Women, younger patients and non-smokers had a significantly longer total delay and may reflect a lower index of suspicion of bladder cancer in these groups of patients. The total delay had no significant effect on survival. However, with stage, patients with a shorter total delay had a worse survival except for patients with pT1 tumours where the reverse was true. | Not undertaken |  |
| **Testicular** | | | | | | | |  |
| Akdas (1986) | Comparison of mortality rates in patients with High-volume metastasis (HVM) among those with a symptom to diagnosis interval of < 4 months vs. ≥ 4 months (descriptive)  Comparison of remission rates among patients with symptom-chemotherapy interval of <=6 months vs. >6months (descriptive)  Comparison of symptom to diagnosis interval in patients with Stage III cancer with (descriptive):  < 4 months vs. ≥ 4 months (HVM)  < 4 months vs. ≥ 4 months (LVM) | 3/9 (33.3%) vs 7/15 (46.6%)  4/10 (40.0%) vs 5/19 (26.3%)  37.5% vs. 62.5%  20% vs. 80% | Not reported  Not reported  Not reported |  |  | A shorter symptom to diagnosis interval improved the complete remission of patients with stage III disease. Patients with high volume metastases (HVM) have a poorer prognosis than those with low volume metastases (LVM) | Not undertaken |  |
| Bosl 1981 | Median interval in days related to clinical stage.  Patient delay:  stage I  stage II  stage III  Difference between Stage I + II vs. Stage III (Mann-Whitney U test):  Physician delay:  stage I  stage II  stage III  Difference between stage I + II vs. stage III  (Mann-Whitney U test):  Patient plus physician delay:  stage I  stage II  stage III  Difference between stage I + II vs. stage III  (Mann Whitney U test): | 30 days (1.0 months)  38 days(1.25 months)  60 days (2.0 months)  9 days (0.3 months)  11 days(0.36 months)  12 days (0.4 months)  75 days(2.5 months)  101 days(3.3 months)  134 days(4.4 months) | p=0.034  p=0.071  p=0.017 | n/a | n/a | Extent of patient and total delay was positively related to advanced clinical stage of testicular cancer at diagnosis | Not undertaken |  |
| Chilvers 1989 | Comparison of proportion of patients with Stage I tumours among those with patient delay <=100 days (54%) vs. >100days(41%) (chi-square test):  Test for trend with delay by group* and relapse free survival from orchidectomy in patients with metastatic disease (log rank):  Test for trend with delay by group* and relapse free survival from orchidectomy in stage I marker negative tumours (log rank):  Association between patient delay and relapse in patients with metastatic disease and allowing for MRC prognostic group, stage and marker level (Cox regression)  * 0-49/50-99/100+/all | 3.79, d.f.=1  4.39, d.f.=1  0.55, d.f.=1  Not reported | p=0.05  p<0.05  p>0.1  p>0.05 | n/a | n/a | Of those who sought medical advice within 100 days of onset of symptoms, 54% had stage I tumours compared to 41% who delayed longer. Delay in seeking medical advice is inversely related to relapse. This could be due to faster growing tumours producing symptoms leading to medical consultation. However, after excluding stage I marker-negative patients and allowing for prognostic group, delay was a significant (p<0.05) indicator of relapse. | Not undertaken |  |
| Dieckmann 1987 | Correlation between the duration of symptoms and increasing tumour stage (method not reported)  Correlation between the extent of local tumour infiltration (Pt-stage) and duration of symptoms: Comparison of mean duration of symptoms among those who died from nonseminoma vs. mean duration of symptoms for all nonseminomas: | No correlation found  No correlation found  Not reported | Not reported | n/a | n/a | In both histological groups, symptomatic interval was longer for stage I than II. Longest symptomatic interval found in patients in nonseminoma group with stage III. Longer duration of symptoms has an unfavourable effect on the prognosis of nonseminoma patients. | Not undertaken |  |
| Fossa 1981 | Association between patient delay (< 2 vs. > 2 months) with stage and survival (descriptive).  Association between doctor delay (< 2 vs. > 2 months) with stage and survival (descriptive)  Association between duration of symptoms (< 2 vs. > 2 months) with stage and survival (descriptive) | Not reported | Non-significant | n/a | n/a | The different time intervals did not correlate with clinical stage or survival | Not undertaken |  |
| Hanson 1993 | Comparison of survival rates at 3 year among those with duration of symptoms:  <16 weeks  >16 weeks  (student t-test) | 1/15 (7%)  7/18 (39%) | p<0.01 | n/a | n/a | Significant correlation between duration of symptoms and survival | Not undertaken |  |
| Harding 1995 | Association between diagnostic delay* and presentation with local vs. metastatic, good prognosis vs metastatic, poor prognosis  (Chi square test)  Comparison of 5-year survival rate in categories of diagnostic delay*  (Cox model)  Comparison of 5-year survival rate in categories of diagnostic delay*  (Cox model)  * <1.5 months, 1.5 - 3 months, > 3 months | 1.61, 4 d.f.  87% (81-93) vs 78% (71-85) vs 78% (71-85)  Not reported | p=0.87  Not reported  Non-significant | n/a | n/a | Cox model showed that diagnostic delay was not an independent influence on survival. No apparent relationship between diagnostic delay and presentation with local, metastatic or advanced metastatic disease. | Not undertaken |  |
| Huyghe 2007 | Comparison of mean diagnostic delay among those with Stage I vs Stage II vs Stage III (extension of the Wilcoxon test):  Overall population  Non seminoma  Seminoma  Comparison of proportion of patients according to diagnostic delay category* (descriptive) in:  Stage I: 189 vs 31 30  Stage II: 90 vs 16 vs 15  Stage III: 37 vs 16 vs 15  Correlation of diagnostic delay with 5-year survival (Kaplan Meier, log rank):  Overall population  Non seminoma  Seminoma  * Diagnostic delay category: 1-3mo, 4-6mo, >6mo. | Not reported | p=0.003  p<0.001  Non-significant  p=0.001  p=0.001  Non-significant |  |  | Duration of diagnostic delay was significantly correlated with disease stage in the overall population and the nonseminoma group. It was not significant for seminoma. Duration of diagnostic delay had a significant impact on survival in the overall population. This effect was essentially due to the NSGCT group. | Not undertaken |  |
| Meffan 1991 | Association of length of presenting history and survival (log-rank):  Association of length of presenting history and advanced stage:  (Wilcoxon U paired test)  Correlation between length of history and size of tumour: (Pearson correlation coefficient) | Chi square=1.857  0.651 | p=0.173  p=0.808  0.001 |  |  | Increasing length of history was not associated with survival or more advanced stage of disease in either seminoma or NSGCT groups | Not undertaken |  |
| Moul 1990 | Association of survival and symptomatic interval (log rank test):  Seminoma 0-16 weeks vs  Seminoma > 16 weeks  Nonseminoma 0-8 weeks vs.  Nonseminoma 9-16 weeks vs.  Nonseminoma > 16 weeks  Trend in mean delay based on stage (t-test):  Seminoma  Non-seminoma | Not reported | p>0.18  p=0.002  Not reported |  |  | Delay did not significantly impact on survival in the seminoma group. Delay of greater than 16 weeks significantly impacted on survival in the nonseminoma group. There is a clear association between an increasing mean symptomatic interval and increased stage for the nonseminoma group | Not undertaken |  |
| MRC Working Party 1985 | Association of 3-year survival rate and length of history of:  < 3 months vs > 3 months  Unadjusted (Chi square, log rank test)  Adjusted for stage and marker status (Cox regression): | Chi-square=5.4, df=1  Chi-square=1.4, df=1 | p=0.02  p=0.23 |  |  | Patients with a history of greater than 3 months had a worse survival than those with a shorter history. A longer history is associated with more advanced disease. | Not undertaken |  |
| Napier 2000 | Association between duration of symptoms and relapse rate in those with symptom duration* of ≤ 4 weeks vs ≥ 4 months  (Mann-Whitney U test):  Correlation between duration of symptoms and time to relapse (Correlation analysis):  * Relapse Rate: ≤ 4 weeks (8/91) vs 1-3 months (12/64) vs  ≥ 4 months (6/30): | Not reported  r=0.2 | Non-significant  Non-significant |  |  | The results of this small study suggested a non-significant trend toward those patients presenting with a symptom duration ≤ 4 weeks having a lower relapse rate than those presenting after 4 weeks. Among those patients relapsing, there was no correlation between symptom duration and time to relapse | Not undertaken |  |
| Prout 1984 | Association between delay in diagnosis* and survival:  Association between delay in diagnosis* and extent of disease:  * Prompt orchiectomy refers to orchiectomy within one month of symptoms | Not reported | Not reported |  |  | Deaths from nonseminomatous germ cell tumours decreased dramatically (29% vs 12%) when diagnosis and treatment were carried out promptly. Those diagnosed within one month of symptoms had a higher tendency to metastasise as metastases were present in 21 of the 34 of these patients. | Not undertaken |  |
| Scher 1983 | Relationship between response to treatment and symptomatic interval:  3 months or less (48/81- 59%) vs at least 3 months (13/38 -34%) (Chi-square test)  Comparison of median symptomatic interval in those with palpable retroperitoneal disease (3.3 months) vs those with either nonpalpable disease or no retroperitoneal disease | Chi square = 6.5, df=2  Chi-square=5.46 d.f=2 | p=0.039  p=0.065 |  |  | There was a significant association between response to treatment and symptomatic interval. No statistically significant difference between number of metastatic sites of disease and symptomatic interval was demonstrated. | Not undertaken |  |
| Ware 1980 | Correlation between duration of symptoms (mean delay-weeks) and stage of tumour.  Embryonal cell carcinoma  Stage I,II,III  Teratoma, teatocarcinoma  Stage I,II,III  Seminoma  Stage I,II,III | 5.5/10.7/15.1  15.9/20.8/25.0  19.3/26.4/6.0 | Not reported | n/a | n/a | The length of the duration of symptoms strongly correlated with a higher stage of disease at the time of diagnosis and staging. | Not undertaken |  |
| Wishnow 1990 | Association between delay in orchiectomy* and stage:  Stage I  Stage II  Stage III  Marker only  Association between delay in orchiectomy* and survival:  Association between delay in orchiectomy* and proportion of cases without metastatic disease:  *Group I ( <= 1 month after the onset of symptoms, n=65) vs Group II ( > 1 month after the onset of symptoms, n=89) | 40 (62%) vs 25 (28%)  14 (22%) vs 15 (17%)  5 (8%) vs 35 (39%)  9 (9%) vs 14 (16%)  1 (1.5%) vs 11 (12.4%)  43% vs 19% | p<0.001  Non-significant  p<0.001  p=0.0072  p<0.001 |  |  | Clinical stage I disease was present more frequently when orchiectomy was performed within 30 days of the onset of symptoms whereas clinical stage III was present more frequently when orchiectomy was performed more than 30 days after the onset of symptoms. Prompt orchiectomy should reduce mortality. | Not undertaken |  |
| **Upper Tract Urothelial** | | | | | | | |  |
| Sundi (2012) | Kaplan-Meier, log rank test.  5-year cumulative disease-specific survival and overall survival and delay. | 5-year cumulative disease-specific survival in:  Early group: 71.6%  Delayed group: 70.6%  5-year cumulative overall survival in:  Early group: 59.5%  Delayed group: 69% | Non-significant  P=0.389 | 63  49  51  48 | 78.4  84  67  83 | Overall, there were no differences between the early and delayed surgery groups with respect to recurrence-free, disease-specific, and overall survival. | Overall survival of patients who did not receive neo adjuvant chemotherapy (n=207) who underwent early or delayed surgery: p=0.845. |  |
| Waldert (2010) | Chi Square test, Kruskal-Wallis test, Mann-Whitney U-test.  Kaplan-Meier method, log-rank test.  Stage: treatment delay and stage.  Survival: treatment delay and survival.  Recurrence: treatment delay and recurrence. | pT stage and treatment delay(categorical)of:  < 3 months/> 3 months  Ta:34 (23.3)/5 (2.2)  Tis: 6 (4.1)/2 (4.9)  T1: 42 (28.8)/8 (9.5)  T2: 25 (7.1)/7 (7.1)  T3: 38 (26.0)/16 (39.0)  T4: 1 (0.7)/3 (7.3)  pT stage and treatment delay (continuous, days). Median (IQR)  Ta: 32(59); Tis: 45(66); T1: 45(62); T2: 36(66); T3: 64(62); T4: 102(58)  Actuarial cancer specific survival (CSS)and treatment delay:  3 years: 70 (4%)  5 years: 69 (4%)  Actuarial recurrence free probabilities after RNU and treatment delay:  3 years: 69 (4%)  5 years: 65 (4%)  Univariate cox regression:  Recurrence HR 1.00.  CSS HR 1.00 | P=0.044  P=0.026  P=0.0637  P=0.658 | 0.99  0.99 | 1.00  1.01 | Time from diagnosis to RNU as a categorical variable (threshold 3 months) increased with advancing pathological stage.  Kaplan-Meier analyses showed no statistical difference in the risk of disease recurrence or cancer-specific mortality between patients who had RNU at <3 months or at >3 months after diagnosis. | Sub group of 90 patients who had muscle invasive disease on RNU. The disease recurred in 44 of 90 patients and 57 were dead at the time of analysis and 40 dies from UTC. Kaplan-Meier analyses showed no statistical difference in the risk of disease recurrence or cancer-specific mortality between patients who had RNU at <3 months or at >3 months after diagnosis |  |
| **Gynaecological cancers** | | | | | | | |  |
| **Cervical** | | | | | | | |  |
| Fruchter (1981) | Chi square test Patient delay >3 months Clinical staging  Pathologic staging Doctor delay >3 months Clinical staging  Pathologic staging Total delay >6 months Clinical staging  Pathologic staging | n/a | <0.013 <0.003  Non-significant Non-significant  <0.04 <0.05 | n/a | n/a | The length of patient delays in diagnosis was positively associated with stage of disease. The length of doctor delay was not associated with stage of disease | Not undertaken |  |
| Tokuda (2009) | Comparison of the mean symptom to visit intervals (days) between patients with and without metastasis: (Student's t-test) | 90.2/82.6 days | Non-significant | n/a | n/a | The risk for distant metastasis is higher among patients with solid tumours and a short interval of symptom onset to clinical presentation. | Not undertaken |  |
| Umezu (2012) | Kaplan-Meier method; log-rank test. Impact of waiting times to the operation on 5-year recurrence-free survival (RFS) and overall survival (OS). | Overall 5 year survival and wait time to operation of:  < 50 days: 80.9%  > 50 days: 92.5%  5 year recurrence free survival and wait time of:  < 50 days: 91.4%  > 50 days: 96.7% | P=.653  P=.106 | Not reported | Not reported | The waiting time to the operation was not a significant variable associated with a poorer RFS and OS. The therapeutic delay did not influence survival. | Not reported |  |
| **Endometrial** | | | | | | | |  |
| Crawford (2002) | Hazard ratio for those with interval from GP referral to treatment: | Multivariate results: 40-61 days: 0.71 62-91 days: 0.47 > 92 days: 0.53 | n/a | 0.43 0.27 0.30 | 1.19 0.83 0.93 | ...we found that patients who experience the longest delay in treatment are more likely to survive | Not undertaken |  |
| Elit (2013) | Hazard ratios and impact of wait times grouped into categories in weeks (0 to 2.0, 2.1 to 6.0, 6.1 to 12.0, >12) on overall survival. (Kaplan-Meier method; univariate Cox proportional hazards regression; multivariable Cox model) | Hazard Ratio Wait time (weeks)  Univariate analyses  Multivariate analyses | P<.001  P<.001 |  |  | After adjusting for all factors in the multivariate model, wait time remained a highly significant prognostic variable for overall survival. | Not reported |  |
| Franceschi (1983) | Chi-square test for trend (duration of symptoms compared to stage)  Proportion of participants with advanced tumour stage among those with bleeding time < 6 months vs > 6 months (Test for trend using the test given by Mantel) | 4.0 | < 0.05 | n/a | n/a | Among women with longer lag time, there was an increased frequency of more advanced tumor stages compared to women whose first bleeding occurred less than six months before | Depth of myometrial invasion, histologic differentiation |  |
| Fruchter (1981) | Chi-square test for: patient delay > 3 months by stage Clinical staging Pathologic Total delay > 6 months by stage Clinical stage Pathologic-clinical | n/a | < 0.03  < 0.001  > 0.05 <0.01 | n/a | n/a | The trend to longer patient delays at higher stages, especially with pathologic staging, was also true for carcinoma of the endometrium. | Not undertaken |  |
| Menczer (1995) | Association between delay in diagnosis and survival, analysed separately as a continuous and categorical variable (log-rank test) | Not reported | Univariate analysis: > 0.15 Multivariate analysis: > 0.3 | n/a | n/a | We found no significant correlation of delay in diagnosis or treatment with the prognostic factors or with survival. | Analysis with and without myometrial invasion. Analysis stratified by stage (showed significance) |  |
| Obermair (1996) | Chi-square test of proportion of FIGO stage IA, IB in patients with bleeding interval:  < 4 weeks  4- 8 weeks  > 8 weeks Comparison of mean bleeding time (weeks) in Stage IA, IB vs. IC-IV (non-parametric t-test) | 10.358  23 (88.5%)  22 (64.7%)  29 (51.8%)  (12.7, SD 17.8) (35.2, SD 69.3) | 0.006    0.011 | n/a | n/a | ...that advanced disease in some cases might be caused by delayed diagnosis in women with poor compliance. | In 63 women undergoing pelvic lymphadenectomy, lymph node involvement was detected in 6 cases. The bleeding interval in patients with negative and positive nodes showed no statistically significant difference. |  |
| Pirog (1997) | Distribution of tumour stage among those diagnosed from onset of symptoms (bleeding) of <1month:  Stage I  Stage II-IV  > 1month:  Stage I  Stage II-IV  < 6 months:  Stage I  Stage II-IV  > 6 months:  Stage I  Stage II-IV  (Fisher's exact test) | 10 (71%)  4 (29%)  111 (73%)  40 (27%)  87 (73%)  32 (27%)  34 (74%)  12 (26%) | 0.87  0.92 | n/a | n/a | The data presented in this paper failed to demonstrate any correlation between time interval from onset of bleeding to definitive therapy, and stage of EMC | Duration of bleeding analysed both as a continuous and a categorical variable. No correlation between duration of symptoms and myometrial invasion, when stratified based on tumour grade (r2= 0.001) |  |
| Tokuda (2009) | Comparison of the mean symptom to visit intervals (days) between patients with and without metastasis: (Student's t-test) | 49.4/110.4 days | Non-significant | n/a | n/a | The risk for distant metastasis is higher among patients with solid tumours and a short interval of symptom onset to clinical presentation. | Not undertaken |  |
| Robinson (2012) | Associations between the QoL scales, patient satisfaction and total delay, Poisson regression modelling. | Total delay; P (crude)/P (adjusted for age stage, in current chemotherapy treatment and type of treatment received)  Overall QoL  Patient satisfaction. | p=0.01/p<0.01  p<0.01/p<0.01 | Not reported | Not reported | For women diagnosed with endometrial cancer, longer total delay was associated with reduced overall QoL. With regard to patient satisfaction, longer total delay and decreased patient satisfaction with the diagnostic phase were statistically significantly associated for women diagnosed with ovarian and endometrial cancer. This association also remained statistically significant when adjusted for age, cancer stage, treatment received and current chemotherapy treatment. | Not reported |  |
| **Ovarian** | | | | | | | |  |
| Fruchter (1981) | Chi square test Total delay Patient delay Doctor delay | 0.14 0.45 0.40 | Non-significant | n/a | n/a | Delay in diagnosis was not associated with stage of disease. | Not undertaken |  |
| Lurie (2010) | Association of symptom duration with stage at diagnosis | Symptom duration by stage N (%) Mean (SE):  Stages I-II: 329 (53) 13.7 (1.5)  Stages III-IV: 293 (47) 7.2 (1.7) | P=0.03 | Not reported | Not reported | Significant interaction of stage (early vs advanced) effects on symptom duration was observed. | Not reported |  |
| Menczer (2009) | Association of median symptom duration in months (range) and stage: Stage I Stage II Stage III Stage IV (Kruskal-Wallis) Duration of symptoms of ≤ 2 months, > 2 months as a categorical variable | 3 (1-36) 3 (1-36) 2 (1-36) 3 (1-12)  Data not presented in full | p=0.26     p=0.5 | n/a | n/a | No statistically significant association was found between median duration of symptom and stage      No difference in the percentage of patients with symptom duration greater than 2 months was observed by stage. | Not undertaken |  |
| Nagle (2011) | Impact of time delay on stage presentation; borderline; FIGO stages I to II and FIGO stages III to IV. Log-rank test.  Survival:  Association of symptom duration on survival. Cox regression to obtain hazard ratios. | Reported graphically, not in text.  Symptom onset to presentation:  ≤ 1 month HR 1.00  2-3 months HR 0.97  4-6 months HR 0.92  7-12 months HR 0.89  >12 months HR 0.70  Presentation to diagnosis  ≤ 1 month HR 1.00  2-3 months HR 1.01  4-6 months HR 0.85  7-12 months HR 1.01  >12 months HR 1.09  Symptom onset to diagnosis  ≤ 1 month HR 1.00  2-3 months HR 0.94  4-6 months HR 0.88  7-12 months HR 0.86  >12 months HR 0.94 | Non-significant | Not reported  1.00  0.75  0.64  0.58  0.39  1.00  0.78  0.62  0.72  0.73  1.00  0.74  0.68  0.65  0.68 | Not reported  1.26  1.32  1.35  1.26  1.31  1.17  1.43  1.63  1.18  1.13  1.14  1.30 | The time from (a) first recalled symptom to the date of first medical practitioner consultation, (b) first recalled symptom to date of clinical diagnosis, and (c) first medical practitioner consultation to clinical diagnosis, were not significantly different among women with borderline, early and late stage disease.  Time from first symptom onset to first medical practitioner consultation was not associated with survival. | Not reported |  |
| Neal (2007) | Comparison of interval data Chi squared and t-tests Referral delay Secondary care delay Comparison of referral route and stage  Survival comparison by referral route Kaplan-Meier  log rank test | 0.99 2.94 2.24  Degrees of freedom (df) =3 459.9 (SE 110.4), df = 1 | p <0.001 p =0.021 p =0.524   p = 0.209 | 243.6 | 676.4 | There was no difference in survival rates between urgent guideline referrals (and all referrals marked as urgent) and those diagnosed through other routes.    Comparison of all urgent referrals with other routes showed a borderline difference. | Comparison of all urgent referrals (guideline and letter) with other routes and stage also showed no difference (p=0.510)  Comparison of all urgent referrals (guideline and letter) with other routes and survival also showed no difference (p=0.076) |  |
| Robinson (2012) | Associations between the QoL scales, patient satisfaction and total delay, Poisson regression modelling. | Total delay; P (crude)/P (adjusted for age stage, in current chemotherapy treatment and type of treatment received)  Overall QoL  Patient satisfaction. | p=0.09/p=0.04  p<0.01/p<0.01 | Not reported | Not reported | For women diagnosed with ovarian cancer, longer total delay was not associated with reduced QoL in the crude analyses. However, when analyses were adjusted for age, cancer stage, treatment type received and current chemotherapy treatment, longer total delay was associated with reduced overall QoL. With regard to patient satisfaction, longer total delay and decreased patient satisfaction with the diagnostic phase were statistically significantly associated for women diagnosed with ovarian and endometrial cancer. This association also remained statistically significant when adjusted for age, cancer stage, treatment received and current chemotherapy treatment. | Not reported |  |
| Smith (1985) | Two tailed significance test To test association between stage and delay | Not reported | Non-significant | n/a | n/a | There was no association between delay, perceived cause, or seriousness of symptoms with stage of disease at diagnosis. | Not undertaken |  |
| Tokuda (2009) | Comparison of the mean symptom to visit intervals between patients with and without metastasis:(Student's t-test) | 13.9/50.5 | p<0.01 |  |  | The risk for distant metastasis is higher among patients with solid tumours and a short interval of symptom onset to clinical presentation. | Not undertaken |  |
| **Head and Neck** | | | | | | | |  |
| Alho (2006) | Association between outcome of initial primary care visit (referred or followed up versus overlooked patients) and disease specific death at 3 years was analysed using Survival curves by Kaplan–Meier method, log rank test for significance and Cox proportional-hazards regression model for the multivariate analysis (Adjusted for age, sex, socioeconomic status, duration of symptoms, cancer stage and study period) Tongue or glottic carcinomas Referred or followed up (n)= 98 Overlooked (n)= 24  Pharyngeal or sub/supra glottic carcinomas Referred or followed up (n)= 78 Overlooked (n)= 21  All head and neck carcinoma patients Referred (n) Followed up (n) Overlooked (n) | Patient deaths n(%) 12 (13%) 11 (46%) Adjusted HR 4.25 Patient deaths n(%) 39 (54%) 9 (44%) Adjusted HR 1.15 Deaths at 3 years 31% 30% 45% Absolute increase in risk of death between referred and overlooked patients at 3 years -14% Absolute increase in risk of death between referred and followed up patients -1% | Not reported    Not reported  p=0.027  p=0.97 | 1.59    0.49  23% 17% 30%  –3%  –16% | 11.4    2.68  39% 43% 60%  31%   14% | The only background factors associated with the disease being overlooked were the patient’s low socioeconomic status, short duration of symptoms (patient delay) and insufficient physical examination. Patients whose disease was overlooked had a significantly shorter time from diagnosis to disease-specific death than patients who were referred or followed up in tongue or glottic carcinomas  In a sensitivity analysis, both the calculation of tumour-specific survival from symptom onset and the use of all-cause death as the end point diminished the effect only slightly. | Not undertaken |  |
| Allison (1998) | Univariate analysis: Stepwise multiple logistic regression Upper aerodigestive tract cancers Patient delay   Professional delay   Total delay     Mouth Patient delay   Professional delay   Pharynx Patient delay  Professional delay   Larynx Patient delay   Professional delay  Multifactorial model (OR) | Odds ratio (OR) of late versus early stage disease < 1 month 1.00  1-3 months 1.57 > 3 months 1.44 < 1 month 1.00 1-3 months 1.56 > 3 months 3.16 < 1 month 1.00 1-3 months 2.76 4-6 months 2.76 7-12 months 3.98 > 12 months 2.81  < 1 month 1.00 1-3 months 1.76 > 3 months 1.67 < 1 month 1.00 1-3 months 4.40 > 3 months 9.20  < 1 month 1.00 1-3 months 4.57 > 3 months oo < 1 month 1.00 1-3 months 0.86 > 3 months 1.18  < 1 month 1.00 1-3 months 0.66 > 3 months 1.90 < 1 month 1.00 1-3 months 2.35 > 3 months 2.08  Professional delay >1 month 2.28 | p=0.03   p=0.022        p=0.0001 | 0.74 0.68 0.68 Ref 0.75 1.29 Ref 1.08 1.14 1.46 0.97  Ref 0.47 0.43 Ref. 1.07 2.11  Ref 0.5  Ref 0.14 0.21  Ref 0.13 0.54 Ref 0.71 0.26  1.13 | 3.31 3.03 3.03  3.27 7.73  7.03 6.69 10.87 8.18  6.54 6.49  15.96 40.17   41.86   5.11 6.76   3.34 I .91  7.75 0.26-16.76  4.64 | Patient delay did not predict stage, but that professional delay greater than 1 month predicted an increased risk of late stage disease. Professional delay > 1 month has approximately twice the odds for late stage of professional delay < 1 month. A total delay > 12 months also has an increased risk of late stage disease compared with those whose total delay was < 1 month | Not undertaken |  |
| Al-Rajhi (2009) | Chi-squared test  Odds ratios (OR) and confidence intervals (CI) using logistic regression for early vs late stage disease | Patient delay < 2 months OR=1.0a ≥ 2 months OR=0.78 < 3 months OR=1.0a ≥ 3 months OR=1.02 Medical diagnosis delay < 2 months OR=1.0a ≥ 2 months OR=0.68 < 3 months OR=1.0a ≥ 3 months OR=0.46 Professional delay < 2 months OR=1.0a ≥ 2 months OR=0.70 < 3 months OR=1.0a ≥ 3 months OR=0.56 Total delay < 2 months OR=1.0a ≥ 2 months OR=0.45 < 3 months OR=1.0a ≥ 3 months OR=0.39 | p=0.40  p=0.92   p=0.12  p=0.007   p=0.26  p=0.04   p=0.16  p=0.01 | 0.45  0.58   0.38  0.26   0.37  0.32   0.14  0.18 | 1.38  1.84   1.20  0.82   1.32  0.98   1.40  0.83 | There was a significant association between a time delay of 3 months or more (diagnostic, professional and overall) and presentation with advanced stage tumour (III or IV). However patient-related delay time was not associated with stage | Not undertaken |  |
| Brouha (2000) | Hazard ratios calculated from the exponent of the coefficient (beta) Actuarial method of Kaplan and Meier for survival curves Inter group comparisons using the log- rank test Cox proportional hazards regression analysis for recurrence used for multivariate analysis adjusting for age, sex, smoking habits before radiotherapy, alcohol intake, and duration of symptoms | Duration of symptoms (=< 6 months versus => 6 months) Coefficient (beta) -0.4125, SEM 0.269, HR 0.662 | p=0.1253 | n/a | n/a | Duration of symptoms was not found to have statistically significant effects on recurrence of tumours (5 year recurrence free survival) | Not undertaken |  |
| Brouha (2005)a | Chi-square test to categorical data Kruskal-Wallis test to continuous data | Larynx (Total patient delay), x2 3.29  Glottic (Appraisal delay), x2 1.08  Nonglottic (Appraisal delay), x2 1.34 | p=0.07  p=0.30  p=0.25 | n/a | n/a | There was no significant difference in the length of patient delay between early and advanced disease There was no significant difference in median total delay between T1 to T2 tumors and T3 to T4 tumors (Table 3). Taking into account the involvement of neck node metastases, the median total delay was also not significant for small (stage I–II) tumors compared with advanced (stage III–IV) disease (9 weeks vs 5 weeks). | Not undertaken |  |
| Brouha (2005)b | Chi-square test to categorical data Kruskal-Wallis test to continuous data Odds ratios and 95% confidence intervals by logistic regression analysis | Pharyngeal cancers (<30 and =>30 days)  T Classification  T1-T2 OR= 1.00  T3-T4 OR= 4.5  N Classification  N0 OR= 1.00  =>1 OR= 0.6  Stage  I-II OR= 1.00  III-IV OR= 1.1  Oral cancers (<30 and =>30)  T Classification  T1-T2 OR= 1.00  T3-T4 OR= 3.2  N Classification  N0 OR= 1.00  =>1 OR= 1.4  Stage  I-II OR= 1.00  III-IV OR= 2.1  Oral OR = 3.2 | p=0.01   p=0.39   p=0.90    p=0.01   p=0.36  p=0.04 | 1.4   0.2   0.3    1.4   0.7  1.0 | 14.5   1.9   4.6    6.9   3.1  4.2 | Prolonged patient delay was associated with late-stage disease for both patients with pharyngeal cancer and patients with oral cancer | Not undertaken |  |
| Caudell (2011) | The DTI was tested as a continuous variable as well as categorized at the median or by quartiles. (Kaplan-Meier survival analyses; Regression analysis; Multivariate Cox regression analysis) (Odds Ratio) | Survival: On regression analysis, the DTI as a continuous variable was associated with overall survival (OR = 1.003)  However, Cox regression analysis did not show significant association (OR = 1.002)  Locoregional control and metastasis free survival: On regression analysis, the DTI as a continuous variable was not associated with  LRC (OR = 1.003)  DMFS (OR = 1.000). | P=.03  P=.26  P=.10  P=.97 | 1.000  0.999  0.999  0.992 | 1.006  1.005  1.007  1.008 | Previous studies suggest that many patients with head and neck cancer may present at a time of rapid growth, and delays could increase the target volumes and potentially diminish locoregional control. However, the results of the present study did not confirm the hypothesis, although there were trends suggesting that prolonging the DTI could be detrimental. Prior studies of DTI focused only on early-stage head and neck cancer, the present study included only LAHNC. Potentially in this population of relatively larger tumours, the growth fraction could be lower or the cell loss factor could be higher, resulting in a comparatively longer population doubling time and a reduced sensitivity to delay. | Not reported |  |
| Hansen (2005) | Median of symptoms duration was used as a cut off and duration below this was regarded as short and above as long. Kaplan-Meier analysis, and survival differences were analysed using the log–rank test. Cox multivariate analyses using local recurrence-free survival | Duration of symptoms RR (relative hazard ratio) 1.045 | p<0.0001 | 1.023 | 1.069 | The recurrence-free survival was significantly poorer for patients with duration of symptoms longer than the median value compared with patients with a shorter duration of symptoms One month of delay from onset of symptoms to start of radiotherapy was equivalent to a 4.5% decrease in recurrence-free survival | Not undertaken |  |
| Ho (2004) | Univariate Regression Analysis for Prolonged Presentation-to-Diagnosis Interval (>3 Months): T3 + T4 Stage IV  Univariate Hazard Ratios for Survival   Multivariate Hazard Ratios for Survival (PDI was not in the model) | OR 0.85 OR 2.11  PDI >=3 months 1.27 PDI >=6 months 1.04 PDI >=12 months 0.67 Stage IV 2.45 | p=0.75 p=0.13  p=0.52 p=0.93 p=0.59 p=0.05 | 0.33 0.80  0.62 0.40 0.16 0.99 | 2.25 5.55  2.60 2.73 2.82 6.02 | A PDI of 3 months or longer did not achieve a statistically significant correlation with survival on univariate analysis. There is a trend for patients with an extended PDI to be diagnosed with stage IV disease at the time of diagnosis but the association is not statistically significant | Not undertaken |  |
| Koivunen (2001) | Adjusted Relative Hazard of death for Patient and Physician delays (2-month threshold) | Patient delay  <2 months 1.0  =>2 months 2.50  Professional delay  <2 months 1.0  =>2 months 0.67  Advanced stage (International Union Against Cancer [UICC] TNM; Stage IV vs. Stage I–III; HR, 3.19; CI, 1.61– 6.35) and age ($ 65 vs. , 65 years; HR, 2.47; CI, 1.32– 4.62) also were associated with an impaired prognosis. The median patient delay was 1 month at all the sites, and altogether 33 (39%) of the patients had over 2 months of patient delay (Table 3). More than 2 months’ patient delay was not related to Stage IV disease at any of the tumor sites (P 5 0.09 for oropharynx, P 5 0.74 for nasopharynx, and P 5 0.13 for hypopharynx) | p=0.002    p=0.21 | 1.39    0.82 | 4.38    3.97 | Survival: The patients who had less than 2 months’ patient delay had significantly better survival than those with a patient delay of 2 months or more. Professional delay showed no differences between the groups regarding the effect on survival. A long patient delay was strongly related to a poorer prognosis. Overall, the pharyngeal cancer patients who had a patient delay for 2 months or more had a 2.5-fold risk for disease specific death. The impact of delay on survival was equally marked as that of Stage IV. There was no association between the stage of the disease and patient delay. Professional delay of more than 2 months was not associated with a poorer prognosis or with stage of the disease or histopathologic diagnosis in the patients with pharyngeal cancer in this series | Not undertaken |  |
| Kumar (2001) | Univariate regression analysis Primary delay versus advanced stage Secondary delay versus advanced stage | G-test 6.3 G-test 0.22 | p<0.0121 p=0.57 |  |  | Cancer stage at presentation is significantly related to primary and not secondary delay. The coefficient and constant of logistic regression showed that the longer the duration of primary delay (PD), the more the probability of having an advanced stage of cancer | Not undertaken |  |
| Lee (1997) | Logistic regression the association between stage category (I-II versus III-V) and symptom duration, adjusted for other covariates (OR) The 10-year actuarial disease-specific survival in in early versus late presenters Multivariate analysis of duration of symptoms and disease-specific deaths using the Cox regression model (HR) Univariate analysis, analysis of duration of symptoms and disease-specific deaths (HR) | 0.981  1.003  1.011 | p<0.001       p=0.142  p<0.001 | 0.972       0.999  1.007 | 0.990       1.008  1.016 | Symptom duration was a significant factor associated with stage category; the odds ratio for presenting with Stage I-II disease was 0.981 per month's delay in diagnosis. The 10-year actuarial disease-specific survival was significantly higher in early presenters | Not undertaken |  |
| McGurk (2005) | Fisher’s exact test  Multiple regression analysis and survival curves generated by the Kaplan—Meier method | Delay vs stage at presentation Retrospective series Prospective series Delay vs survival Retrospective series Prospective series | p=0.8 p=0.8  p=0.63 p=0.8 | n/a | n/a | Delay correlates with neither the stage of tumour at presentation nor survival. The most likely explanation is that some tumours are relatively silent until they are advanced | Not undertaken |  |
| Miziara (1998) | P values calculated from the data presented using Chi squared test, not reported by the authors | Diagnostic delay (<2 months, 3 months, 4-6 months, =>7 months):  T stage (T1, T2, T3, T4)  N stage (N0, N+)  Early stage (Stages I,II) vs advanced stage (Stage III,IV) Diagnostic delay (Early=<3 months vs Late=> 4 months):  T stage (T1, T2, T3, T4)  N stage (N0, N+)  Early stage (Stages I,II) vs advanced stage (Stage III,IV) | p=0.143 p=0.618  p=0.089   p=0.917 p=0.319  p=0.905 | n/a | n/a | There is no association between diagnostic delay and the extension of tumour or nodal involvement, independently of tumour localisation | Not undertaken |  |
| Pitchers (2006) | Spearman’s rank correlation and PoLytomous universal model (PLUM) ordinal regression for any association between delay in referral and stage at presentation, two tailed p value Survival assessed using Kaplan–Meier survival analysis, and the log-rank test (6 weeks or less, compared to greater than 6 weeks) | Correlation coefficient +0.309     x2 4.579 df 1 | p=0.011     p=0.032 | n/a | n/a | There is a positive relationship (not necessarily linear) between increased delay in referral and more advanced stage at presentation. From the data, it can be inferred that for every 1-week increase in delay in referral, it is estimated that the stage at presentation will progress by 0.045 of ‘a stage’ The group with less than 6 weeks delay had significantly better survival | Not undertaken |  |
| Scott (2005) | Univariate logistic regression analyses used to calculate the odds ratio of early versus advanced stage of disease due to diagnostic delay. Only factors significantly related to stage of disease were entered into a multiple logistic regression model | Early stage/advanced stage No delay (<3 months) OR 1.00 Ref. Delay (>3 months) OR 1.34 | Non-significant  p value not reported | 0.808 | 2.226 | The idiosyncratic relationship between stage of disease at diagnosis and duration of diagnostic delay has led researchers to conclude some patients have a more aggressive form of oral cancer and as such scope for earlier diagnosis is limited. Differences in tumour biology are not a likely explanation for the absence of a delay–stage relationship but instead, a proportion of tumours (approximately 27%) may be silent until advanced in stage | Not undertaken |  |
| Seoane (2010) | Comparison of survivor and exitus groups based on diagnostic delay. Kaplan-Meier, Chi-square; Odds Ratio. (Clinically relevant or those significantly relevant variables related to mortality were examined with Cox regression model). | Diagnostic delay: median/SD.  Survivor  ≤1.5 months: 16/59.3%  >1.5 months: 11/40.7%  Exitus  ≤1.5 months: 23/63.9%  >1.5 months: 13/36.1%  OR: 0.82 | P=0.71 | 0.29 | 2.29 | Diagnostic delay did not seem to influence survival to oral cancer in a significant way. Even when tumours with higher and lower proliferative activity were analysed individually, diagnostic delay did not influence survival. | Not reported |  |
| Sidler (2010) | Kaplan-Meier to plot outcome; generalized Wilcoxon test (Gehan method).  Comparison of time to treatment after diagnosis and its impact on survival. (Entire population: 91%/77%/58%). | Survival of 1/3/5 years and delay of <10 weeks:  First therapy session (30 patients; 81%):  93%/85%/ 62%.  Survival of 1/3/5 years and delay of >10 weeks:  First therapy session (4 patients;12%): 75%/25%/25% | P<0.05 | Not reported | Not reported | Factors with favourable prognostic value were concomitant radio chemotherapy regimens, photon radiotherapy, and a delay between diagnosis and first therapy session of less than ten weeks, respectively. | Not reported |  |
| Sheng (2008) | Chi-square test Extent of disease Degree of invasion (T1, T2, T3, T4) vs number of delays (short/intermediate/long) T1 vs 37/15/7 T2 vs 35/18/19 T3 vs 28/12/11 T4 vs 11/8/15 Nodal status (N0, N1, N2, N3) vs number of delays (short/intermediate/long) N0 vs 32/12/15 N1 vs 53/22/20 N2 vs 20/14/14 N3 vs 6/5/3 Clinical stages (I,II,III,IV) vs number of delays (short/intermediate/long) I vs 13/2/2 II vs 42/15/10 III vs 35/20/21 IV vs 21/16/19 | X2=13.78      X2=4.12      X2=14.05 | p<0.05      p>0.05      p<0.05 | n/a | n/a | Delay in diagnosis correlates with the degree of invasion of nasopharyngeal carcinoma. There was no significant difference in the nodal status among the three delay groups. A strongly significant correlation was found between the nasopharyngeal carcinoma stages and patient related delayed diagnosis. | Not undertaken |  |
| Teppo (2003) | Multivariate analysis stepwise Cox regression model with disease-specific survival Disease-specific survival Kaplan-Meier method and compared using Breslow’s test  Patient delay  <3 months  =>3 months  Professional delay  <6 months  =>6 < 12 months  =>12 months  Glottic tumours  Supraglottic tumors  Stage  I–III  IV | HR 1.0 HR 1.73  HR 1.0 HR 1.88 HR 4.74    HR 1.0 HR 5.18 | p=0.41   p=0.05  p=0.28 p=0.21   p=0.02 | 0.48   0.47 1.30     1.26 | 6.25   7.44 17.3     21.3 | Patient delay of 3 months or more was not related to overall survival or to survival glottic or supraglottic tumors  Professional delay was significantly associated with survival: longer delay implicated poorer survival | Not undertaken |  |
| Teppo (2005) | Chi-square test for categorical and the Kruskal–Wallis H-test for continuous variables. Total delay (months) No Recurrence (n = 43) Local Recurrence (n = 10) Neck Recurrence (n = 7) Distant recurrence (n = 6) Patient delay (months) No Recurrence (n = 43) Local Recurrence (n = 10) Neck Recurrence (n = 7) Distant recurrence (n = 6) Professional delay (months) No Recurrence (n = 43) Local Recurrence (n = 10) Neck Recurrence (n = 7) Distant recurrence (n = 6) Multivariate analyses with the stepwise Cox regression model Patient delay (<3 months vs=>3 months)  Local recurrence Neck recurrence Distant recurrence Professional delay (<12 months vs=>12 months) Local recurrence Neck recurrence Distant recurrence Kaplan–Meier survival function method and the log rank test for risk of recurrence with the disease-free time period as the outcome measure Professional delay (<12 months vs=>12 months) Local recurrence Neck recurrence | Median (range) 6 (2–69) 12 (2–79) 14 (4–30) 6 (2–17) Median (range) 2 (<1–60) 1 (<1–6) 1 (<1–6) 3 (<1–6) Median (range) 2 (<1–26) 10 (1–75) 10 (2–23) 2 (<1–16)   Adjusted relative HR 0.62 1.46 1.61 Adjusted relative HR 4.62 9.50 3.67 | p=0.25     p=0.56  p=0.007        p=0.49 p=0.68 p=0.59  p=0.02 p=0.015 p=0.32     p=0.019 p=0.019 | 0.17 0.24 0.28  1.25 1.55 0.28 | 2.36 8.81 9.10  17.1 58.3 48.7 | Prolonged professional diagnostic delay increased the risk of local and neck recurrence significantly in laryngeal carcinoma. The effect of longer professional diagnostic delay on the risk of recurrence was especially obvious among LSCC patients with early stage disease (T1–2 N0 M0) | Not undertaken |  |
| Teppo (2008) | Death rates (percentages) for 2 and 5 years (starting from the date of histologic diagnosis) with the Kaplan–Meier method and statistical significance using log rank test The independent effect of those diagnostic delays found to be significant in univariate analyses were further evaluated with Cox multivariate model adjusting for age (= 65 years), sex, tumour subsite and clinical stage (I–III versus IV, I–II versus III–IV and all stages separately).                        Multivariate analysis Professional delay of =>6 months resulted in an HR of 3.5 (95% CI 1.8–6.9) in laryngeal cancer as compared to the delay of <6 months | Patient delay  Tongue  <2 weeks vs >=2 weeks  At 2 years (50/23)  At 5 years (50/31)  <2 months vs >=2 months  At 2 years (31/23)  At 5 years (41/27)  <3 months >=3 months  At 2 years (29/25)  At 5 years (36/30)  Pharynx  <2 weeks vs >=2 weeks  At 2 years (50/65)  At 5 years (71/ 83)  <2 months vs >=2 months  At 2 years (54/72)  At 5 years (70/93)  <3 months >=3 months  At 2 years (63/61)  At 5 years (75/94)  Larynx  <2 weeks vs >=2 weeks  At 2 years (31/25)  At 5 years (39/40)  <2 months vs >=2 months  At 2 years (25/27)  At 5 years (32/47)  <3 months >=3 months  At 2 years (22/31)  At 5 years (30/54)  Professional delay  Tongue  <2 weeks vs >=2 weeks  At 2 years (37/23)  At 5 years (42/30)  <3 months vs >=3 months  At 2 years (27/30)  At 5 years (33/40)  <6 months >=6 months  At 2 years (25/50)  At 5 years (42/50)  Pharynx  <2 weeks vs >=2 weeks  At 2 years (75/59)  At 5 years (100/76)  <3 months vs >=3 months  At 2 years (67/36)  At 5 years (82/73)  <6 months >=6 months  At 2 years (65/25)  At 5 years (82/50)  Larynx  <2 weeks vs >=2 weeks  At 2 years (17/26)  At 5 years (33/40)  <3 months vs >=3 months  At 2 years (18/39)  At 5 years (32/53)  <6 months >=6 months  At 2 years (17/55)  At 5 years (31/68)  Professional delay  Laryngeal  =>6 months vs <6 months.  HR of 3.5 | 0.041  0.12  0.50  0.28  0.72  0.62  0.36  0.38  0.31  0.073  0.80  0.35  0.67  0.99  0.96  0.23  0.44  0.039  0.25  0.32  0.89  0.71  0.21  0.35  0.51  0.14  0.089  0.21  0.16  0.18  0.58  0.73  0.014  0.023  <0.001  <0.001 | 1.8 | 6.9 | In pharyngeal cancer, patient delay longer than 2 months showed a trend towards worsened survival (mean survival time 28 versus 20 months in patient delay of less and over 2 months, respectively, P = 0.073). In laryngeal cancer, the impact of delay was significant: the cut-off point which made the difference in survival was 3 months in patient delay (mean survival time 48 months versus 43 months, P = 0.039) and 6 months in professional delay (22 versus 17 months, P < 0.001). In tongue cancer the impact of delays on survival were insignificant and often paradoxical: shorter delays showed a trend towards impaired survival. In laryngeal cancer, patient delay of less than 3 months resulted in an absolute reduction of 24% in 5-year death rate. The absolute difference in death rate was as high as 37% between laryngeal cancer patients with professional delay of less than or more than 6 months | Not undertaken |  |
| Teppo (2009) | Fisher’s exact test for categorical data Mann–Whitney U-test for comparing medians Independent samples t-test (comparing means) in cases of continuous data Max tumour diameter on MRI (cm) Patient delay (<6 mth vs =>6 mth)   Professional delay (<1 yr vs =>1 yr)  Total delay (<2 yrs vs =>2 yrs)  Pre-treatment morbidity [n(%)]  Hearing loss  Patient delay (<6 mth vs =>6 mth)  Professional delay (<1 yr vs =>1 yr)  Total delay (<2 yrs vs =>2 yrs)  Tinnitus  Patient delay (<6 mth vs =>6 mth)  Professional delay (<1 yr vs =>1 yr)  Total delay (<2 yrs vs =>2 yrs)  Vertigo  Patient delay (<6 mth vs =>6 mth)  Professional delay (<1 yr vs =>1 yr)  Total delay (<2 yrs vs =>2 yrs)  Post-treatment morbidity [n(%)]  Deafness  Patient delay (<6 mth vs =>6 mth)  Professional delay (<1 yr vs =>1 yr)  Total delay (<2 yrs vs =>2 yrs) Tinnitus  Patient delay (<6 mth vs =>6 mth)  Professional delay (<1 yr vs =>1 yr)  Total delay (<2 yrs vs =>2 yrs) Vertigo  Patient delay (<6 mth vs =>6 mth)  Professional delay (<1 yr vs =>1 yr)  Total delay (<2 yrs vs =>2 yrs) Headache/Pain  Patient delay (<6 mth vs =>6 mth)  Professional delay (<1 yr vs =>1 yr)  Total delay (<2 yrs vs =>2 yrs) Facial weakness  Patient delay (<6 mth vs =>6 mth)  Professional delay (<1 yr vs =>1 yr)  Total delay (<2 yrs vs =>2 yrs) | Mean 1.9 vs 1.8 Median (range) 1.6 (0.8–4.2) vs 1.8 (0.3–4.0) Mean 1.9 vs 1.8 Median (range) 1.8 (0.3–4.0) vs 1.4 (0.5–4.2) Mean 1.9 vs 1.8 Median (range) 1.8 (0.3–4.0) vs 1.6 (0.3–4.2)   22 (63) vs 13 (54) 24 (60) vs 11 (58) 21 (60) vs 14 (58)  19 (54) vs 12 (50) 21 (53) vs 10 (53) 19 (54) vs 12 (50)  13 (37) vs 9 (38) 15 (38) vs 7 (37) 13 (37) vs 9 (38)  23 (92) vs 16 (94) 25 (93) vs 14 (93) 20 (91) vs 19 (95)  2 (8) vs 3 (18) 4 (15) vs 1 (7) 3 (14) vs 2 (10)  9 (36) vs 4 (24) 10 (37) vs 3 (20) 9 (41) vs 4 (20)  4 (16) vs 3 (18) 4 (15) vs 3 (20) 4 (18) vs 3 (15)  11 (44) vs 11 (65) 15 (56) vs 7 (47) 13 (59) vs 9 (45) 0.21 | 0.66 0.65  0.72 0.31  0.79 0.60   0.5 0.6 0.58  0.43 0.53 0.43  0.55 0.54 0.55  0.6 1 0.53  0.33 0.4 0.53  0.29 0.21 0.11  0.62 0.49 0.53  0.17 0.39 0.21 | n/a | n/a | The diagnostic delays, when dichotomised, had no impact on the maximum tumour diameter at the time of diagnosis or on the pre- or post-treatment morbidity. The differences were equally insignificant, both clinically and statistically, with all the other cut-off points studied. Moreover, the diagnostic delays had no impact on tumour size at the time of diagnosis or on pre- or post-treatment morbidity, even when analysed separately for intra- and extracanalicular tumours | Not undertaken |  |
| Tokuda (2009) | Student’s t-test was used for comparing the mean values of symptom-to-visit intervals between patients with and without metastasis    Multivariable-adjusted logistic regression analysis used for analysing possible association between symptom-to-visit intervals and presence of metastasis adjusted for age and gender | Metastasis n(%) = 46 (15.2%) Symptom-to-visit interval, Mean (SD)= 87.4 (139.6) No metastasis n(%) = 257 (84.8%) Symptom-to-visit interval, Mean (SD)= 160.3 288.9 Not performed for individual head and neck cancer site | p<0.01   Not reported | n/a | n/a | The longer interval was significantly associated with a lower likelihood of distant metastasis.  A short interval was associated with a distant metastasis in patients with head and neck cancer  Head and neck cancers are among the types with an interval of months to years | Not undertaken |  |
| Tromp (2005) | Univariate logistic regression analyses Tumour advancement (T1-2/T3-4) vs: Patient delay <1 month (87/33) 1–3 months (60/33) =>3 months (46/32) Referral delay <1 month (107/50) 1–3 months (49/28) =>3 months (35/18) Specialist delay <1 month (56/26) 1–3 months (116/62) =>3 months (35/10) Total delay <1 month (52/14)  1–3 months (110/59) =>3 months (45/26) Stepwise multiple logistic regression analysis. Those factors with a univariate significance of p Tumour advancement (T1-2/T3-4) vs: Patient delay 1–3 months =>3 months The same analyses were carried out to estimate the odds ratios for being diagnosed with advanced stage cancer (stage III or IV) according to the study variables. The various forms of delay were not related to tumour stage | OR 1.00 (Ref.) OR 1.45 OR 1.83  OR 1.00 (Ref.) OR 1.22 OR 1.10  OR 1.00 (Ref.) OR 1.15 OR 0.62  OR 1.00 (Ref.) OR 1.99 OR 2.15     OR 2.00 OR 3.47    Not reported | p=0.049  Non-significant p value not reported Non-significant p value not reported p=0.044       p=0.056 p=0.001    Not reported | 0.81 1.00  0.69 0.57   0.66 0.27  1.02 1.00     0.98 1.62    n/a | 2.60 3.35  2.17 2.13   2.01 1.43  3.89 4.60     4.06 7.44    n/a | The multivariate model demonstrates that patients with patient delay between 1 and 3 months have twice, and patients with a patient delay >3 months have 3.5 the odds for an advanced tumour of those patients with a patient delay  The various forms of delay were not related to tumour stage and there was no associations between different forms of delay and advanced stage cancer | Not undertaken |  |
| Vernham (1994) | Spearman's rank correlation (Rs) Tumour size Patient delay Professional delay TNM Stage Patient delay Professional delay Histological scores Patient delay Professional delay Survival by Kaplan-Meier and log rank test The corrected survival was analysed according to the quartiles of total delay with a test for trend, but no correlation was found | Rs not given Rs-0.22  Rs not given Rs not given  Rs not given Rs not given  Not reported | Non-significant p=0.005  Non-significant  Non-significant | n/a | n/a | Non-significant There is no statistically significant difference between the duration of symptoms in patients with 'early' and those with 'advanced' disease | Not undertaken |  |
| Wildt (1995) | Spearman's rank correlation (Rs) Tumour size Patient delay Professional delay TNM Stage Patient delay Professional delay Histological scores Patient delay Professional delay Survival by Kaplan-Meier and log rank test The corrected survival was analysed according to the quartiles of total delay with a test for trend, but no correlation was found | Rs not given Rs-0.22  Rs not given Rs not given  Rs not given Rs not given  Not reported | Non-significant p=0.005  Non-significant  Non-significant  Non-significant | n/a | n/a | There was no correlation between patient delay and disease status at the time of diagnosis, nor was there any correlation between total delay and the corrected survival. Information about patient delay and total delay obtained at the diagnosis of oral squamous cell carcinoma does not seem to be clinically applicable to categorisation or prognostication of the disease | Not undertaken |  |
| **Brain/CNS (Central Nervous System)** | | | | | | | |  |
| Balasa (2012) | Comparison of complaints at symptom onset with complaints at the time of diagnosis. Post-operative complications. (T-student test) | There was a significant increase of sensory and motor function impairment, and a decrease in pain-related complaints suggesting slowly progressive neurological deterioration for patients. | Not reported | Not reported | Not reported | There was a progressive neurological deterioration from the symptom onset and symptom diagnosis. The short-term post-operative complications were transitory neurological deterioration in 57% of cases. In the long-term there were no recurrences in the follow up period (29±18.6 months, min. 6 months, max. 57 months). | Not reported |  |
| **Melanoma** | | | | | | | |  |
| Baade (2006) | Multiple linear regression, Spearman rank: relationship between diagnostic delay and melanoma thickness | All respondents: -0.17 | Non-significant | -0.38 | 0.05 | There seems to be no positive association between melanoma thickness and TTD on a population basis | < 12 month delayer cut-off, pre-presentation, post-presentation |  |
| Carli (2003) | Multivariate analysis:relationship between patient’s and doctor’s delay and melanoma thickness | Patient delay >3-months v ≤ 3 months OR 1.12  Physician delay >1-month v ≤ 1 month OR 0.59 | n/a | 0.78 0.38 | 1.60  0.91 | No significant effect of patient's delay of longer than 3 months was found on melanoma thickness | Not undertaken |  |
| Cassileth (1982) | Correlation coefficient :correlation between time to diagnosis and melanoma thickness | R = -0.06 | Non-significant | n/a | n/a | Study demonstrates that there is no correlation between TTD and the biologic state of disease (i.e. Superficial spreading melanoma) as reflected by thickness | Not undertaken |  |
| Helsing (1997) | Student’s t-test, Spearman rank correlation: correlation between delay and Breslow thickness | R = 0.246 (inverse relationship) | P=0.068 | n/a | n/a | An inverse relationship (n/s at the p=0.05 level) may be explained by the variable biology of cutaneous malignant melanoma | Analysis excluding incidentally-diagnosed melanomas (no difference in results) |  |
| Krige (1991) | Correlation coefficient: correlation between time to diagnosis and melanoma thickness | R = 0.027 | Non-significant | n/a | n/a | No correlation was found between delay in diagnosis and ultimate thickness of lesion | Sub-group analysis done according to histogenetic type: SSM, NM (0.2087, p<0.05), LMM, ALM. |  |
| Metzger (1998) | Mann Whitney U test, Kaplan Meier: relationship between physician delay and tumour thickness, stage and survival | Correct diagnosis vs misdiagnosis  Median tumour thickness (mm) 1.5 vs 5.0  Correct diagnosis vs misdiagnosis  Stage la/lb: 14 vs 1  Stage lla/llb: 21 vs 6  Stage llla: 1 vs 0  Stage lllb: 1 vs 4  Stage lV: 0 vs 2  Correct diagnosis vs misdiagnosis  5- year survival rate(%): 68.9 vs 15.4 | Descriptive | n/a | n/a | Professional delay in diagnosis influences the patients prognosis | Not undertaken |  |
| Montella (2002) | Multiple logistic regression: relationship between diagnostic delay and tumour thickness | Odds ratio < 3 month: 1 (Reference) 3-6 months: 1.0 > 6 months: 1.5 | P=0.9  P=0.09 | 0.6 0.9 | 1.7 2.4 | The relation between diagnostic delay and thickness is not direct or causal, it is often attributed to chance and it is correlated to some sociocultural variables, first physician visited, to mole sites and to other personal attitudes | Not undertaken |  |
| Richard (1999) | Spearman Rank correlation:  correlation between delays in diagnosis and tumour thickness  Kruskal-Wallis test: relative risk of increased Breslow thickness, mean/median | From first noticing lesion to treatment:  R = -0.004  From noticing a lesion to identifying as suspicious:  R = -0.20  Relative risk:  < 4 months vs. > 4 months (D1-D2): 1.00/1.80 vs 1.90/3.44  < 2 months vs. > 2 months (D2-D3): 1.70/3.00 vs  1.00/2.00 | P=0.90  P<.001  P= 0.003  P= 0.001 | n/a | n/a | No correlation was found between melanoma thickness and physicians delay. There is a negative correlation between tumour thickness and the delay to seek medical attention .  Our data underline that the Breslow thickness of melanoma results not only from delay in diagnosis but also from the biological aggressiveness of the tumour. | A correlation was shown between Breslow thickness and the delay in the D1-D2 when the interval was no greater than 5 years. No correlation was found in the sub-group of those > 5 years. This group often included those with a pre-existing nevus. |  |
| Schmid-Wendtner (2002) | Association between period of delay and tumour thickness | Descriptive: long periods of patient or professional delay were not associated with tumour thickness. |  |  |  | Data did not indicate an association between the period of delay and tumour thickness. Only in patients with a very high tumour thickness did the periods of delay tend to be prolonged | Not undertaken |  |
| Temoshok (1984) | Correlation coefficient: correlation between delay and tumour Breslow thickness and Clark’s level (1-5) | Superficial spreading melanoma (SSM):  Thickness: r = 0.17  Clark level: r = 0.27  Nodular melanoma (NM):  Thickness: r = 0.49  Clark level: r= 0.04 | Non-significant  P=0.06  P=0.02  Non-significant | n/a | n/a | Delay in seeking medical attention for suspicious lesions is strongly associated with poorer prognosis of malignant melanoma. | Sub-group analysis with coincidentally diagnosed patients removed. Statistically significant result for correlation between delay and thickness for both NM and SSM. |  |
| Tørring (2013) | Kaplan-Meier method, conditional logistic regression to estimate ﬁve-year mortality odds ratios as a function of the diagnostic interval using restricted cubic splines and adjusting for comorbidity, age, sex and type of cancer. | Mortality rates (MR%) and crude/adjusted odds ratios (OR) for the 1st and 4th compared with 2nd and 3rd diagnostic interval quartiles (DIQ) for patients with a) alarm or serious symptoms and b) vague symptoms.  Alarm or serious symptoms  DIQ MR% Crude OR Adjusted OR  1^st^ 20.0 1.00 1.83  2^nd^ + 3^rd^ 20.0 1 1  4^th^  26.3 1.43 1.88  Vague symptoms  DIQ MR% Crude OR Adjusted OR  1^st^ 10.0 Comparison unjustified  2^nd^ + ^3rd^ 27.8  4^th^ 22.2 |  | 0.26/0.37  Reference  0.40/0.44 | 3.83/8.96  Reference  5.15/8.00 | In patients with alarm or any serious symptoms, mortality grew the longer the diagnostic intervals in those 40% from this group who experienced the longest delays. In patients with alarm or any serious symptoms, having very short diagnostic intervals was also associated with a high mortality. Inversely, in patients presenting with vague symptoms, we saw much longer diagnostic intervals, the same survival probability and an opposite, concave trend between the length of the diagnostic interval and mortality. However, the latter association was not statistically signiﬁcant and the association did not apply to prostate cancer. | Not reported |  |
| **Non-melanoma skin** | | | | | | | |  |
| Alam (2011) | McNemar test. Association of increase in tumour size with delay in presentation for treatment. | Size of lesion when first noticed:  ≤1 month/> 1 month;  Small as a pinhead 33(13%)/53(9%);  About the size of a pimple 178(68%)/424(75%);  Dime size 36(14%)/72(13%);  Nickel size 6(2%)/13(2%);  Bigger than a nickel 8(3%)/5(1%);  Size of lesion when first shown to a doctor: Small as a pinhead 32(12%)/26(5%);  About the size of a pimple 171(66%)/384(68%);  Dime size 40(15%)/117(21%);  Nickel size 8(3%)/24(4%);  Bigger than a nickel 8(3%)/13(2%). | Not reported | Not reported | Not reported | On average, skin cancers grew while patients were waiting, with the average lesion enlarging from pimple size (2-3 mm) to between pimple and dime size (10 mm). There was a significant increase in tumour size from the time when tumours were noticed by patients to the time when patients presented for treatment. | Not reported |  |
| Renzi (2010) | Association of total delay and size of squamous cell carcinoma. Chi squared test; univariate and multivariate analyses. | Univariate:  Short delay: size ≤2 cm/>2 cm; 181/40.  Long delay (more than 18 months): size ≤2 cm/>2 cm; 57/29.  Multivariate:  Short delay: size >2cm; OR 1.  Long delay: size >2cm; OR 2.28. | P=.001  P=.05 | 1.00 | 5.19 | Univariate analysis on the total study sample shows that larger SCCs are associated with total delay longer than 18 months. Multivariate analysis showed an association between long total delay and size to be of borderline significance. | Association of delay and invasive and in situ SCC. Long total delay was associated with SCC size only for invasive SCC. |  |
| Tokuda (2009) | Comparison of the mean symptom to visit intervals between patients with and without metastasis: (Student's t-test) | 133.3/340.5 | p<0.05 |  |  | The risk for distant metastasis is higher among patients with solid tumours and a short interval of symptom onset to clinical presentation. | Not undertaken |  |
| **CTYA (Children, Teenagers and Young Adults)** | | | | | | | |  |
| **Brain/CNS (Central Nervous System)** | | | | | | | |  |
| Brasme (2012) | Kruskal-Wallis test.  Kaplan-Meier, Log rank: relationship between time to diagnosis and survival. Cox model, logistic regression. | Stage:  Relation between time to diagnosis (median) and tumour stage:  T1 or T2 n=49; 75 days.  T3A n=25; 62 days.  T3B n=64; 61 days.  T4 n=28; 84 days.  Survival:  Univariate analysis:  Time to diagnosis: median (65 days) n=83;  10 year survival = 47%;  ≥median n=83;  10 year survival = 60%.  Multivariate:  Relation between time to diagnosis (median) and tumour volume:  Small n=55; 45 days. Intermediate n=55; 69 days. Large n=56; 86 days.  Relation between time to diagnosis (median 87 vs 72 vs 72 days respectively) and neurological disability (n=96): normal 30%; moderate and unilateral dysmetria without functional consequence 36%; neurological disability 33%.  Relation between time to diagnosis and IQ Score of 83% of 96 survivors (n=80). | P=0.35  P=0.02  P=0.17  P=0.002  p>0.2  P=0.01 | Relative risk (95% CI) 1.8.  1.2 Adjusted relative risk (95% CI)1.5  0.8 | 2.8  2.5 | We did not find a statistically significant association between time to diagnosis and local extension.  The survival of patients with a long time to diagnosis was significantly better than that of patients with a short pre-diagnostic interval but this was no longer significant after adjustment for confounding factors.  A long pre-diagnosis interval was associated with a larger tumour volume on univariate analysis but not after adjustment. No significant relation was found between the time to diagnosis and neurological disability. The IQ score was significantly associated with the pre-diagnosis interval, both after linear regression following transformation and after adjustment for covariables (p<0.05). | In the 62 patients with metastases, a long pre-diagnosis interval was associated with a higher T stage, infiltration of the fourth ventricle floor, and incomplete surgical resection; it nonetheless did not influence survival significantly in this subgroup. |  |
| Crawford (2009) | Comparison of overall survival for those with a symptom duration of  > 6 months vs < 6 months  (Kaplan-Meier, t-test) Association of grade with duration of symptoms (statistical test and outcome measure unclear)  High grade tumours- average duration  Low grade tumours – average duration | 1.65 months, range 0.25-7 months 11.1 months, range 0.25-60 months | p=0.91  p=0.05 |  |  | Duration of symptoms did not affect overall survival  Shorter duration of symptoms is associated with higher grade tumours | Not undertaken |  |
| Halperin (2001) | Comparison of median symptom duration in patients with:  low-stage disease  high-stage disease  (t-test)  Correlation between symptom duration and disease stage (logistic regression) | 8  4  Data not presented | 0.01   Not reported | n/a | n/a | The duration of symptoms is significantly less for patients with high stage disease than those with low stage disease.  Disease stage in medulloblastoma is correlated inversely with duration of symptoms | Not undertaken |  |
| Kameda-Smith (2013) | Correlation co-efficient: pre-diagnostic interval and survival. | PSI and survival:  r value: 0.084 | P=0.503 |  |  | PSI was not significantly correlated with outcome. | Not reported |  |
| Kukal (2009) | Comparison of 10 year overall survival/progression free survival probability for patients (n=315) with PSI of: < 20 days 20-59 days 60-179 days ≥ 180 days (Chi squared, log rank) Comparison of 10 year overall survival/progression free survival probability for patients (n=234) with doctor's delay of: < 1 day 1-14 days 15-70 days > 70 days (Chi squared, log rank) | 71%/49% 61%/39% 66%/43% 86%/61%     67%/53% 64%/49% 67%/40% 78%/60% | p<0.001/p=0.029        p=0.24/p=0.64 | 59/37 50/28 55/32 78/50     55/41 51/35 53/26 65/47 | 85/64 73/54 80/60 95/74     81/68 79/67 83/60 90/75 | In the group with the longest PSI the survival probability was the highest. The PSI correlated inversely with progression free and overall survival.  The survival probability was highest in the group with the longest doctor's delay but these differences were not significant | Not undertaken |  |
| Sethi (2013) | Stage: Chi squared test: delayed diagnosis and likelihood of disseminated disease.  Survival: Kaplan-Meier, Cox regression, log rank: association of delayed diagnosis and progression free survival (PFS). | Likelihood of disseminated disease:  Delayed diagnosis: 43%  Non-delayed: 6%  Survival:  Delayed diagnosis: 93%  Non-delayed: 83% | P=.007  P=.20 | Not reported | Not reported | Patients with delayed diagnosis were more likely to have disseminated disease at diagnosis.  At a median follow-up of 34 months from the date of diagnosis, progression-free survival (PFS) at 3 years was similar in the 2 groups of patients. | In a subgroup analysis of patients with pure germinoma histology, 3-year PFS was similar in patients with delayed diagnosis and those without delay (90% vs 90%; p= .60). One patient with a non-disseminated NGGCT whose diagnosis was not delayed died of disease after local recurrence at 15.3 months after diagnosis. |  |
| **Head and Neck** | | | | | | | |  |
| Butros (2002) | Log-transformed t test and the Wilcoxon nonparametric test | Eye loss in bilateral disease  Choroidal extension Survival | p=0 .17  Not reported Not reported | n/a | n/a | A trend toward longer delays being associated with eye loss in patients with bilateral retinoblastoma was noted but did not reach statistical significance The delays to diagnosis in patients with choroidal extension were not significantly different from delays in the patients without aggressive disease at diagnosis Patient survival was not affected by the delay to diagnosis | Not undertaken |  |
| Erwenne (1989) | Kaplan-Meier analysis for early versus late referrals against survival and  Cox-Mantel test for univariate analyses with two tailed p values   Logit estimates of OR according to Woolf technique for stage | Early referrals (n=80) Mean survival time (months)=101.0 3 year survival rate=81.5% Late referrals (n=73) Mean survival time (months)=59.6 3 year survival rate=44.3% Interval <=6 months EO/IO ratio=20/67, OR 1.0 (ref) Interval 7-12 months EO/IO ratio=17/13, OR 4.3 Interval >12 months EO/IO ratio=30/11, OR 8.7 | <0.0001         Not reported  Not reported | n/a         1.7  3.6 | n/a         11.6  23.6 | The risks of having extraocular disease in late referrals was found to be almost nine times higher than that seen among patients with early referrals (<=6 months) | Not undertaken |  |
| Goddard (1999) | Mann–Whitney and Kruskall–Wallis tests and Spearman’s rank correlation | Not reported | n/a | n/a | n/a | There was no significant difference in overall lag time for enucleated compared with non-enucleated eyes. The need for enucleation was not influenced by diagnostic delay Overall lag time for patients requiring adjuvant therapy (27 weeks, range 2–61) was significantly longer than those patients with no evidence of local tumour invasion (8 weeks, range 1–94) | Not undertaken |  |
| Wallach (2006) | Logistic regression analysis Wald’s test used to calculate p values (2-sided tests) t test or 1 way analysis of variance to compare means | <1 month RR 1.00 1–3 months RR 1.41  3–6 months RR 1.77 >6 months RR 8.09 | p=0.518 p=0.306 p=0.005 | 0.5 0.59 1.86 | 4.00 5.25 35.23 | Delayed interval (DI) was statistically significant factor influencing diagnosis of advanced stage (Murphree group E) | Not undertaken |  |
| **Leukaemia** | | | | | | | |  |
| Lins (2012) | Survival:  Kaplan-Meier, log rank test.  Early death:  OR with 95% CI, significance level 5%. Chi square test. | No delay: 67% (n=130)  Delay: 64%(n=156)  Early death:  No delay: 14, 10.9% (n=130)  Delay: 11, 7.0%(n=158)  No early death:  No delay: 116, 89.1% (n=130);  Delay: 147, 93.0 %( n=158). OR 0.62. | Non-significant  P=0.35 | 0.27 | 1.41 | There was no association between delayed diagnosis and event free survival at 5 years.  In the present study, no differences in early mortality for children with and without delays in diagnosis were observed. | Not reported |  |
| Marwaha (2010)a | Students t test: Comparison of symptom to presentation interval (SPI) with patients initially misdiagnosed with JRA and those not. Kaplan-Meier; Log rank: Comparison of overall and event free survival for patients with and without initial misdiagnosis as JRA. Cox multivariate regression model: prognostic variables for survival. | SPI (mean, days) of those misdiagnosed/not misdiagnosed: 91.0 ± 20/54.6 ± 4.0.  Comparison of OS and EFS for patients with and without initial misdiagnosis as JRA.    Prognostic variables for survival: OR=1.66. | P=0.001  P=0.06  P=0.006 | 1.316 | 2.105 | The SPI was significantly higher in the patients who were initially diagnosed. Initial misdiagnosis of JRA was not a significant determinant of outcome. SPI was a significant predictor for survival outcome. | Not reported |  |
| Marwaha (2010)b | Chi square test, Kaplan-Meier method. Multivariate analysis. | Symptom diagnosis interval (mean), (days).  Deaths: 53.9 ± 8.3  Survivors and cured: 39.6 ± 7.9.  Multivariate | P=0.049  P=0.001 |  |  | Longer symptom diagnosis interval (SDI) was associated with an increased risk of death. Multivariate analysis showed that longer SDI significantly influenced death. | Not reported |  |
| Wahl (2012) | 2 x 3 exact Chi Square tests.  Survival (all, high risk and standard risk patients) and treatment delay.  Relapse (all, high risk and standard risk patients) and treatment delay.  Cox regression analysis models. | Death and treatment delay of 2/3/4 or more days:  All patients: 5 (19.2%)/5 (38.5%)/0 (0%)  High risk: 10 (11.9%)/ 4 (11.1%)/6 (12.5%)  Standard risk: 15 (15.5%)/13 (29.5%)/2 (3.8%)  Relapse rate and treatment delay of 2/3/4 or more days:  All patients: 5 (19.2%)/4 (30.8%)/ 1 (7.7%).  High risk: 16 (19.0%)/ 7 (19.4%)/ 9 (18.8%).  Standard risk: 17 (17.5%)/ 13 (29.5%) 4 (7.5%).  Bacteremia and treatment delay of 2/3/4 or more days:  Positive cultures: 5(19.2%)/4 (4.8%)/11/(12.9%).  Admission to ICU and treatment delay of 2/3/4 or more days:  ICU transfer: 11 (42.3%)/21 (25.0%)/20 (20.6%).  Treatment delay and risk of relapse: HR 0.98 | P=0.55  P=0.06  P=0.19  P=0.94  P=0.58  P=0.23  P=0.07  P=0.08  p=0.80 | Not reported  0.18 | Not reported  1.78 | There was no association between death and treatment delay for all patients, HR patients or SR patients.  There was no association found between relapse rate and treatment delay days for all patients and within each risk group. There was no increased incidence of bacteremia associated with an increased time to treatment initiation, though a higher proportion of patients with bacteremia were in the group with the shortest treatment delay. Similarly, there was a trend toward a higher percentage of ICU admissions in patients with shorter treatment delays.  Treatment days was not an independent predictor of relapse. | Not reported |  |
| **Connective tissue** | | | | | | | |  |
| Bacci (1999) | Proportion of patients with metastatic disease among those with a less-than 2 month interval to diagnosis (32%) vs a more-than 4 month interval (12%)  (Chi-square test) | Not reported | p<0.0001 | n/a | n/a | The time to diagnosis was not found to be positively associated with the stage of disease. This probably reflects inherent biological differences in the aggressive behaviour among subgroups of patients, that might be implicated in the clinical course and the propensity to respond to adjuvant treatment | Not undertaken |  |
| Ferrari (2010) | Kaplan-Meier; Cox model | Multivariate analysis of sarcoma-specific mortality HR (95% CI) for symptom interval, months:  ≥1, <3 vs <1: HR 1.4  ≥3, <6 vs <1: HR 1.9  ≥6, <12 vs <1: HR 2.4  >12 vs <1: HR 3.6 | p=0.002 | 0.7  1.0  1.2  1.7 | 2.6  3.7  4.9  8.0 | The risk of death tended to increase significantly the longer the symptom interval (P=0.002). Analysis of the interaction of symptom interval and histological type showed a more complex pattern, with a longer time interval for tumours in the extremities. | Not reported |  |
| Simpson (2005) | Distribution of Enneking stage based on duration of patient delay (chi-square test) | Not reported | p=0.1 | n/a | n/a | The length of symptom duration did not affect presenting Enneking stage significantly, although there was a trend toward increased mortality and increased local recurrence in those with high-stage tumours | Not undertaken |  |
| Yang (2009) | Correlation between duration of symptoms (30 days)before first consultation and: With or without metastases Good chemonecrosis Limb salvage surgery Relapse Death Correlation of all the above using 60 days | 24%/24% 49%/43% 93%/81% 41%/25% 21%/35% No difference in outcome variables | p not stated p=0.8 p=0.3 p=0.5 p=0.3 |  |  | There were no significant correlations with respect to the feasibility of limb salvage surgery, chemonecrosis factors, relapse and overall survival rates and duration of symptoms before first consultation. | Not undertaken |  |
| **Solid tumours** | | | | | | | |  |
| Loh (2012) | Stage:  Two tailed t-test. Association of delay and disease stage.  Survival:  Cox regression. Kaplan Meier. Association of total delay and event free survival. | Stage: no association  Survival:  Total delay and event free survival;  < median 5.3 weeks (EFS 81.4 ± 4.4 months)  > Median 5.3 weeks (EFS 98.8 ± 6.2 months). | P=0.945  p=0.737 (Adjusted for delay less or more than the overall median) |  |  | Disease stage was not associated with delay.  The logarithm of total delay was not significantly predictive of EFS. Kaplan-Meier analysis showed no difference in EFS for tumours with total delay less or more than the overall median of 5.3 weeks. | Not reported |  |
| **Leukaemia** | | | | | | | |  |
| Bertoli (2013) | Restricted cubic spline method Differences in survival functions were tested using the log rank test | TDT in days (median survival in months):  1 (12.0); 2 (18.2); 3-4 (21.2); 5-6 (27.7); 7-10 (15.5); >10 (21.6). | P=.1048 | Not reported | Not reported | TDT was not significantly associated with early death in both adjusted and non-adjusted analysis. | Not reported |  |
| Friese (2011) | Cox regression was used to estimate the impact of diagnosis delays and receipt of flow cytometry within 30 days of CLL diagnosis on overall survival. Covariate selection was accomplished by including those variables significantly associated with delay in our prior models plus socio-demographic factors. | Delay and overall survival HR, 1.11 | Non-significant | 0.99 | 1.25 | We did not observe a significant effect of delay between sign or symptom and diagnosis on overall survival. The median survival time for the full sample was 10.4 years. | Not reported |  |
| Prabhu (1986) | Correlation between duration of symptoms and survival  Comparison of median (actuarial) survival (months) for those with a duration of symptoms:  Up to 3 months  > 3 months | 56.72  49.43 | Non-significant | n/a | n/a | Duration of symptoms did not correlate with survival | Not undertaken |  |
| **Lymphoma** | | | | | | | |  |
| Foulc (2003) | Relative risk of longer interval before diagnosis (Likelihood ratio test): Multivariate analysis (Cox Regression analysis): | 0.8899  Not reported | p=0.0566  Not reported | n/a | n/a | Poor prognosis associated with short interval before diagnosis and the interval before diagnosis was considered an independent risk factor in multivariate analysis.  A short interval before diagnosis appeared to be a negative prognostic factor for survival | Not undertaken |  |
| Jacobi (2008) | Association between 5 year/10 year survival and median symptom duration: ≤ 3 months > 3 months | 83.3%/66.2% 81.4%/69.7% |  |  |  | The duration of symptoms before diagnosis did not have an important impact on overall survival. | Not undertaken |  |
| Kim (1995) | Univariate analysis (Gehan test): Comparison of survival rates for those diagnosed in less than 10 years vs 10 years or more  Comparison of survival rates for those diagnosed in less than 2 years vs 2-10 years Multivariate analysis (proportional-hazards regression method): Hazard ratio for those with diagnosed in less than 10 years vs 10 years or more | Not reported    Not reported      1.56 | p=0.055    p=0.74      p=0.12 | n/a | n/a | Survival was more favourable for the group with the longer symptom duration (10 years) and showed no difference  Our results indicate that the patients with longer symptom duration have a tendency for a better survival outcome, but only when the symptom period was exceedingly long (>10 years) | Not undertaken |  |
| Maguire (1994) | Comparison of survival curves for quintile 1 of symptom-to-diagnosis-interval (i.e. 20% of the shortest intervals) vs higher quintiles (log-rank test) Comparison of SDI for various stage levels (Local, Regional, Disseminated, Unspecified) (Kruskall-Wallis test) | Not reported     Not reported | p=0.781     p=0.427 | n/a | n/a | No association between symptom-to-diagnosis interval and survival | Not undertaken |  |
| Norum (1995) | Correlation between delay in diagnosis and (Pearsons coefficient): Survival Advanced clinical stage | Not reported | Non-significant | n/a | n/a | There was no correlation between delay in diagnosis and clinical stage, or death from the disease. The nature of lymphocyte predominant HD, usually presenting in stage I and II and located in lymph nodes without affecting adjacent structures, may be the cause of a prolonged delay in diagnosis in this subgroup. Clinical symptoms and stage distribution are probably more influenced by the aggressiveness of the malignant disease rather than delayed diagnosis | Not undertaken |  |
| **Myeloma** | | | | | | | |  |
| Friese (2009) | Effect of delay on likelihood of complications (Odds ratio) | OR 0.91 | Not reported | 0.80 | 1.03 | There was no evidence of a direct relationship between delayed diagnosis and skeletal or renal complications. | Not undertaken |  |
| Kariyawasan (2007) | Survival from diagnosis: Comparison of survival rates (overall survival) for those diagnosed 0-3 months vs 3-6 months vs >6months (log-rank test)  Comparison of survival rates (disease-free survival) for those diagnosed 0-3 months vs 3-6 months vs >6months (log-rank test)  Survival from onset of symptoms: Comparison of survival rates (overall survival) for those diagnosed 0-3 months vs 3-6 months vs >6months (log-rank test)  Comparison (disease-free survival) for those diagnosed 0-3 months vs 3-6 months vs >6months (log-rank test)  Comparison of proportion of patient diagnosed 0-3 months in Stage I and III vs those diagnosed >6months in Stage I and III (chi-square test) | Not reported | p=0.14    p=0.003     p=0.59    p=0.043     p=0.04 | n/a | n/a | Patients who had a delay of >=6months were more likely to have an increased number of complications, Durie-Salmon stage III and reduced disease-free survival, measured both from onset of symptoms and from time of diagnosis.  The effect of prolonged time to diagnosis on disease-free survival from the onset of the original symptoms implies an impact on the durability of remission achieved after a delay in initial diagnosis and commencement of treatment | Not undertaken |  |
| **Connective tissue** | | | | | | | |  |
| Bacci (2002) | Relative risk of metastasis at presentation of patients diagnosed <=2 months vs >2 months (multivariate) Comparison of proportion of patients diagnosed <2 months with metastatic vs localized disease (univariate) Comparison of (median?) interval between the onset of symptoms and diagnosis in patients with metastatic vs local disease (univariate). | Not reported    56.1% vs 45.2%   2.17 vs 2.54 | Non-significant   p=0.009   p=0.0002 | n/a | n/a | In the current study, the time to diagnosis was not found to be positively associated with the stage of the disease; on the contrary, patients with metastatic tumours generally received diagnoses earlier than did patients with localised disease | Not undertaken |  |
| Nakamura (2011) | Association of time from symptom onset to treatment and cumulative overall survival rates and distant metastasis free survival. Kaplan-Meier method, log rank test.  The survival time was counted from the date of the initial treatment for the primary tumour. Multivariate analysis was performed using Cox proportional hazard model. | Overall 5-year survival rate in the 48 patients treated within the first six months: 77%  Overall 5 year survival rate in the 34 patients who were treated more than 6 months: 59.7%. | P=0.22 | Not reported | Not reported | The current study showed that the short interval between the onset of the initial symptoms and start of treatment has a significant association with an improved survival in patients with primary soft tissue sarcoma. A delay of more than 6 months to treat was identified as the worst prognostic factor in both the univariate and multivariate analysis. | Not reported |  |
| Rougraff (2007) | Hazard ratio (Cox regression model) and correlation (method not reported) between duration of symptoms and: - survival - disease-free survival - size of the tumour at diagnosis | Not reported | Non-significant | n/a | n/a | Greater duration of symptoms did not correlate with lower survival or continuous disease-free survival. This lack of association might be explained by the fact that the timing of primary tumour spread and growth of metastatic disease might be extremely variable.  Metastases at diagnoses was just as likely to occur in patients with the shortest length of symptoms as in patients with longer times to diagnosis far beyond the mean. |  |  |
| Ruka (1988) | Analysis of the impact of symptom duration (treated as a continuous variable) on prognosis (overall survival time) (Cox regression) Estimated 5-year relative survival for symptom duration =6months Analysis using Cox's proportional hazards model for 'size/duration (S/D) ratio' (as a measure of aggressiveness) vs survival time. In a multi-variate proportional hazards model for survival time, the S/D ratio was significantly related to survival * the S/D ratio was dichotomised with a 1.0 cut-off point | Not reported    33% vs 35% | p=0.2490      p<0.0001   p=0.0005 |  |  | Survival time is significantly better for patients with longer duration of symptom rather than maximal size of tumour (S/D <=1) vs. those with shorter duration of symptoms as compared to diameter of tumour (S/D > 1). The S/D ratio, despite shortcomings, reflects dynamic characteristics of the biological behaviour of sarcomas. | The majority of patients (123/180, 68%) with large sarcomas (>5cm) had symptom duration longer than 6 months |  |
| Saithna (2008) | Mean difference in symptom duration in weeks between metastasis and non-metastasis Mean difference in symptom duration in weeks between sub-cutaneous and deep Mean difference in symptom duration in weeks between grade: High, intermediate High, low Intermediate, low Survival non-metastatic (Cox proportional hazards model) Hazard ratio Survival metastatic (Cox proportional hazards model) Hazard ratio | 19.4  35.4  29.6 61.4 31.8  0.998   0.998 | Non-significant  Significant, no p  Significant, no p    p=0.0038  Non-significant | -2.5  16.5  8.9 39.8 7.2  0.997  0.995 | 41.4  54.4  50.3 83.0 56.4  0.999  1.000 | Those patients who present early, and therefore have a shorter duration of symptoms, have a less favourable overall survival. An additional week of symptoms improved the monthly survival rate by 0.2%. This is probably a reflection of the poor prognosis conferred by high-grade disease. We have been unable to demonstrate any significant survival benefit associated with shorter symptom durations even when the effect of more aggressive tumours is accounted for in multivariate analysis. | For patients with deep, high grade tumours >5cm and without metastases at diagnosis n=411, symptom duration had no effect on survival. Hazard ratio 1.0. |  |
| Wurtz (1999) | Association between symptom duration and: Survival Grade Stage Association between the survival of those who had a delay in diagnosis of at least one month and that of the remaining patients | Not reported | p=0.54  Non-significant Non-significant  p=0.62 | n/a | n/a | Symptoms may be present for a long period of time before the patient seeks medical attention. Furthermore, we detected no association between a long (at least one-month) delay in diagnosis. Patients with a high-grade bone sarcoma of the pelvis had a poor prognosis regardless of any delay before an accurate diagnosis was made | Not undertaken |  |
| **Carcinoid** | | | | | | | |  |
| Toth-Fegel (2004) | Correlation between delay of diagnosis and extent of disease (Fishers exact test) Correlation between delay of diagnosis and survival (Fishers exact test) |  | p=0.20  Non-significant |  |  | Linear regression analysis showed no significant correlation between delay of diagnosis and extent of disease score. There was no correlation between any length of delay of diagnosis and death due to carcinoid disease. | Not undertaken |  |
| **Thyroid** | | | | | | | |  |
| Tokuda (2009) | Correlation between delay of diagnosis and extent of disease (Fishers exact test) Correlation between delay of diagnosis and survival (Fishers exact test) | 428.4/169.9 | Non-significant |  |  | The risk for distant metastasis is higher among patients with solid tumours and a short interval of symptom onset to clinical presentation. | Not undertaken |  |
| **Multi-site** | | | | | | | |  |
| Tørring (2013) | Kaplan-Meier method, conditional logistic regression to estimate ﬁve-year mortality odds ratios as a function of the diagnostic interval using restricted cubic splines and adjusting for comorbidity, age, sex and type of cancer. | Alarm or serious symptoms  DIQ MR% Crude OR Adjusted OR  1^st^ 54.2 1.98 1.98  2^nd^ + 3^rd^ 41.3 1 1  4^th^  48.5 1.49 1.49  Vague symptoms  DIQ MR% Crude OR Adjusted OR  1^st^ 52.5 0.53 0.51  2^nd^ + ^3rd^ 63.2 1 1  4^th^ 57.1 0.70 0.67 |  | 1.34/1.34  Reference  1.01/1.01  0.23/0.21  Reference  0.30/0.26 | 2.91/2.91  Reference  2.22/2.22  1.22/1.24  Reference  1.65/1.71 | In patients with alarm or any serious symptoms, mortality grew the longer the diagnostic intervals in those 40% from this group who experienced the longest delays. In patients with alarm or any serious symptoms, having very short diagnostic intervals was also associated with a high mortality. Inversely, in patients presenting with vague symptoms, we saw much longer diagnostic intervals, the same survival probability and an opposite, concave trend between the length of the diagnostic interval and mortality. However, the latter association was not statistically signiﬁcant and the association did not apply to prostate cancer. The study displays the immense complexity and difficulty of diagnosing cancer. Yet, we can infer from the increasing trends in mortality that a few weeks can make a difference – that time matters. | Not reported |  |
